# Recent spread of Varroa destructor virus-1, a honey bee pathogen, in the United States

**DOI:** 10.1038/s41598-017-17802-3

**Published:** 2017-12-12

**Authors:** Eugene V. Ryabov, Anna K. Childers, Yanping Chen, Shayne Madella, Ashrafun Nessa, Dennis vanEngelsdorp, Jay D. Evans

**Affiliations:** 1 US Department of Agriculture, Agricultural Research Service, Bee Research Lab, Beltsville, MD USA; 2 University of Maryland, Department of Entomology, College Park, MD USA

## Abstract

RNA viruses impact honey bee health and contribute to elevated colony loss rates worldwide. Deformed wing virus (DWV) and the closely related Varroa destructor virus-1 (VDV1), are the most widespread honey bee viruses. VDV1 is known to cause high rates of overwintering colony losses in Europe, however it was unknown in the United States (US). Using next generation sequencing, we identified VDV1 in honey bee pupae in the US. We tested 603 apiaries the US in 2016 and found that VDV1 was present in 66.0% of them, making it the second most prevalent virus after DWV, which was present in 89.4% of the colonies. VDV1 had the highest load in infected bees (7.45*10^12^ ± 1.62*10^12^ average copy number ± standard error) compared to other tested viruses, with DWV second (1.04*10^12^ ± 0.53*10^12^). Analysis of 75 colonies sourced in 2010 revealed that VDV1 was present in only 2 colonies (2.7%), suggesting its recent spread. We also detected newly emerged recombinants between the US strains of VDV1 and DWV. The presence of these recombinants poses additional risk, because similar VDV1-DWV recombinants constitute the most virulent honeybee viruses in the UK.

## Introduction

The European honey bee *(Apis mellifera)* is the most commonly managed bee in the world and a key contributor to pollination of food crops and wild plants. The economic impact of animal pollination services worldwide has been estimated at US$235–577 billion (in 2009) annually^1^, with honey bee pollination provide the bulk of these services (e.g., $210 billion in 2005). Overall, pollinator services provide 9.5% of the total agricultural economy^2^. Honey bees and other pollinators are challenged by abiotic and biotic stress factors that cause queen and worker mortality and impact colony health^3,4^ resulting in more than 50% of hives being lost annually since 2006 in the United States^5–8^.

**Figure 1:**
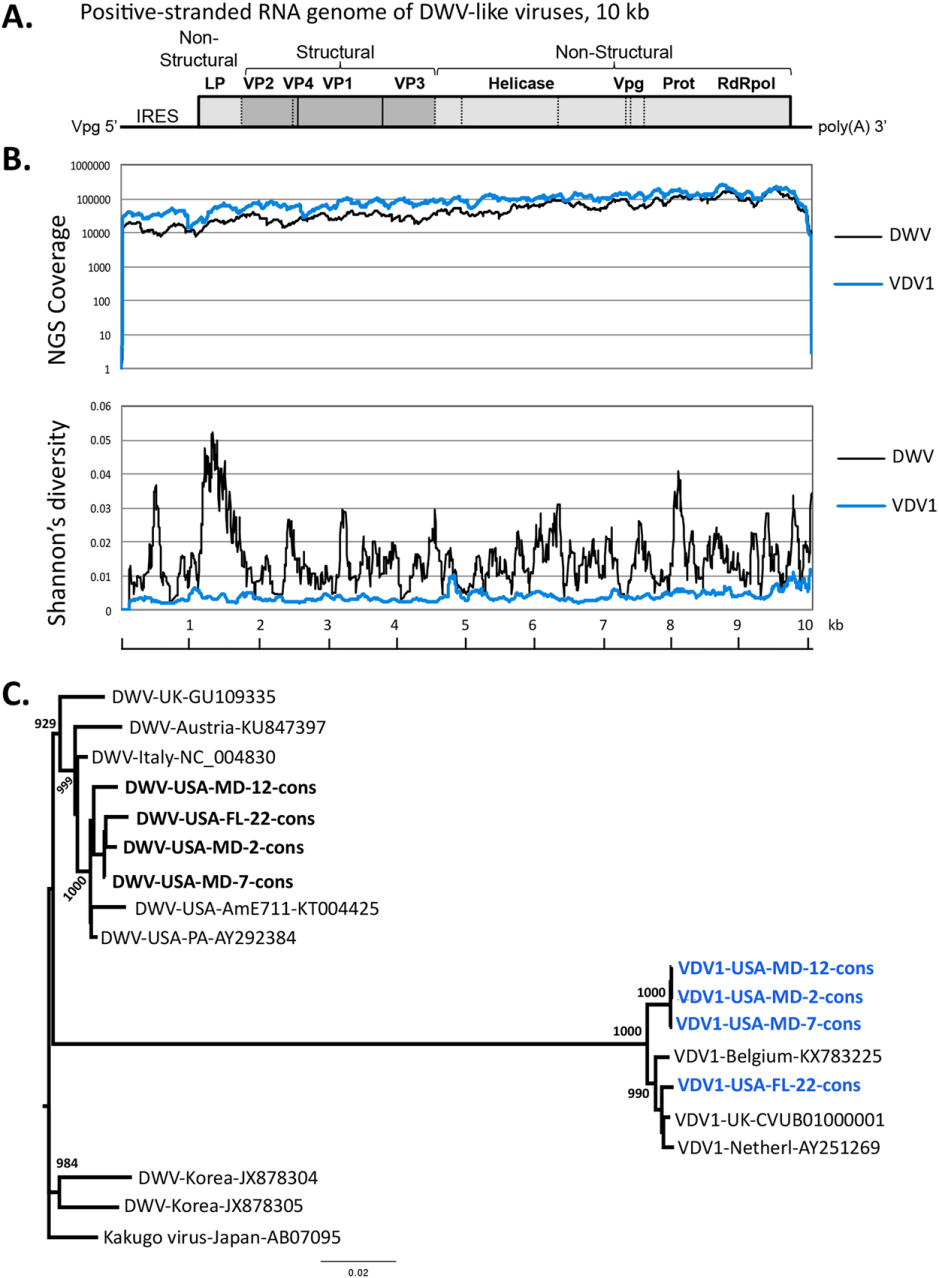
NGS read coverage, Shannon’s diversity index and relationship between DWV-like viruses. (**A**) Genome organization of DWV and VDV1. (**B**) Analysis of an NGS library from a single honey bee pupa (Pupa-No-2-MD). Shown are NGS read coverage and average Shannon’s diversity index profiles (100 nt window) for DWV and VDV1. (**C**) Neighbor-joining phylogenetic tree of the full genome VDV1 and DWV consensus sequences. Bootstrap values for 1000 replicates are shown for the groups with more than 50% bootstrap support.

Parasites and pathogens are strongly implicated in honey bee colony losses, impacting pollination services^4,9^. Pathogens shared with honeybees could affect wild insect pollinators^10,11^ thereby posing additional risks to the services provided by wild pollinators^12^. In Europe and the US, elevated losses of honeybee colonies are associated with the mite *Varroa destructor* ^13^. This parasite feeds on the internal tissues of both pupal and adult honey bees, and in the process can vector a number of RNA viruses^13,14^. These include Deformed wing virus (DWV)^15^, also referred to as DWV-A^16^, and DWV-like viruses such as the Varroa destructor virus 1 (VDV1)^17^, (Fig. 1A), also referred to as DWV-B^16^. In this paper we will use VDV1 to be consistent with the current nomenclature from the International Committee for the Taxonomy of Viruses (ICTV)^18,19^. DWV and VDV1 are not only the most prevalent viruses in honey bees, but are also likely to be the most significant in terms of their impact on honey bee colony health^13,20–22^.

Varroa-mediated transmission of DWV selects for highly virulent strains and decreases overall virus population diversity^23–25^. Both VDV1 and VDV1-DWV recombinants outcompete DWV and are more virulent than DWV (DWV-A) strains^24,26^, although the mechanisms of the their increased virulence remain unknown. On its own, VDV1 was identified as a major predictor of elevated winter losses in Germany^27^.

Little is known about the prevalence and range of VDV1 in the US, a serious knowledge gap considering the potential role of this virus in observed high colony loss rates. In this study, we report that VDV1 is currently widespread in the US, alongside DWV. There are at least two distinct sequence variants of VDV1 present in the US, one of which is closely related to the European VDV1 strains. The prevalence of VDV1 in tested colonies (65%) was significantly lower than that of DWV (89%). Nevertheless, average VDV1 loads in virus-positive colonies were higher than those of DWV. Although the major VDV1 variants in the samples tested by NGS were full-length, we demonstrated that in addition to the full-length VDV1 and DWV, US honey bees harbor VDV1-DWV recombinants, similar to those found in Europe with respect to genome organization, but showing sequence-level traits from the VDV1 and DWV parental virus strains typical of the US. The widespread nature of VDV1 in the USA is worrisome. When planning antiviral treatments, including RNAi-based methodologies, consideration of the sequence differences between VDV1 and DWV will be important to ensure their efficacy.

## Results

### Identification and phylogenetic analysis of the US VDV1 isolates

Analysis of NGS libraries from individual honey bee pupae sourced in Maryland and Florida in 2015–2017 revealed the presence of VDV1 alongside DWV in 4 libraries from individual bees (Supplementary Table S1). The reads were aligned to the reference DWV and VDV1 sequences to reveal complete coverage for both viruses, suggesting that full-length VDV1 and DWV constituted the majority of the DWV-like populations in the sampled pupae (Fig. 1B). VDV1 and DWV were present in pupae with a range of virus levels, accounting for 2% to 88% of the total NGS reads in three out of four libraries. DWV was present at higher levels than VDV1 in three individual pupae out of four analyzed by NGS (Supplementary Table S1; Supplementary Fig. S1B-D). We assembled consensus DWV and VDV1 sequences for each of the NGS libraries (Supplementary Data S1). The presence of VDV1 was further confirmed by sequencing the RT-PCR fragment corresponding to a section of the structural protein block, central and polymerase regions (Supplementary Data S2). DWV sequences present in all libraries matched most closely to the PA strain of DWV, identified in the US in 2006^15^ (Fig. 1C). The Florida VDV1 sequences clustered with all three previously published European VDV1 sequences, while the VDV1 strain present in the Maryland samples showed 1.3% nucleotide and 0.5% amino acid differences of the viral polyproteins when compared to the Florida VDV1 (Fig. 1C, Supplementary Data S1), suggesting it is a different strain.

There was a striking difference in the degree of genetic variability within VDV1 and DWV populations in the NGS libraries. The Shannon’s diversity index for nucleotide sequences was far higher for the DWV reads compared to those of VDV1 (Fig. 1B; Supplementary Fig. S1).

### Recent expansion of VDV1 in the US

**Figure 2:**
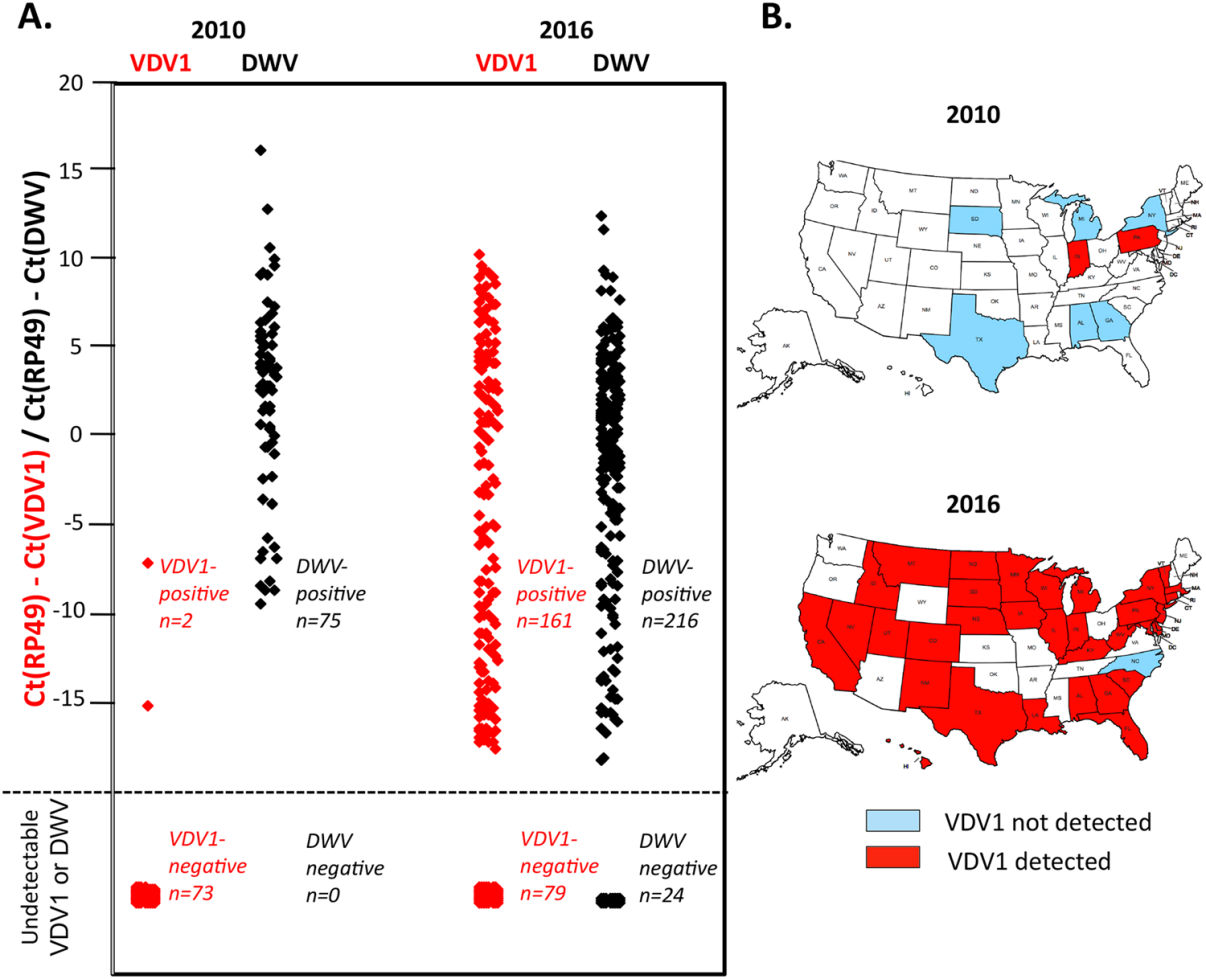
Prevalence of VDV1 and DWV in US honey bees. (**A**) VDV1 and DWV genomic RNA levels normalised to the honey bee PR49 mRNA in 2010 and 2016 honey bee colony samples. (**B**) Geographical distribution of VDV1-positive apiaries sampled in 2010 (75 apiaries from Fig. 2A) and 2016 (603 apiaries, analysed further in Fig. 3). The maps were prepared using the free online tool https://createaclickablemap.com. This figure is not covered by the CC BY licence. [Copyright © 2012 Flynndustries, LLC]. All rights reserved.

The lack of previously published nation-wide reports on US presence of VDV1 suggested a recent arrival and spread of this virus. To test this possibility, we quantified VDV1 and DWV by qRT-PCR in honey bee samples, each containing 50 pooled adult honey bees from a composite sample of 8 colonies per apiary, collected as part of the National Honey Bee Disease Survey^21^ in 2016 (n = 240 apiaries) and in 2010 (n = 75). VDV1 and DWV qPCR primers targeting the structural gene block and the polymerase regions, respectively, were designed so as to not to cross-amplify, i.e. VDV1 primers did not detect DWV and vice versa (Supplementary Table S2). To exclude possible effects of prolonged storage on the RNA quality and to allow comparison between samples which were stored at −80 °C for several months or for seven years, we normalized the viral genomic RNA Ct values to that of the honey bee RP49 mRNA, which was done by subtracting the virus Ct value for DWV or VDV1 genomic RNAs from the Ct for the honeybee RP49 mRNA for each RNA extract to obtain delta Ct values (Fig. 2A). We found a significant increase in VDV1 estimated true prevalence, from 0% of sampled apiaries in 2010 (95% confidence interval (CI) 0% to 4.3%) to 66.1% in 2016 (95% CI 61.7% to 70.3%) (Fig. 2A) (p < 0.01: Chi-square statistics). There was also an increase in the geographic range of VDV1, which was found in the majority of the sampled states in 2016 (Fig. 2B). Notably, the estimated true prevalence of DWV in US apiaries was 100% in 2010 (95% CI 100% to 100%) and 93.8% in 2016 (95% CI 90.8% to 96.3%), Fig. 2B. Detection of high levels of DWV in the 2010 samples further confirmed that the low incidence of VDV1 detection was not a result of viral RNA degradation and VDV1 was indeed much less common in 2010 compared to 2016.

### Analysis of factors affecting VDV1 incidence and levels

**Figure 3:**
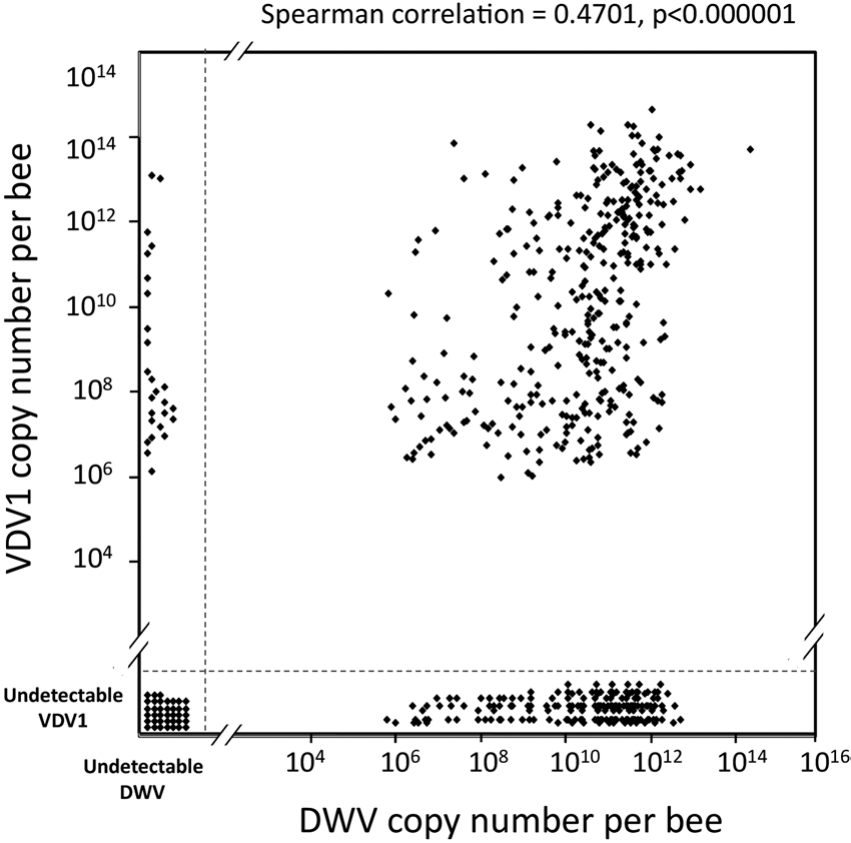
The prevalence of VDV1 and DWV in 603 US honey bee samples from 2016. Positive correlation between DWV and VDV1 (Spearman correlation = 0.4701, n = 363, p < 0.000001).

In total, VDV1 was quantified in 603 apiary samples collected from May-November 2016 in apiaries across the US. A large proportion of the samples (n = 389, 64.5%), distributed throughout the US (Fig. 2B), contained VDV1 above the detection threshold (Fig. 3). The same samples were also tested for the presence of the other honeybee pathogens as part of the National Honey Bee Disease Survey^21^. This pathogen information, together with metadata from the survey samples, allowed for an analysis of factors that might influence VDV1 incidence and levels in the honey bees.

Overall VDV1-positive apiaries had more colonies (188 ± 45.7) than did VDV1-free apiaries (40 ± 6.3; Student t test, p = 0.017). We observed an association between VDV1 levels and Varroa load in VDV1 prevalent colonies (R^2^ = 0.025, F = 6.53, 387, 1, p = 0.0127). Neither Nosema prevalence nor Nosema spore load were associated with VDV1. We correlated the levels of VDV1 and other honey bee viruses quantified from the same samples during the National Honey Bee Disease Survey^22^, including DWV, Kashmir bee virus (KBV), Acute bee paralysis virus (ABPV), Israel acute paralysis virus (IAPV), Chronic bee paralysis virus (CBPV), and Lake Sinai virus 2 (LSV2). Although VDV1 was detected in a lower proportion of apiaries than DWV (n = 539, 89.4%), the average number of genome copies of VDV1 in VDV1-positive apiaries, (7.45*10^12^ ± 1.62*10^12^) was the highest for all viruses quantified in this study, including DWV in DWV-positive apiaries (1.04*10^12^ ± 0.53*10^12^). Notably, the average genome copy numbers of other tested RNA viruses in their respective positive samples were more than 100 times lower than that of VDV1, ranging from 1.12*10^9^ ± 0.84*10^9^ for KBV to 5.31*10^10^ ± 3.27*10^10^ for IAPV (average number of virus genome copies per bee ± standard error), (Supplementary Fig. S2).

The analysis of pathogen load in the samples showed significant correlation only between VDV1 and DWV. In dually infected colonies, the total loads of DWV and VDV1 were positively correlated (Spearman correlation coefficient rs = 0.4701, n = 363, P < 0.000001) (Fig. 3). Notably, the distribution of the two virus levels was not random; the majority of samples with high VDV1 levels also had high DWV levels. Indeed, we observed a statistically significant under-representation of samples that had detectable VDV1 and undetectable DWV (contingency table analysis, P < 0.001; Supplementary Fig. S3).

### Identification of VDV1-DWV recombinants

**Figure 4:**
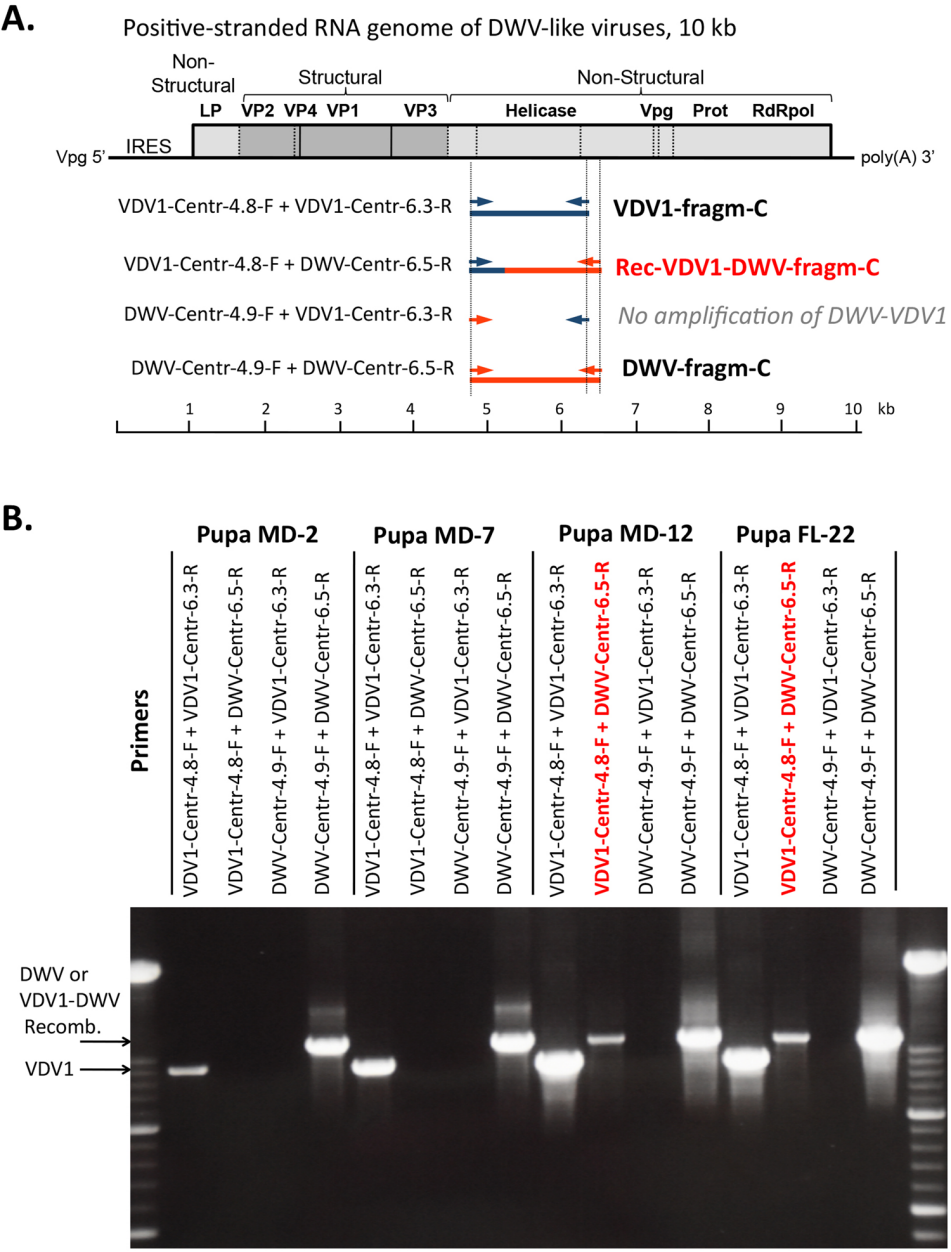
Detection of the VDV1(CP)-DWV(NS) recombinant sequences in US honey bee samples by RT-PCR. (**A**) Positions of the VDV1- and DWV-specific primers and RT-PCR products in the viral genomic RNA. (B) Amplification of the central 1.4 kb genomic region by RT-PCR using the four possible combinations of the DWV- and VDV1-specific primers flanking the main central recombination region. The reactions with detected recombinants are shown in red.

All VDV1-DWV recombinants reported to date contain recombination points located in the central, helicase-coding region of the genome^24,25,28,29^. To determine if these sequences were present in bees dually infected with DWV and VDV1, we used the four possible combinations of strain-specific primers flanking the expected recombination region between the capsid protein (CP) genes and the non-structural (NS) genes, positions 4.9–6.5 kb of the VDV1 and DWV genomes, to amplify VDV1, DWV and possible recombinants in the RNA samples used for NGS by RT-PCR as described previously^30^ (Fig. 4A, Supplementary Table S2). In all four sampled honey bee pupae we observed amplification of the DWV and VDV1 sequences, and in two of them (Pupa-MD-12 and Pupa-FL-22) clear bands were detected corresponding to potential VDV1(CP)-DWV(NS) fragments (Fig. 4B), the recombinant nature of which was confirmed by sequencing (Supplementary Data S3). Notably, no amplification of DWV(CP)-VDV1(NS) recombinants was observed (Fig. 4B), in agreement with previous reports that only recombinants of VDV1(CP)-DWV(NS) were present^24,25^. The proportions of these recombinant variants were much lower than those of the full-length DWV and VDV1, as evidenced by the NGS analysis (Fig. 1B; Supplementary Fig. S1) and the lower intensity of the gel band (Fig. 4B). NGS libraries from insects infected with VDV1 were screened for the presence of split NGS reads (reads which could be split in two parts, aligning to different references) and discordant NGS read pairs (read pairs in which each read aligns to a different reference) the presence of which indicates recombination events. We identified such reads only in the libraries in which recombinants in the central region were also identified by RT-PCR (Pupa-MD-12 and Pupa-FL-22; Fig. 4B). The positions of all potential recombination events detected in the “Pupa-MD-12” NGS library based on read mapping are shown in the Supplementary Fig. S4A. These recombination sites were identified in the LP region (positions 1–2 kb) as well as the main NS region (positions 5 to 10 kb), where mainly switches from VDV1 to DWV took place. Additionally, alignment of the libraries to the VDV1(CP)-DWV(NS) sequenced fragment allowed for the detection of reads arising from recombinants (Supplementary Fig. S4B).

**Figure 5:**
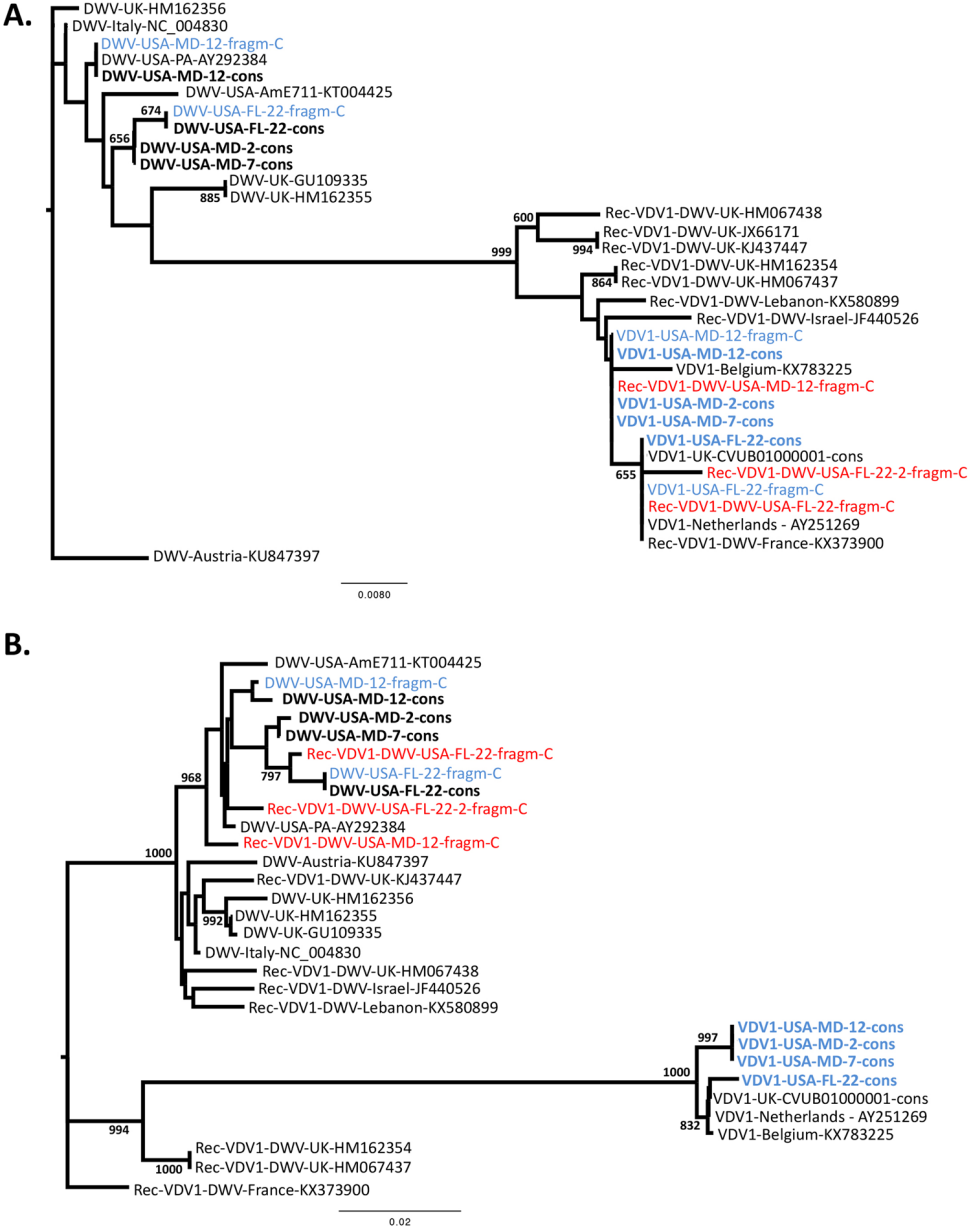
Phylogeny of the US VDV1-DWV recombinants. Neighbor-joining phylogenetic trees of (**A**) the VDV-derived 272 nt parts (corresponding to positions 4955–5226 of VDV1, GenBank accession AY251269) and (**B**) the DWV-derived 931 nt parts (corresponding to positions 5523–6453 of DWV, GenBank accession AY292384). Bootstrap values for 1000 replicates are shown for the groups with more than 50% bootstrap support. Recombinant US sequences have prefix “Rec-VDV1-DWV-USA-”.

To further investigate the origins of these recombinants we aligned them to the GenBank database, which allowed unambiguous identification of the VDV1- and DWV-derived portions of the amplified recombinant fragments (Fig. 4). We then carried out a phylogenetic analysis by aligning these VDV1- and DWV-type fragments separately with the full-length DWV and VDV1 sequences, as well as the VDV1 and DWV derived portions of the European and Middle Eastern recombinants, and generating a neighbor-joining phylogenetic tree (Fig. 5). In the case of the 272 nt VDV1 parts, in some cases, we observed complete identity with the VDV-1 consensus and the VDV1 RT-PCR sequences derived from the same bee (Fig. 5A). Since this region of VDV1 was not particularly divergent, analysis of the longer 931 nt DWV-derived parts was more informative. There was no complete sequence identity between the DWV portions of the US recombinants and the full-length DWV consensus sequence from their respective honey bees, likely due to the high level of DWV nucleotide diversity. Despite this, all DWV portions of the US recombinants clustered together with the US DWV-PA and the DWV consensus sequences from the source bees (Fig. 5B). Results of phylogenetic analyses of the VDV1-DWV recombinant sequences strongly suggest that these were generated as a result of a recent event involving the US strains of DWV and VDV1, rather than being imported from other regions. Although the proportion of reads around the breakpoint for the recombinants were below those of the parental strains in the analyzed samples (Fig. 4B), the accumulation above RT-PCR detection levels suggests their replication and viability. Considering that in several UK regions the VDV1(CP)-DWV(NS) type recombinants constitute the majority of DWV-like viruses and represent the major virulent variants in the Varroa-infested colonies in the UK^24^, the presence of US VDV1-DWV recombinant strains should be closely monitored in addition to the full-length VDV1 and DWV variants.

## Discussion

In this study we provide the first evidence that VDV1^17^ is widespread in the United States. This is a matter of concern because VDV1 was shown to have higher pathogenicity in honey bees compared to DWV^26,27^. Using next-generation sequencing (NGS), we demonstrated that full-length VDV1 sequences were present alongside DWV (DWV-A type; Fig. 1, Supplementary Data Fig. S1) in honey bee pupae collected in 2015 and 2016. We observed two VDV1 variants, one first identified in Florida that belongs to the same clade as European VDV1 (VDV1-USA-FL-22) and a distinct strain sourced in Maryland in 2015 (VDV-1-US MD-2, −7, −12; Fig. 1C).

By sampling over 600 colonies with a highly specific qRT-PCR test for VDV1 we found that VDV1 was widespread in the US in 2016 with presence in about 65% of the tested colonies (Figs 2A and 3). It was detected in 33 out of 34 tested states including Hawaii (Fig. 2B). In fact, the only sampled state in which we did not detect VDV1 was North Carolina, from which only a single apiary was screened (Fig. 2B). In sharp contrast, the proportion of VDV1-infected colonies in 2010 was about 3% (2 samples out of 75 tested, Fig. 2A) suggesting a recent and rapid spread of VDV1 in the USA. Interestingly, quantification of DWV in the same honey bee samples showed that DWV was widespread in both 2010 and 2016, with 100% and 89% infection rates, respectively. It is likely, that the more recent expansion of VDV1 (between 2010–2016) compared to DWV, which was widespread in the US in both 2006^15^ and 2010, could explain much lower genetic diversity of VDV1 when compared to the DWV present in the same honey bees as revealed by NGS analysis (Fig. 1B, Supplementary Fig. S1, Shannon’s diversity). However, other bottleneck effects might also contribute to lower diversity to VDV1. Given these data, it seems likely that very few genotypes of VDV1 were introduced to US honey bees. Further testing of VDV1 variants in the US and worldwide will help establish possible points of introduction for this virus and timing by which at least two VDV1 variants arrived in the US (Fig. 2B).

We also found that levels of VDV1 in infected colonies were higher than those in DWV-infected colonies. Although pathogenicity of the US variants of VDV1 and DWV are unknown and will require additional studies, the higher levels of VDV1 in the infected colonies compared to that of the DWV-positive colonies may indicate higher virulence of the US VDV1 strains. If true, this observation would be in agreement with reported higher virulence of VDV1 compared to DWV in Europe^26,27^.

In this study a weak association was found between the VDV1 load and Varroa load in VDV1-infected colonies. Given a clearly demonstrated connection between Varroa mite infestation and the incidence and pathogenicity of all DWV-like viruses^23,31,32^, it seems quite likely that this virus is vectored by these parasitic mites. This is also consistent with the observation that VDV1 is more likely to be present in apiaries with a larger number of colonies. Higher density of hosts (in this case honeybee colonies) could increase chances of the mite-mediated spread of VDV1 which has higher virulence compared to DWV^26,27^, perfectly fitting the model that increases in host density lead to the dominance of more virulent strains of pathogens^33^. On the other hand, long-term surveys by the Bee Informed Partnership (www.beeinformed.org) did not find higher disease incidents in colonies from large versus small apiaries. Additional monitoring of VDV1 and Varroa load over time will be necessary to determine whether this trend is consistent and important to virus transmission.

We observed both a positive correlation between DWV and VDV1 loads in dually-infected colonies (Fig. 3) as well as an under-representation of colonies that were VDV1-positive, but DWV-negative (Supplementary Fig. S2). There are several non-exclusive possibilities to explain why high levels of VDV1 would be more likely to occur alongside high levels of DWV. First, replication of DWV to high levels may be a prerequisite for VDV1 to achieve high levels. While the samples shown in Fig. 2 are pooled, analysis of NGS libraries (Fig. 1B, Supplementary Fig. 1S) indicates that individual bees could be infected with a mixture of both viruses. Second, the replication of VDV1 to high levels may require specific conditions (i.e., Varroa infestation or a particular colony health status), which always result in high DWV levels when present.

Although analysis of the NGS data clearly showed that full-length VDV1 sequences were present alongside the full-length DWV, we also found strong evidence for VDV1-DWV recombinants, both by direct sequencing of RT-PCR products (Figs 4 and 5) and bioinformatic analysis of NGS libraries (Supplementary Data Fig. S4). NGS analysis revealed a number of potential recombination breakpoints throughout the viral genome, except the structural protein coding region, with the highest proportion of the recombinant reads and/or discordant NGS pairs (reaching 0.2–0.3% of total NGS coverage) reported in the IRES region (Supplementary Data Fig. S4). Recombination points in this region were also reported in the genomes of VDV1-DWV recombinants in the UK^25,28^. We were particularly interested in analyzing recombination points in the central genomic region because these VDV1-DWV recombinants were highly virulent in the UK^24^. By using RT-PCR we detected and sequenced recombinants containing VDV1-derived 5′ proximal structural (CP) and DWV-derived non-structural (NS) 3′ proximal gene blocks which were similar to those found in the UK^24,28^, Israel^29^, and France^25^. Moreover, we demonstrated that these recombinants derived from the US VDV1 and DWV parent viruses (Fig. 5). It is possible that the VDV1(CP)-DWV(NS) recombinants are widespread and their the presence contributed to the positive correlation observed between the VDV1 and DWV levels (Fig. 3). In this study we quantified VDV1 and DWV using qPCR primers targeting the CP and the NS regions, respectively, therefore such samples with the VDV1(CP)-DWV(NS) recombinants may appear in the Fig. 3 scatter plot as having nearly equal levels of DWV and VDV1 (Fig. 3). In future studies, additional tests will be necessary to determine the precise composition of virus complexes containing DWV, VDV1 and their recombinants in individual honey bees rather that pooled samples as in this study. Such VDV1-DWV recombinants pose an additional threat to US honey bees because recombinant viruses of the same type, but generated from different parental DWV (DWV-A) and VDV1 (DWV-B) strains, are most virulent in the UK^24^. Generation of VDV1-DWV recombinants suggests that the VDV(CP) × DWV(NS) combination may have selective advantages over the parental viruses. There is an urgent need to monitor VDV1 and recombinants to determine sources and possible control strategies for these viruses. Thanks to widespread samples available from the National Honey Disease Health Survey^22^, it should be possible to determine the source, dispersal, and arguably the bee population consequences of VDV1 during the past seven years. Along with reducing the risk of further introductions, knowledge of VDV1 and the rest of the DWV-like viruses can aid novel antiviral therapies including RNAi-based strategies.

## Methods

### Honey bee sampling, virus quantification, and detection

Honey bee samples were collected as a part of the APHIS honey bee survey^22^. Quantification of virus RNA and the honeybee pRP49 transcript were performed using the qRT-PCR SYBR-green kit (Eurogentec) as described previously^21,22^. The VDV1 specific primers were designed to exclude cross detection of DWV (Table S2). cDNA amplification of the cDNA fragments of DWV, VDV1 and their recombinants, which were used for sequencing, was carried out by RT-PCR using DWV- and VDV1-specific oligonucleotide primers (Table S2) as described previously^24,28^. Quantification of DWV and the honey bee mRNA internal controls were carried out as described previously^22^. True prevalence with 95% confidence intervals of DWV and VDV1 in apiary tests was estimated using the epi.prev function of the epiR package (v0.9-87; R v3.3.3)^34^, based on the Blaker method with a conservative 95% sensitivity and specificity. For NGS analysis and amplification of the RT-PCR fragments for sequencing, total RNA was extracted from individual honey bee pupae collected in Maryland and Florida in 2015 and 2017, respectively, as in previous studies^24,28^.

### RNAseq data analysis

Pre-analysis processing of the raw Illumina data (paired-end 150 nt reads) was performed in three steps with BBDuk (version 36.92), a tool in the BBTools package^35^. First, extra bases and adapters were trimmed using the file of Illumina Truseq and Nextera adapters sequences included with the BBTools package (parameters: ref = adapters.fa ftm = 5 ktrim = r k = 23 mink = 11 hdist = 1 tpe tbo). Next, PhiX contaminant sequences were removed using a Kmer filtering approach and the PhiX reference sequence included with the BBTools package (parameters: ref = phix174_ill.ref.fa.gz k = 31 hdist = 1). Finally, data was quality trimmed on both ends of the read to Q6 using the Phred algorithm and length filtered such that only those reads with a minimum length of 75 bp after all pre-analysis processing steps were retained (parameters: qtrim = rl trimq = 6 minlength = 75).

Cleaned data from each sample library were individually aligned to reference sequences of DWV^15^ (GenBank Accession GU109335), VDV1^17^ (GenBank accession AY251269) and DWV-C^16^ or the recombinant RT-PCR product (for Supplementary Fig. 4B) using Bowtie2^36^ (version 2.2.9; parameters: -q–local–very-sensitive-local). Counts, coverage and Shannon’s diversity index estimates for each nucleotide position were calculated from SAMtools mpileup^37^ (version 1.2) output. In order to correct Illumina sequencing chemistry errors, the Shannon diversity index estimate was error corrected^38^. Briefly, the error model was calibrated using data from an Illumina library from a bee injected with a cloned virus of known sequence. Data from positions 5230 to 6600 of the sequence, representing a low diversity region of the virus, were used to calculate an alphahat of 9.87546E-05 after excluding any position with a Shannon diversity index above 0.004 due to the presence of background virus diversity.

For detection of recombination breakpoints, cleaned data from each sample NGS library were individually aligned to the VDV1 and DWV references using SpeedSeq^39^ (version 0.1.2). Structural variants were detected using the lumpy smooth script from the LUMPY package^40^ (version 0.2.13) and additional genotype and coverage metrics for each variant were calculated using SVTyper, a script within the SpeedSeq package. Breakpoint positions representing recombinations between two different viral sequences were considered for further analysis. Recombination points with evidence from more the 10 supporting events based on split or discordant reads, which did not resulted in inversions, deletions or insertions, were considered.

### Phylogenetic analysis

The viral nucleotide sequences were aligned using CLUSTAL^41^, and the neighbour-joining trees were produced and bootstrapped using the PHYLIP package^42^. Bootstrap values were obtained from 1000 replications.

## Electronic supplementary material

Supplementary Information
