# Supplementary material for: Recent spread of Varroa destructor virus-1, a honey bee pathogen, in the United States
Source: Sci Rep. 2017 Dec 12;7:17447. doi: 10.1038/s41598-017-17802-3 (PMC5727227; doi:10.1038/s41598-017-17802-3)
Supplement: Supplementary file 1 — Supplementary Information [file 41598_2017_17802_MOESM1_ESM.pdf]

Supplementary Information

Recent spread of Varroa destructor virus-1, a honey bee pathogen, in the United States.

Authors:

Eugene V. Ryabov[1*], Anna K. Childers[1], Yanping Chen[1], Shayne Madella[1], Ashrafun Nessa[2], Dennis vanEngelsdorp[2], Jay D. Evans[1*]

Affiliations:
[1]US Department of Agriculture, Agricultural Research Service, Bee Research Lab, Beltsville, MD, USA
[2]University of Maryland, Department of Entomology, College Park, MD, USA

This PDF file includes:

Supplementary information

Supplementary Table 1. Summary of the NGS libraries for the honey bee pupae infected with VDV1.

Supplementary Table 2. Primers used in this study.

Supplementary Figure S1. DWV-like NGS read coverage and Shannon's diversity index for VDV1 positive NGS libraries.

Supplementary Figure S2. Average load and prevalence of honeybee viruses.

Supplementary Figure S3. Mutual influence of DWV and VDV1, contingency table analysis.

Supplementary Figure S4. Detection of VDV1-DWV recombination products in the NGS library from a single pupa (Pupa-MD-12).

Supplementary Data S1. Full-length VDV1-USA and DWV consensus sequences generated using NGS data.

Supplementary Data S2. DWV, VDV1 and VDV-1-DWV recombinant sequences generated by RT-PCR.

Supplementary Table 1. Summary of the NGS libraries for the honey bee pupae infected with VDV1. Shown are the total number of reads in the library, DWV-type reads, VDV1-type reads, their

proportions and combined DWV and VDV1 coverage normalised to the coverage of the honeybee RPS5 and actin mRNAs.

NGS library	Sample description	Total reads	Total DWV reads (aligned to GenBank Acesion number GU109335)	Total VDV1 reads (aligned to GenBank Accession number AY251269)	DWV reads (%)	VDV1 reads (%)	DWV and VDV1 to RPS5 mRNA (GB45730) coverage ratio	DWV and VDV1 to actin mRNA (B44311) coverage ratio
Pupa_No_2_MD	Individual pupa, Maryland, October 2015	14918458	845852	65458	5.67%	0.44%	5.48	8.15
Pupa_No_7_MD	Individual pupa, Maryland, October 2015	12314066	227910	24312	1.85%	0.20%	1.70	2.45
Pupa_No_12_MD	Individual pupa, Maryland, October 2015	15250702	4955503	8543796	32.49%	56.02%	1232.40	1532.29
Pupa_No_22_FL	Individual pupa, Florida, January 2017	20781666	5519537	2697953	26.56%	12.98%	185.69	97.45

Supplementary Table 2. Primers used in this study.

Primer ID	Nucleotide sequence (5' to 3')	Target (postions in the nucleotide sequence), polarity, GenBank accession number	Applications
**VDV1-CP-1650-For**	CGGAAACAAGTTCGGCTTC	VDV1 (1646 -1664), Forward, AY251269	RT-PCR, VDV1 fragment CP
**VDV1-CP-2850-Rev**	CTCAATTTTCTGTTCAACG	VDV1 (2836 - 2854), Reverse, AY251269	RT-PCR, VDV1 fragment CP
**VDV1-Centr-4.8-F**	CTGTAGTTAAGCGGTTATTAGAA	VDV1 (4890 - 4912), Forward, AY251269	RT-PCR, VDV1 and VDV1-DWV recombinants fragments C
**VDV1-Centr-6.3-R**	CTGAAGTACTAATCTCTGAG	VDV1 (6298 - 6317), Reverse, AY251269	RT-PCR, VDV1 fragment C
**DWV-Centr-4.9-F**	ACTGTAGTCAAGCGGTTACTTGAG	DWV (4911 - 4934), Forward, AY292384	RT-PCR, DWV fragment C
**DWV-Centr-6.5-R**	CTTGGAGCTTGAGGCTCTGCA	DWV (6521 - 6541), Reverse, AY292384	RT-PCR, DWV and VDV1-DWV recombinants fragments C
**VDV1-POL-8910-For**	GCGTCGTACCGTGCCGCTA	VDV1 (8912 - 8930), Forward, AY251269	RT-PCR, VDV1 fragment POL
**VDV1-POL-10060-Rev**	GACGCATTGCCGTTATGC	VDV1 (10065 - 10082), Reverse, AY251269	RT-PCR, VDV1 fragment POL
**qVDV1-CP-F**	CTGTAGTTAAGCGGTTATTAGAA	VDV1 (4890 - 4912), Forward, AY251269	qRT-PCR-VDV1
**qVDV1-CP-R**	GGTGCTTCTGGAATAGCGGAA	VDV1 (4966 - 4986), Reverse, AY251269	qRT-PCR-VDV1
**VDV1-CP-CONTROL**	CTGTAGTTAAGCGGTTATTAGAAAAATATCACTT GGCGACGCAACCCCAGGAATCCGCCAATTCAGG TACGGTTATTTCCGCTATTCCAGAAGCACC	VDV1 (4890 - 4986), Forward, AY251269	VDV1-CP Standard
**DWV-F8668**	TTCATTAAAGCCACCTGGAACATC	DWV (8645 - 8668), Forward, AY292384	qRT-PCR-DWV
**DWV-B8757**	TTTCCTCATTAACTGTGTCGTTGA	DWV (8780 - 8757) Reverse, AY292384	qRT-PCR-DWV
**ABPV-F6548**	TCATACCTGCCGATCAAG	ABPV (6531 - 6548), Forward, AF150629	qRT-PCR-ABPV
**KIABPV-B6707**	CTGAATAATACTGTGCGTATC	ABPV (6727 - 6707), Reverse, AF150629<br>IAPV, (6812 - 6792), Reverse, KY243933	qRT-PCR-ABPV<br>qRT-PCR-IAPV
**IAPV-F6627**	CCATGCCTGGCGATTCAC	IAPV, (6610 - 6627), Forward, KY243933	qRT-PCR-IAPV
**CBPV1-qF1818**	CAACCTGCCTCAACACAG	CBPV, (1790 - 1807), Forward, KY937971	qRT-PCR-CBPV
**CBPV1-qB2077**	AATCTGGCAAGGTTGACTGG	CBPV, (2085 - 2066), Reverse, KY937971	qRT-PCR-CBPV
**KBV.F**	TGAACGTCGACCTATTGAAAAA	KBV, (5408 - 5429), Forward, AY275710	qRT-PCR-KBV
**KBV.R**	TCGATTTTCCATCAAATGAGC	KBV, (5534 - 5514), Reverse, AY275710	qRT-PCR-KBV
**qLSV2-F1722**	CGTGCTGAGGCCACGGTTGT	LSV2, (1776 - 1795), Forward, HQ888865	qRT-PCR-LSV2
**qLSV2-R1947**	GCGGTGTCGATCTCGCGGAC	LSV2, (2000 - 1982), Reverse, HQ888865	qRT-PCR-LSV2
**RP49-qF**	AAGTTCATTCGTCACCAGAG	Honeybee RP49 mRNA, (206 - 225), Forward, XM_016914656	qRT-PCR-RP49
**RP49-qB**	CTTCCAGTTCCTTGACATTATG	Honeybee RP49 mRNA, (410 - 389), Reverse, XM_016914656	qRT-PCR-RP49

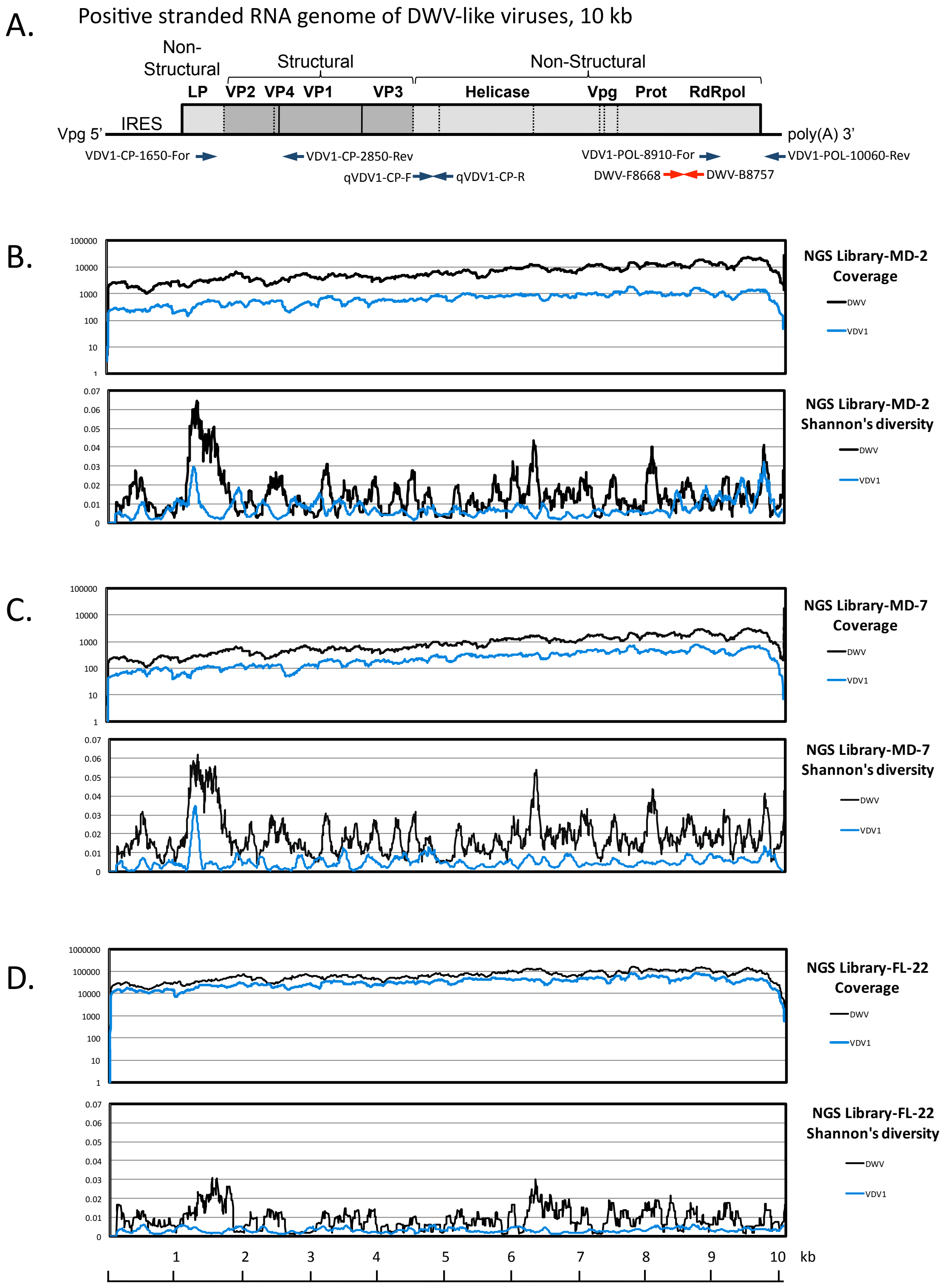

Supplementary Figure S1. DWV-like NGS read coverage and Shannon's diversity index for VDV1 positive NGS libraries. (A) Genome organization of DWV and VDV1 with positions of the primers used for RT-PCR amplification of VDV-1 and qRT-PCR quantification of DWV and VDV1. (B-D) NGS read coverage and Shannon's diversity index profiles (100 nt window average) for DWV and VDV1 in individual honey bee pupae for three NGS libraries. Data for Pupa-No-2-MD shown in Fig. 1.

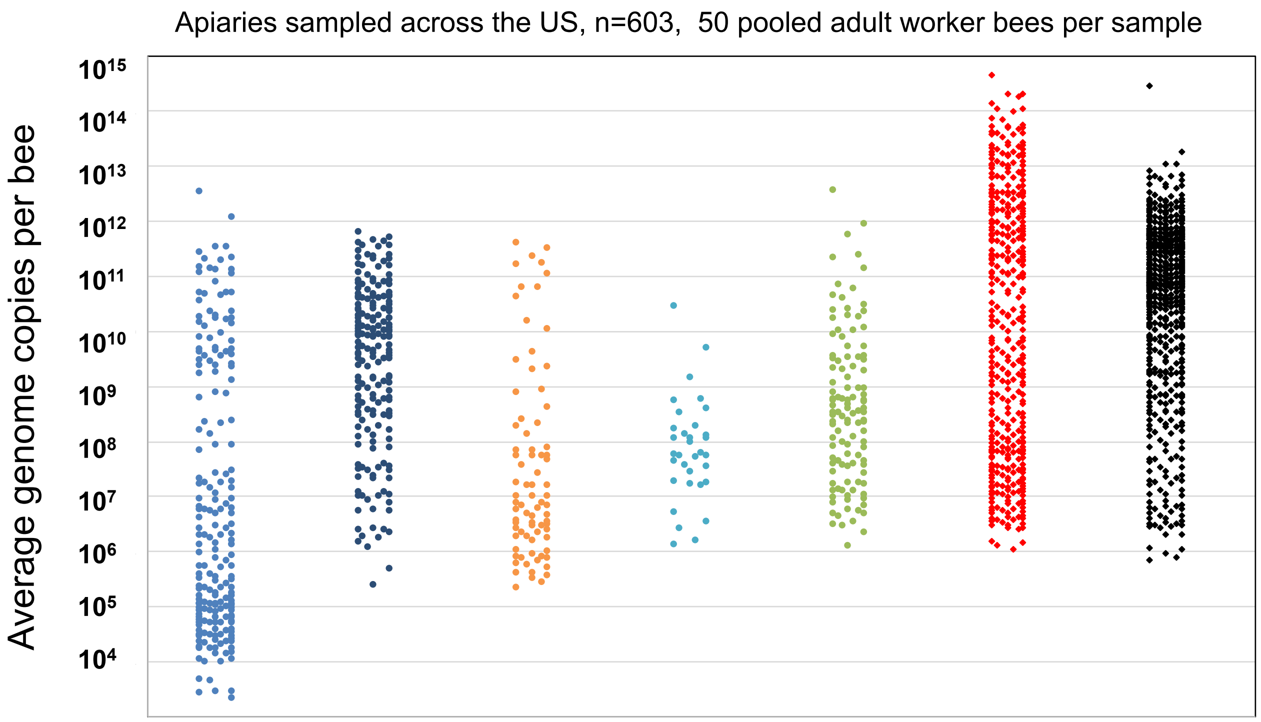

VIRUS	ABPV	LSV2	CBPV	KBV	IAPV	VDV1	DWV
Genome copies per honeybee bee Average ± SE	**3.68*10^{10}** ±1.78*10^{10}	**4.39*10^{10}** ±0.72*10^{10}	**1.89*10^{10}** ±0.75*10^{10}	**1.13*10^{9}** ±0.84*10^{9}	**5.31*10^{10}** ±3.27*10^{10}	**7.45*10^{12}** ±1.6*10^{12}	**1.04*10^{12}** ±0.5*10^{12}
Prevalence	33.6%	31.3%	13.3%	5.5%	18.9%	64.5%	89.4%

Supplementary Figure S2. Average load and prevalence of honeybee viruses.

Contingency table analysis, P<0.0001

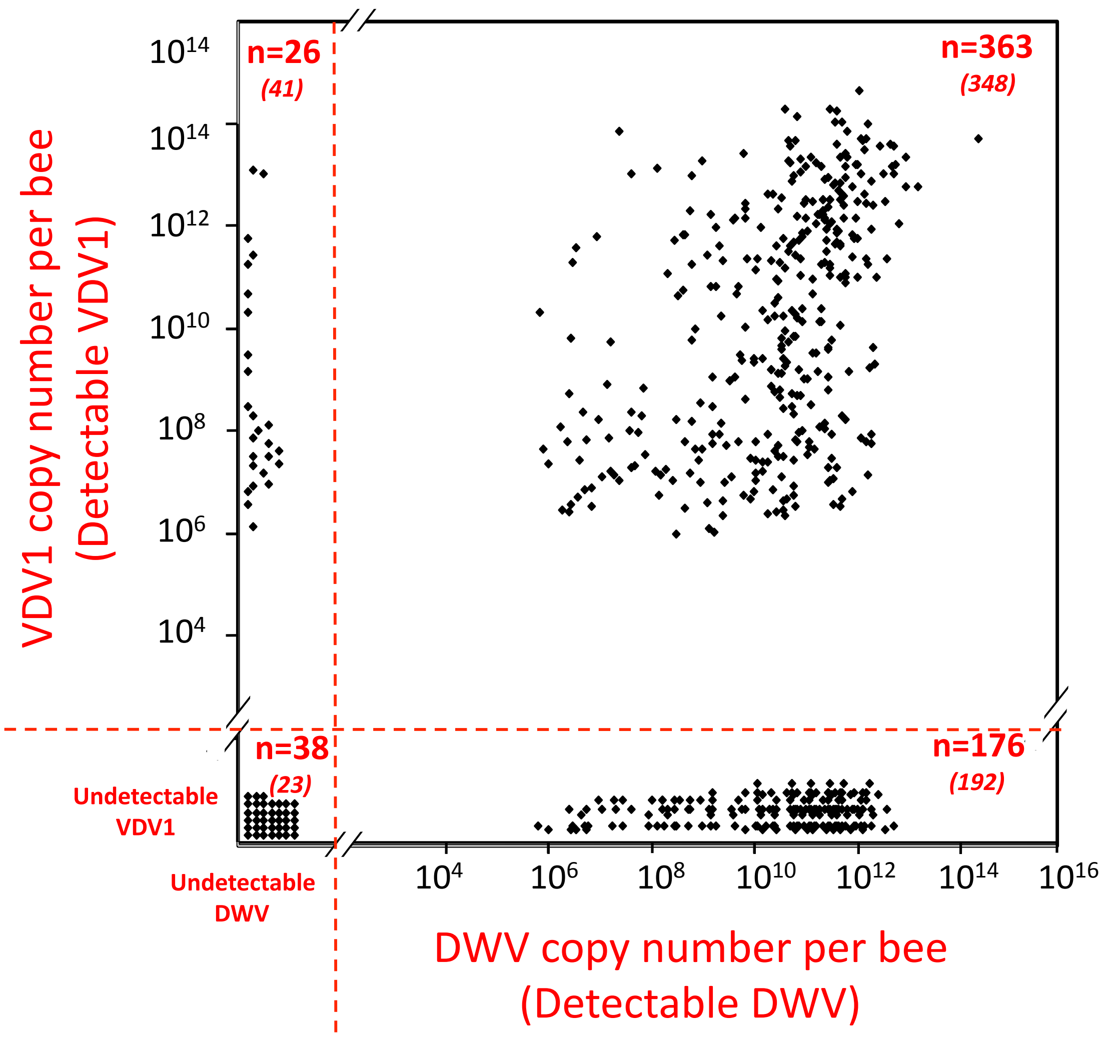

Supplementary Figure S3. Mutual influence of DWV and VDV1, contingency table analysis. The honey bee colonies with detectable VDV1 and undetectable DWV are significantly under-represented (P<0.0001). Expected frequencies are shown in italics in brackets below the observed.

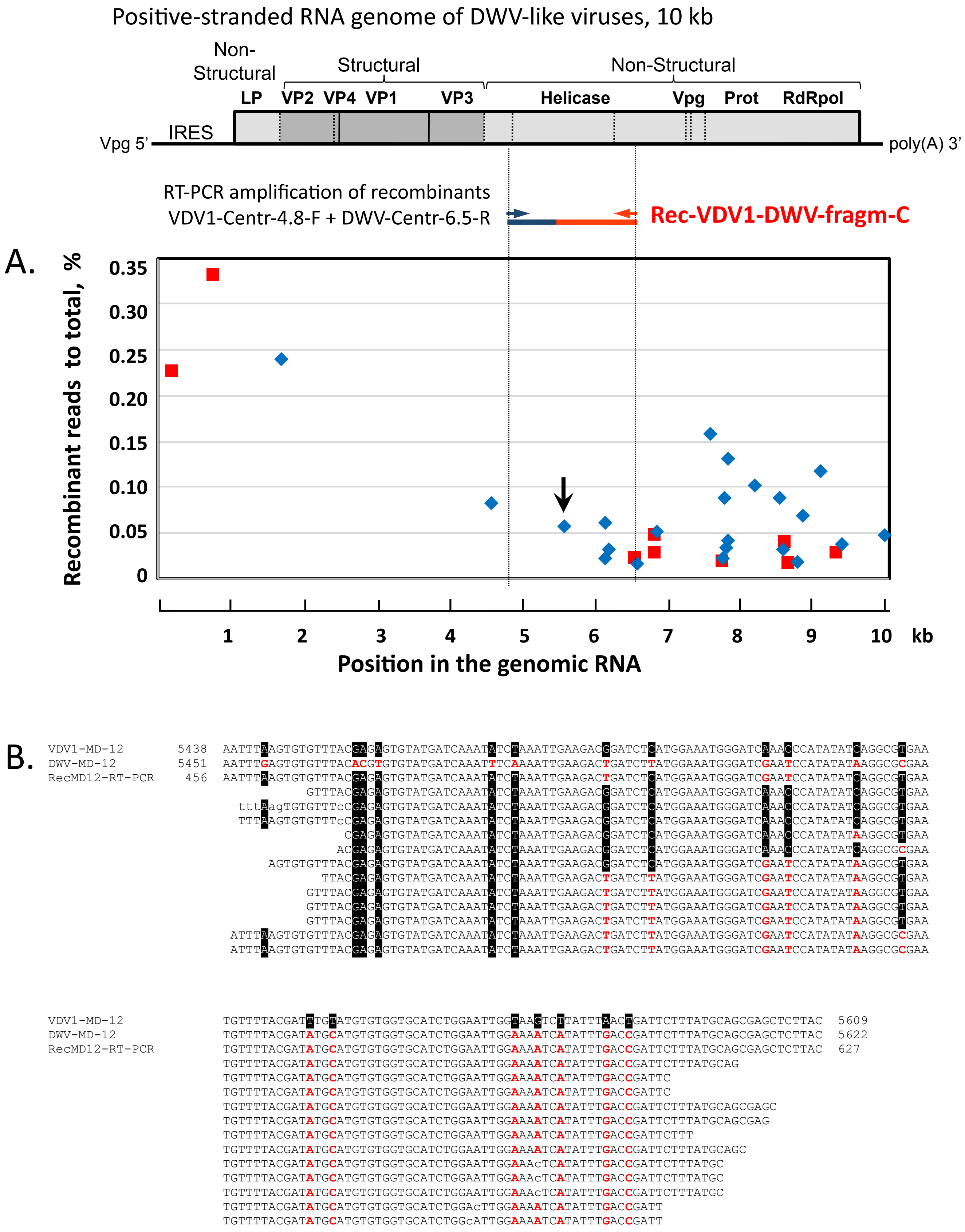

Supplementary Figure S4. Detection of VDV1-DWV recombination products in the NGS library from a single pupa (Pupa-MD-12). (A) Identification of the recombination sites using the LUMPY package. The sites are shown along the viral genome map, X-axis. The

recombination points are indicated as red squares for the DWV to VDV1 sites, and as blue diamonds for the VDV1 to DWV sites. The percentage of the NGS reads supporting recombination to total NGS depth of coverage is shown on the Y-axis. Only recombination points with more than 10 supporting events (split or discordant NGS reads) are reported. The black arrow indicates position of the recombination point possibly corresponding to that identified in the RT-PCR product amplified using VDV1 and DWV-specific primers. (B) The recombinant VDV1-DWV reads aligned to the Rec-MD-12-RT-PCR reference sequence (Supplementary Data S2, "Rec-VDV1-DWV-USA-MD-12-fragm-C") and VDV1 and DWV sequences from the pupae MD-12 (Supplementary Data S1, "VDV1-USA-MD-12-cons" and "DWV-USA-MD-12-cons"). The VDV1- and DWV-type divergent positions are highlighted with black and shown in red bold, respectively.

Supplementary Data S1. Full-length VDV1-USA and DWV consensus sequences generated using NGS data.

>VDV1-USA-MD-2-cons
GGCGATTTATGCCTTCCATAGCGAATTACGGTGCAACTAACAATTTTAGATAGTAGCCATGAACAAACATTATGAT
TACTCACTACGTATTGATCATTTTTACAATGACTTGCGTAGCATGAAGCGCATGCTTGTAGTTATAACTATGTTAT
TTTGCAAGTTGGAGATAATTGTATTGGATTATGGATGCGTGCACTAAGTGTCTACATCTATAGTCGTTTGTGGTTC
AAGTTTTTGTGTTAGTAGTACAATTTTGAAGAATGTAAGTATCGTATGAATGATATTTGAATGACAACACTGAAGT
ATAAAATATATAAAATCCAAAAATATTTTAAATTTTATTCAGTGTAGTGTTTAATAGAGTAGAATGCCATGTGACC
GCTCAAAGAAGTCCATTATGGTATATCATTCGAAGTTGAATACTTGTGTATAGTTATTGTATTTTATTAGTAATAT
TAGTAGTCCGTAACTATCATAATCCTATTATAGTTTGATTATATGATAGACCACTGCAGTATCGAGTAGAGTTTAG
AAAGAGTAGTGCAATAGTAAGATCACTGTCACCGACCACTTATTGTAATAGTGAGGTTTGTCGGAAACCAGTTATT
GTGCAGCGACTAGCAATCGTGAATCAATATAGTTGGTATTCTAAATATGAGACGATTCGGCGATTTTATTGCGACT
GAAATTTCATATTTAGCATGTCAGGTCTTATTATGAATGCTCGAGTATTTATTTCTGCGGTAGAGTAGGGACCCCT
CTATCTTTCAGGTACTGTATGAGGCGAAAGTGTGAAAGTAATTTATGTCTCTATACATAAGTGACTGTATCGGGAT
TTCCTTTGGCAAGAATCCTTTTAATACAGTATAATTTATGCTACGGTACGTTACGTTCGCAGGGCACCCGTTAATG
TCACATAGCCCAGACGATGACGAATGGAAAGACATTACTTTTTATTTTAATGCTACGATTATTGCTGTTTTATTTT
GCTGTTTTTATTTGCTATTATATTTTGCTATTTTATTATTGCTAAATATATTTCTTTGCTATTTTTGCTTTATATA
TTAGATTCAATTCTTTTTATTTTATATTTTCAATTTGATTTCGATTTTGAAGGTAAATATATATAAAAATGGCATT
TAGTTGTGGAACTCTTTCTTATGCTGCTGTTGCTCAAGCTCCCTCTGTAGCTCATGCTCCCCGTAGTTGGGAGATT
GATGAAGCTAGGCGTCGACGTGTTATTAAGCGTTTGGCGTTGGAACAGGAACGGATTCGAAACGTTCTTGATGTCA
CTGTGTATGATCATACAACGTGGGAGCAAGAGGATGCGCGTGATAATGAGTTCCTTACGGAACAATTGAATAATTT
ATATACGATATATTCTATAGCTGAAAGATGTGCCCGCCGCCCTGTTCAAGAACATGTCCCCATTTCAATCAGTAAT
AGATATTCCCCTTTAGAATCCCTTAAGATTGAGGTAGGACAAGATGCGGGTGAGTTCGTATTTAAGAGACCCAAAT
ATACAAAGATTTGTAAGAAAGTGAAACGGGTGGCATCAAAATTTGTGCGCGAGAAAGTTGTTAGGCCCGTTTGTAA
TCGATCGCCCATGTTATTATTTAAAATTAAGAAAGTAATATATGATTTACATTTGTATCGGTTACGGAAACAAGTT
CGGCTTCTCAGACGCGAAAAACAGCGTGAATATGAGTTAGAGTGTGTTACTAGTTTGCTACAGCTATCTAATCCTG
TTTCAGCTAAACCTGAGATGGACAATCCTAATCCTGGTCCAGATGGTGAAGGTGAAGTTGAATTAGAAAAAGATAG
TAATGTAGTATTAACTACACAACGTGATCCTAGTACCTCTATTCCTGCTCCAACTAGTGTGAAGTGGAGTAGGTGG
ACTAGTAATGATGTTGTGGATGATTATGCCACTATAACTTCGCGTTGGTATCAGATTGCCGAATTTGTATGGTCAA
AGGATGATCCATTTGATAAGGAATTGGCGCGCTTAATTTTACCTCGAGCTTTGTTATCTAGTATTGAGGCTAATTC
TGACGCTATTTGTGATGTACCTAATACTATTCCGTTTAAGGTACATGCATATTGGCGTGGAGATATGGAAGTTCGA
GTGCAGATTAACTCGAATAAATTCCAGGTTGGTCAATTACAGGCAACTTGGTATTATTCGGATCATGAAAATTTGA
ATATTCAGACGAAGCGAAGTGTGTATGGTTTTTCGCATATGGATCATGCTTTGATTAGTGCATCAGCGAGTAATGA
AGCAAAATTAGTGATACCTTTTAAACACGTATATCCATTCTTACCAACGCGCGTCGTTCCTGATTGGACAACTGGT
ATTCTTGATATGGGTACCTTAAATATTCGTGTAATTGCTCCATTACGTATGAGTGCGACGGGACCAACCACTTGTA
ATGTTGTAGTATTTATTAAGTTAAATAATAGTGAATTCACTGGTACTTCCTCTGGTAAGTTTTACGCGAATCAAAT
CAGGGCAAAACCTGAAATGGACCGTGTGTTAAATTTGGCAGAAGGATTATTAAATAATACTGTAGGTGGTTGTAAT
ATGGATAATCCGTCATATCAGCAATCTCCGCGTCATTTTGTTCCTACTGGTATGCATAGTTTAGCTTTAGGCACTA
ATTTAGTAGAGCCTTTGCATGCATTACGATTAGATGCATCAGGTACAACACAACATCCAGTTGGGTGTGCGCCTGA
TGAAGATATGACTGTATCTTCCATTGCATCACGATATGGTTTAATTCGCCAAGTGCAATGGAAGAAAGACCATGCG
AAAGGATCATTATTATTACAACTTGATGCTGATCCTTTCGTTGAACAGAAAATTGAGGGAACCAATCCAATTTCTT
TGTATTGGTTTGCTCCGGTTGGAGTTGTATCTAGTATGTTTATGCAATGGAGAGGTTCTTTAGAATATAGATTTGA
TATTATAGCTTCCCAATTTCATACGGGTAGGTTAATTGTAGGTTATGTTCCTGGACTGACTGCTTCTTTACAACGT
CAAATGGACTATATGAAATTGAAATCATCTAGTTATGTAGTGTTTGATTTACAGGAAAGTAATAGTTTTACGTTTG
AAGTGCCTTATGTGTCATACAGACCGTGGTGGGTGCGTAAGTATGGTGGTAATTATCTGCCATCCTCTACTGATGC
GCCTAGCACACTGTTTATGTATGTACAAGTGCCATTGATACCTATGGAAGCTGTTTCTGATACTATAGATATCAAT
GTGTATGTGCGTGGTGGCAGTTCGTTTGAGGTTTGTGTTCCAGTCCAACCTAGTTTAGGTTTGAACTGGAATACAG
ATTTTATATTACGTAATGATGAGGAGTACCGCGCAAAGAATGGATATGCACCATATTATGCTGGTGTGTGGCATAG
CTTCAATAATAGTAATTCGCTTATTTTTAGATGGGGTTCGGCTTCAGATCAAATTGCTCAATGGCCAACAATAACA
GTGCCTCGAGGAGAGTTGGCATTCTTGCGTATCCGCGATGCTAAGCAAGCTGCTGTAGGAACGCAACCTTGGCGTA
CTATGGTCGTTTGGCCTTCAGGTCATGGATATAATATTGGAATACCAACTTATAATGCTGAACGAGCAAGACAACT
TGCTCAGCATTTGTATGGTGGTGGGTCTTTGACAGATGAAAAGGCTAAGCAATTATTTGTGCCTGCTAACCAGCAA
GGACCCGGCAAAGTAAGTAATGGTAACCCCGTCTGGGAAGTAATGCGCGCGCCTCTTGCAACTCAGCAAGCGCATA
TACAAGATTTTGAATTTGTTGAAGCTGTTCCAGAAGGCGAAGAATCACGCAACACTACAGTGCTAGACACGACAAC
AACGTTACAGTCTAGCGGATTTGGTCGTGCTTTCTTTGGTGAGGCATTTAACGATCTTAAGACGTTAATGCGCCGA
TACCAATTATATGGTCAATTATTGTTATCCGTTACTACGGATAAGGATATTGATCATTGTATGTTTACCTTCCCTT

GTTTACCTCAAGGGTTAGCGTTAGATATAGGTTCGGCTGGATCTCCTCATGAAATATTTAATCGCTGCCGTGATGG
TATCATTCCATTGATAGCGTCAGGGTATCGGTTTTATCGAGGCGACTTACGGTTCAAAATTGTTTTCCCAAGTAAC
GTTAATAGCAATATTTGGGTACAACATCGACCAGATCGTAGACTGAAAGGATGGTCTGAAGCGAAAATAGTAAACT
GTGATGCTGTATCTACTGGACAAGGTGTTTATAATCATGGATATGCTAGTCATATTCAGATTACGCGTGTAAATAA
TGTTATAGAATTGGAAGTCCCGTTTTATAACGCTACGTGCTATAATTATTTGCAAGCGTTTAACCCATCTAGTGCA
GCGTCGAGTTATGCCGTTTCGCTCGGAGAGATTTCGGTTGGTTTTCAGGCTACTAGTGATGATATTGCAGCCATAG
TTAATAAACCTGTAACTATATATTACAGTATTGGCGATGGTATGCAGTTTTCGCAGTGGGTTGGTTATCAACCAAT
GATGATTCTGGATCAATTGCCAGCACCTGTAGTTAGGGCTGTGCCTGAGGGCCCTATAGCGAAGATAAAGAACTTT
TTCCACCAAACGGCAGATGAAGTTCGAGAAGCTCAGGCCGCAAAGATGCGTGAAGATATGGGTATAGTAGTCCAAG
ACGTTATAGGAGAGTTAAGTCAGGCTATACCCGATCTTCAACAACCGGAAGTTCAAGCGAATGTTTTTTCCTTGGT
GTCACAGTTAGTGCATGCTATTATTGGTACTAGTCTTAAGACAGTTGCTTGGGCGATTGTTTCGATTTTTGTAACT
TTAGGTTTGATTGGACGTGAAATGATGCATTCAGTCATAACTGTAGTTAAGCGGTTATTAGAAAAATATCACTTGG
CGACGCAACCCCAGGAATCCGCCAATTCAGGTACGGTTATTTCCGCTGTTCCAGAAGCACCCAATGCTGAAGCAGA
GGAGGCCAGTGCCTGGGTATCCATTATTTATAATGGTGTGTGTAATATGTTGAATGTAGCCGCTCAAAAACCGAAA
CAATTTAAAGATTGGGTAAAATTAGCTACCGTAGATTTTAGTAATAATTGTAGAGGTAGTAATCAGGTATTTGTGT
TTTTCAAGAATACGTTTGAAGTGTTGAAGAAAATGTGGGGTTATGTGTTTTGTCAGAGTAATCCTGCAGCGCGACT
CTTGAAAGCAGTGAATGATGAACCTGAGATTTTAAAAGCGTGGGTTAAAGAATGTCTGTATTTAGATGATCCTAAA
TTTAGAATGCGACGTGCACATGATCAAGAGTATATTGAGAGAGTGTTTGCGGCCCATTCGTATGGACAAATTTTAT
TGCATGACTTAACGGCTGAAATGAATCAATCGCGTAATTTAAGTGTGTTTACGAGAGTGTATGATCAAATATCTAA
ATTGAAGACGGATCTCATGGAAATGGGATCAAACCCATATATCAGGCGTGAATGTTTTACGATTTGTATGTGTGGT
GCATCTGGAATTGGTAAGTCTTATTTAACTGATTCTTTATGCAGCGAGCTCTTACGTGCGAGTCGTACTCCAGTGA
CAACGGGCATTAAGTGTGTTGTGAACCCTTTGTCTGATTATTGGGATCAGTGTGATTTTCAGCCCGTTTTATGTGT
TGATGACATGTGGAGTGTTGAAACGTCTACTACGCTCGATAAACAGTTAAATATGCTATTTCAGGTTCATTCACCA
ATTGTACTTTCACCTCCTAAAGCTGATTTAGAAGGTAAGAAAATGCGTTATAATCCTGAAATATTCATATATAATA
CGAATAAACCTTTTCCGAGGTTTGATCGTATAGCTATGGAAGCTATTTATCGACGTAGAAACGTTTTAATTGAATG
TAAGGCTAATGAAGAGAAGAAGCGTGGATGTAAACATTGTGAGAATAATATACCCATTGCTGAATGTAGTCCAAAA
ATTTTGAAAGATTTTCATCACATTAAATTTCGTTATGCTCATGATGTGTGCAATTCTGAAACTACGTGGTCTGAGT
GGATGTCGTATAATGAATTTTTGGAATGGATTACTCCTGTATATATGGCTAATCGACGTAAAGCAAATGAATCGTT
TAAGATGCGTGTTGATGAAATGCAAATGTTGCGTATAGACGAGCCCTTGGAAGGCGATAATATTTTAAATAAGTAT
GTTGAAGTTAATCAGCGCTTAGTTGAGGAAATGAAAGCTTTTAAAGAGCGAACCCTCTGGGCTGATTTACAACGTG
TTGGCTCAGAGATTAGTACTTCAGTTAAGAAAGCATTACCAACTATTTCCATTACTGAGAAGCTACCACATTGGAC
CATCCAATGTGGCATAGCTAAGCCTGAAATGGATCATGCTTATGAAGTTATGAGCTCATATGCAGCAGGAATGAAC
GCAGAAATTGAAGCGCATGAACAAGTTCGTCGTTCTTCTTTGGAATGTCAGTATGTTGAGCCTTCAACTTCAAGAC
CTCTGGATGAAGAGGGTCCTACTATCGACGAGGAATTACTTGGCGAAGTAGAATTTACTTCTTCAGCTTTGGAGCG
TTTGGTTGATGAGGGGTATATTACTGGTAAACAAAAGAAGTACATGGCAACTTGGTGTACGAAACGAAGAGAGCAT
GTATCTGATTTTGATTTAGTATGGATGGATAATTTGCGTGTTTTAAGTGCGTATGTCCACGAGCGTTCTACATCTA
CGCGTTTGTCTACCGATGATGTTAAATTATTTAAGACGATTAGTATGTTACATCAGAGGTATGATACCACTGATTG
TGCAAAATGCCAACATTGGTATGCACCATTAACAGCTATTTATGTTGATGATAGAAAGCTGTTTTGGTGCCAGAAG
GAGACTAAGACTTTGATAGATGTTCGTAAATTGTCGAAAGAGGACGTTACAGTCCAATCGAAATTAATTAACTTAT
CGGTTCCGTGCGGTGATGTATGTATATTACATTCTAAGTACTTTAATTATTTATTTCATAAAGCGTGGTTGTTTGA
AAATCCAACATGGCGTTTAATATATAACGGTACTAAGAAAGGTATGCCTGAGTATTTCATGAATTGCGTGGATGAA
ATTTCATTAGATTCAAAATTTTGTAAAGTAAAGGTTTGGCTTCAAGCAATTATTGATAAATATTTGACTCGTCCAG
TGAAAATGATTCGTGACTTTCTATTTAAATGGTGGCCGCAAGTAGCATACGTGTTAAGTTTGTTAGGTATAATTGG
TATAACTGCGTATGAGATGCGTAATCCTAAATCAACAGCAGAAGACTTGGCTGAGCACTATGTTAATAGGCATTGT
AGTTCAGATTTTTGGTCACCAGGTATGGCAACTCCTCAGGGATTAAAATATAGTGAAGCGATAACAGCTAAAGCGC
CTAGAATTCATAGATTGCCCGTTACTACTAGACCTCAGGGATCAACGCAACAAGTTGACGCCGCTGTGAATAAAAT
TTTGCAGAATATGGTGTATATCGGTGTTGTGTTTCCAAAAGTGCCTGGTAGTAAGTGGCGAGATATTAATTTTAGA
TGTCTTATGCTTCATAATCGGCAGTGTTTGATGTTGCGGCATTACATTGAGTCGACGGCTGCTTTTCCGGAGGGTA
CCAAATACTATTTTAAGTATATTCATAATCAAGAAACTCGAATGTCAGGTGATATATCTGGTATTGAGATTGATTT
ATTGAGTTTACCTAGATTGTATTATGGTGGCTTAGCCGGGGAAGAGTCGTTCGATAGCAATATAGTGTTAGTAACT
ATGCCGAATAGAATTCCTGAGTGTAAGAGTATTGTGAAGTTTATAGCTTCACATGCTGAACATGCTCGTGCTCAAA
ATGATGGTGTGTTAGTTACTGGTGAACATACTCAGTTATTGGCGTTTGAGAATAATAATAAAACACCTATAAGTAT
TAATGCTGATGGTTTGTATGAGGTTATACTTCAAGGAGTATACACCTATCCATACCATGGTGATGGTGTTTGTGGG
TCTATATTATTGTCTCGTAATTTACAACGACCGATTATAGGGATCCATGTAGCTGGTACTGAAGGATTACATGGCT
TTGGTGTTGCTGAACCTCTTGTTCATGAGATGTTCACTGGGAAAGCAATAGAGAGTGAAAGGGAACCGTATGATCG
TGTGTATGAATTACCTTTGCGTGAATTAGATGAATCTGATATAGGTTTAGATACTGATTTATATCCTATAGGAAGA
GTCGATGCGAAATTAGCTCATGCCCAAAGTCCTTCAACAGGAATTAAAAAGACGCTTATTCATGGTACTTTTGATG
TTCGGACTGAACCGAATCCGATGTCATCACGAGACCCAAGAATAGCGCCACATGATCCGTTGAAGTTGGGGTGTGA
GAAACATGGTATGCCATGTTCTCCATTTAATCGGAAACATTTGGAATTAGCAACAACTCATTTAAAGGAGAAGTTA

ATTTCCGTAGTTAAACCTATAAATGGATGCAAGATTAGAAGTTTGCAAGATGCTGTATGTGGTGTACCAGGTTTGG
ATGGCTTTGATTCAATATCCTGGAATACTAGTGCTGGTTTTCCTTTATCTTCATTAAAACCGCCAGGCTCTTCTGG
TAAGCGATGGTTGTTTGATATTGAATTACAAGATTCAGGATGTTATCTTTTGAGAGGGATGAGACCTGAACTTGAG
ATACAGTTGACAACAACTCAGTTAATGAGGAAGAAGGGAATAAAGCCTCACACTATATTCACAGATTGTTTGAAAG
ATACATGTTTGCCTGTGGAAAAATGCAGAATACCTGGTAAAACTAGAATATTTAGTATAAGTCCCGTCCAATTTAC
GATTCCATTTCGACAATACTATCTCGATTTTATGGCGTCGTACCGTGCCGCTAGACTTAATGCTGAGCATGGAATA
GGTATAGACGTGAACAGCTTGGAATGGACAAACTTGGCAACAAGTCTGTCGAAATATGGCACGCATATTGTGACAG
GAGATTATAAGAATTTTGGTCCTGGGTTAGATTCTGATGTTGCCGCTTCAGCTTTTGAAATTATCATTGATTGGGT
GTTAAATTACACTGAAGAAGATGATAAAGACGAAATGAAGCGTGTAATGTGGACTATGGCTCAGGAAATCTTAGCT
CCTAGTCACTTATGTCGTGATTTAGTATATCGCGTGCCATGCGGTATTCCTTCTGGATCACCGATTACGGACATTT
TGAATACTATTTCGAATTGTTTGTTAATTCGATTGGCTTGGCAAGGTATTACTGATTTGCCTTTATCCGAATTTTC
TAGACATGTCGTGCTAGTTTGTTACGGTGATGATCTCATCATGAATGTAAGTGATGAGATGATAGATAAATTCAAT
GCTGTAACAATTGGCGATTTCTTTTCGCGATATAAGATGGAATTTACGGATCAGGATAAGTCTGGAAATACAGTGC
GGTGGCGAACTTTACAAACTGCTACGTTTTTGAAGCATGGGTTCTTGAAACATCCAACAAGACCCGTGTTTCTAGC
CAATCTGGATAAGGTTTCTATAGAAGGAACAACCAATTGGACACATGCTCGAGGATTGGGTCGTCGAGTAGCAACC
ATTGAGAATGCTAAACAAGCGCTAGAGTTGGCATTCGGATGGGGTCCCGAATACTTCAATCATGTTCGGAATACCA
TTAAAATGGCATTCGACAAGTTAGGTATTTATGAGGATCTCATAACGTGGGAAGAAATGGATGTTAGATGTTATGC
TAGTGCGTAATTTTAAGATTTTAATACTCATTAAAATTAATTCATATTTAGGTTATTGGAATTGAGGGAAGTACCA
CCCCCCAAGACCTTCGTTTTAAATCTGCTAAGAGGAGTGAACTTGCATATAAGAGTCTAAAAGCAGAGTGGATTAG
ACCACCACTTTTAGCTTATATGTGAGGAAGGTTGAGTTGCCTCTAAAGACTCAGCTCCGTAGTAGAGTAGTTTTAG
TTACGATTAAAGTGGTACTCTAGGTTAGGTGTTACTCGCGTATTGTCGCATAACGGCAATGCGTCCTAATTTTAGT
ATAGTTTAACCATAATAG

>VDV1-USA-MD-7-cons

GGCGATTTATGCCTTCCATAGCGAATTACGGTGCAACTAACAATTTTAGATAGTAGCCATGAACAAACATTATGAT
TACTCACTACGTATTGATCATTTTTACAATGACTTGCGTAGCATGAAGCGCATGCTTGTAGTTATAACTATGTTAT
TTTGCAAGTTGGAGATAATTGTATTGGATTATGGATGCGTGCACTAAGTGTCTACATCTATAGTCGTTTGTGGTTC
AAGTTTTTGTGTTAGTAGTACAATTTTGAAGAATGTAAGTATCGTATGAATGATATTTGAATGACAACACTGAAGT
ATAAAATATATAAAATCCAAAAATATTTTAAATTTTATTCAGTGTAGTGTTTAATAGAGTAGAATGCCATGTGACC
GCTCAAAGAAGTCCATTATGGTATATCATTCGAAGTTGAATACTTGTGTATAGTTATTGTATTTTATTAGTAATAT
TAGTAGTCCGTAACTATCATAATCCTATTATAGTTTGATTATATGATAGACCACTGCAGTATCGAGTAGAGTTTAG
AAAGAGTAGTGCAATAGTAAGATCACTGTCACCGACCACTTATTGTAATAGTGAGGTTTGTCGGAAACCAGTTATT
GTGCAGCGACTAGCAATCGTGAATCAATATAGTTGGTATTCTAAATATGAGACGATTCGGCGATTTTATTGCGACT
GAAATTTCATATTTAGCATGTCAGGTCTTATTATGAATGCTCGAGTATTTATTTCTGCGGTAGAGTAGGGACCCCT
CTATCTTTCAGGTACTGTATGAGGCGAAAGTGTGAAAGTAATTTATGTCTCTATACATAAGTGACTGTATCGGGAT
TTCCTTTGGCAAGAATCCTTTTAATACAGTATAATTTATGCTACGGTACGTTACGTTCGCAGGGCACCCGTTAATG
TCACATAGCCCAGACGATGACGAATGGAAAGACATTACTTTTTATTTTAATGCTACGATTATTGCTGTTTTATTTT
GCTGTTTTTATTTGCTATTATATTTTGCTATTTTATTATTGCTAAATATATTTCTTTGCTATTTTTGCTTTATATA
TTAGATTCAATTCTTTTTATTTTATATTTTCAATTTGATTTCGATTTTGAAGGTAAATATATATAAAAATGGCATT
TAGTTGTGGAACTCTTTCTTATGCTGCTGTTGCTCAAGCTCCCTCTGTAGCTCATGCTCCCCGTAGTTGGGAGATT
GATGAAGCTAGGCGTCGACGTGTTATTAAGCGTTTGGCGTTGGAACAGGAACGGATTCGAAACGTTCTTGATGTCA
CTGTGTATGATCATACAACGTGGGAGCAAGAGGATGCGCGTGATAATGAGTTCCTTACGGAACAATTGAATAATTT
ATATACGATATATTCTATAGCTGAAAGATGTGCCCGCCGCCCTGTTCAAGAACATGTCCCCATTTCAATCAGTAAT
AGATATTCCCCTTTAGAATCCCTTAAGATTGAGGTAGGACAAGATGCGGGTGAGTTCGTATTTAAGAGACCCAAAT
ATACAAAGATTTGTAAGAAAGTGAAACGGGTGGCATCAAAATTTGTGCGCGAGAAAGTTGTTAGGCCCGTTTGTAA
TCGATCGCCCATGTTATTATTTAAAATTAAGAAAGTAATATATGATTTACATTTGTATCGGTTACGGAAACAAGTT
CGGCTTCTCAGACGCGAAAAACAGCGTGAATATGAGTTAGAGTGTGTTACTAGTTTGCTACAGCTATCTAATCCTG
TTTCAGCTAAACCTGAGATGGACAATCCTAATCCTGGTCCAGATGGTGAAGGTGAAGTTGAATTAGAAAAAGATAG
TAATGTAGTATTAACTACACAACGTGATCCTAGTACCTCTATTCCTGCTCCAACTAGTGTGAAGTGGAGTAGGTGG
ACTAGTAATGATGTTGTGGATGATTATGCCACTATAACTTCGCGTTGGTATCAGATTGCCGAATTTGTATGGTCAA
AGGATGATCCATTTGATAAGGAATTGGCGCGCTTAATTTTACCTCGAGCTTTGTTATCTAGTATTGAGGCTAATTC
TGACGCTATTTGTGATGTACCTAATACTATTCCGTTTAAGGTACATGCATATTGGCGTGGAGATATGGAAGTTCGA
GTGCAGATTAACTCGAATAAATTCCAGGTTGGTCAATTACAGGCAACTTGGTATTATTCGGATCATGAAAATTTGA
ATATTCAGACGAAGCGAAGTGTGTATGGTTTTTCGCATATGGATCATGCTTTGATTAGTGCATCAGCGAGTAATGA
AGCAAAATTAGTGATACCTTTTAAACACGTATATCCATTCTTACCAACGCGCGTCGTTCCTGATTGGACAACTGGT
ATTCTTGATATGGGTACCTTAAATATTCGTGTAATTGCTCCATTACGTATGAGTGCGACGGGACCAACCACTTGTA
ATGTTGTAGTATTTATTAAGTTAAATAATAGTGAATTCACTGGTACTTCCTCTGGTAAGTTTTACGCGAATCAAAT
CAGGGCAAAACCTGAAATGGACCGTGTGTTAAATTTGGCAGAAGGATTATTAAATAATACTGTAGGTGGTTGTAAT

ATGGATAATCCGTCATATCAGCAATCTCCGCGTCATTTTGTTCCTACTGGTATGCATAGTTTAGCTTTAGGCACTA
ATTTAGTAGAGCCTTTGCATGCATTACGATTAGATGCATCAGGTACAACACAACATCCAGTTGGGTGTGCGCCTGA
TGAAGATATGACTGTATCTTCCATTGCATCACGATATGGTTTAATTCGCCAAGTGCAATGGAAGAAAGACCATGCG
AAAGGATCATTATTATTACAACTTGATGCTGATCCTTTCGTTGAACAGAAAATTGAGGGAACCAATCCAATTTCTT
TGTATTGGTTTGCTCCGGTTGGAGTTGTATCTAGTATGTTTATGCAATGGAGAGGTTCTTTAGAATATAGATTTGA
TATTATAGCTTCCCAATTTCATACGGGTAGGTTAATTGTAGGTTATGTTCCTGGACTGACTGCTTCTTTACAACGT
CAAATGGACTATATGAAATTGAAATCATCTAGTTATGTAGTGTTTGATTTACAGGAAAGTAATAGTTTTACGTTTG
AAGTGCCTTATGTGTCATACAGACCGTGGTGGGTGCGTAAGTATGGTGGTAATTATCTGCCATCCTCTACTGATGC
GCCTAGCACACTGTTTATGTATGTACAAGTGCCATTGATACCTATGGAAGCTGTTTCTGATACTATAGATATCAAT
GTGTATGTGCGTGGTGGCAGTTCGTTTGAGGTTTGTGTTCCAGTCCAACCTAGTTTAGGTTTGAACTGGAATACAG
ATTTTATATTACGTAATGATGAGGAGTACCGCGCAAAGAATGGATATGCACCATATTATGCTGGTGTGTGGCATAG
CTTCAATAATAGTAATTCGCTTATTTTTAGATGGGGTTCGGCTTCAGATCAAATTGCTCAATGGCCAACAATAACA
GTGCCTCGAGGAGAGTTGGCATTCTTGCGTATCCGCGATGCTAAGCAAGCTGCTGTAGGAACGCAACCTTGGCGTA
CTATGGTCGTTTGGCCTTCAGGTCATGGATATAATATTGGAATACCAACTTATAATGCTGAACGAGCAAGACAACT
TGCTCAGCATTTGTATGGTGGTGGGTCTTTGACAGATGAAAAGGCTAAGCAATTATTTGTGCCTGCTAACCAGCAA
GGACCCGGCAAAGTAAGTAATGGTAACCCCGTCTGGGAAGTAATGCGCGCGCCTCTTGCAACTCAGCAAGCGCATA
TACAAGATTTTGAATTTGTTGAAGCTGTTCCAGAAGGCGAAGAATCACGCAACACTACAGTGCTAGACACGACAAC
AACGTTACAGTCTAGCGGATTTGGTCGTGCTTTCTTTGGTGAGGCATTTAACGATCTTAAGACGTTAATGCGCCGA
TACCAATTATATGGTCAATTATTGTTATCCGTTACTACGGATAAGGATATTGATCATTGTATGTTTACCTTCCCTT
GTTTACCTCAAGGGTTAGCGTTAGATATAGGTTCGGCTGGATCTCCTCATGAAATATTTAATCGCTGCCGTGATGG
TATCATTCCATTGATAGCGTCAGGGTATCGGTTTTATCGAGGCGACTTACGGTTCAAAATTGTTTTCCCAAGTAAC
GTTAATAGCAATATTTGGGTACAACATCGACCAGATCGTAGACTGAAAGGATGGTCTGAAGCGAAAATAGTAAACT
GTGATGCTGTATCTACTGGACAAGGTGTTTATAATCATGGATATGCTAGTCATATTCAGATTACGCGTGTAAATAA
TGTTATAGAATTGGAAGTCCCGTTTTATAACGCTACGTGCTATAATTATTTGCAAGCGTTTAACCCATCTAGTGCA
GCGTCGAGTTATGCCGTTTCGCTCGGAGAGATTTCGGTTGGTTTTCAGGCTACTAGTGATGATATTGCAGCCATAG
TTAATAAACCTGTAACTATATATTACAGTATTGGCGATGGTATGCAGTTTTCGCAGTGGGTTGGTTATCAACCAAT
GATGATTCTGGATCAATTGCCAGCACCTGTAGTTAGGGCTGTGCCTGAGGGCCCTATAGCGAAGATAAAGAACTTT
TTCCACCAAACGGCAGATGAAGTTCGAGAAGCTCAGGCCGCAAAGATGCGTGAAGATATGGGTATAGTAGTCCAAG
ACGTTATAGGAGAGTTAAGTCAGGCTATACCCGATCTTCAACAACCGGAAGTTCAAGCGAATGTTTTTTCCTTGGT
GTCACAGTTAGTGCATGCTATTATTGGTACTAGTCTTAAGACAGTTGCTTGGGCGATTGTTTCGATTTTTGTAACT
TTAGGTTTGATTGGACGTGAAATGATGCATTCAGTCATAACTGTAGTTAAGCGGTTATTAGAAAAATATCACTTGG
CGACGCAACCCCAGGAATCCGCCAATTCAGGTACGGTTATTTCCGCTGTTCCAGAAGCACCCAATGCTGAAGCAGA
GGAGGCCAGTGCCTGGGTATCCATTATTTATAATGGTGTGTGTAATATGTTGAATGTAGCCGCTCAAAAACCGAAA
CAATTTAAAGATTGGGTAAAATTAGCTACCGTAGATTTTAGTAATAATTGTAGAGGTAGTAATCAGGTATTTGTGT
TTTTCAAGAATACGTTTGAAGTGTTGAAGAAAATGTGGGGTTATGTGTTTTGTCAGAGTAATCCTGCAGCGCGACT
CTTGAAAGCAGTGAATGATGAACCTGAGATTTTAAAAGCGTGGGTTAAAGAATGTCTGTATTTAGATGATCCTAAA
TTTAGAATGCGACGTGCACATGATCAAGAGTATATTGAGAGAGTGTTTGCGGCCCATTCGTATGGACAAATTTTAT
TGCATGACTTAACGGCTGAAATGAATCAATCGCGTAATTTAAGTGTGTTTACGAGAGTGTATGATCAAATATCTAA
ATTGAAGACGGATCTCATGGAAATGGGATCAAACCCATATATCAGGCGTGAATGTTTTACGATTTGTATGTGTGGT
GCATCTGGAATTGGTAAGTCTTATTTAACTGATTCTTTATGCAGCGAGCTCTTACGTGCGAGTCGTACTCCAGTGA
CAACGGGCATTAAGTGTGTTGTGAACCCTTTGTCTGATTATTGGGATCAGTGTGATTTTCAGCCCGTTTTATGTGT
TGATGACATGTGGAGTGTTGAAACGTCTACTACGCTCGATAAACAGTTAAATATGCTATTTCAGGTTCATTCACCA
ATTGTACTTTCACCTCCTAAAGCTGATTTAGAAGGTAAGAAAATGCGTTATAATCCTGAAATATTCATATATAATA
CGAATAAACCTTTTCCGAGGTTTGATCGTATAGCTATGGAAGCTATTTATCGACGTAGAAACGTTTTAATTGAATG
TAAGGCTAATGAAGAGAAGAAGCGTGGATGTAAACATTGTGAGAATAATATACCCATTGCTGAATGTAGTCCAAAA
ATTTTGAAAGATTTTCATCACATTAAATTTCGTTATGCTCATGATGTGTGCAATTCTGAAACTACGTGGTCTGAGT
GGATGTCGTATAATGAATTTTTGGAATGGATTACTCCTGTATATATGGCTAATCGACGTAAAGCAAATGAATCGTT
TAAGATGCGTGTTGATGAAATGCAAATGTTGCGTATAGACGAGCCCTTGGAAGGCGATAATATTTTAAATAAGTAT
GTTGAAGTTAATCAGCGCTTAGTTGAGGAAATGAAAGCTTTTAAAGAGCGAACCCTCTGGGCTGATTTACAACGTG
TTGGCTCAGAGATTAGTACTTCAGTTAAGAAAGCATTACCAACTATTTCCATTACTGAGAAGCTACCACATTGGAC
CATCCAATGTGGCATAGCTAAGCCTGAAATGGATCATGCTTATGAAGTTATGAGCTCATATGCAGCAGGAATGAAC
GCAGAAATTGAAGCGCATGAACAAGTTCGTCGTTCTTCTTTGGAATGTCAGTATGTTGAGCCTTCAACTTCAAGAC
CTCTGGATGAAGAGGGTCCTACTATCGACGAGGAATTACTTGGCGAAGTAGAATTTACTTCTTCAGCTTTGGAGCG
TTTGGTTGATGAGGGGTATATTACTGGTAAACAAAAGAAGTACATGGCAACTTGGTGTACGAAACGAAGAGAGCAT
GTATCTGATTTTGATTTAGTATGGATGGATAATTTGCGTGTTTTAAGTGCGTATGTCCACGAGCGTTCTACATCTA
CGCGTTTGTCTACCGATGATGTTAAATTATTTAAGACGATTAGTATGTTACATCAGAGGTATGATACCACTGATTG
TGCAAAATGCCAACATTGGTATGCACCATTAACAGCTATTTATGTTGATGATAGAAAGCTGTTTTGGTGCCAGAAG
GAGACTAAGACTTTGATAGATGTTCGTAAATTGTCGAAAGAGGACGTTACAGTCCAATCGAAATTAATTAACTTAT
CGGTTCCGTGCGGTGATGTATGTATATTACATTCTAAGTACTTTAATTATTTATTTCATAAAGCGTGGTTGTTTGA

AAATCCAACATGGCGTTTAATATATAACGGTACTAAGAAAGGTATGCCTGAGTATTTCATGAATTGCGTGGATGAA
ATTTCATTAGATTCAAAATTTTGTAAAGTAAAGGTTTGGCTTCAAGCAATTATTGATAAATATTTGACTCGTCCAG
TGAAAATGATTCGTGACTTTCTATTTAAATGGTGGCCGCAAGTAGCATACGTGTTAAGTTTGTTAGGTATAATTGG
TATAACTGCGTATGAGATGCGTAATCCTAAATCAACAGCAGAAGACTTGGCTGAGCACTATGTTAATAGGCATTGT
AGTTCAGATTTTTGGTCACCAGGTATGGCAACTCCTCAGGGATTAAAATATAGTGAAGCGATAACAGCTAAAGCGC
CTAGAATTCATAGATTGCCCGTTACTACTAGACCTCAGGGATCAACGCAACAAGTTGACGCCGCTGTGAATAAAAT
TTTGCAGAATATGGTGTATATCGGTGTTGTGTTTCCAAAAGTGCCTGGTAGTAAGTGGCGAGATATTAATTTTAGA
TGTCTTATGCTTCATAATCGGCAGTGTTTGATGTTGCGGCATTACATTGAGTCGACGGCTGCTTTTCCGGAGGGTA
CCAAATACTATTTTAAGTATATTCATAATCAAGAAACTCGAATGTCAGGTGATATATCTGGTATTGAGATTGATTT
ATTGAGTTTACCTAGATTGTATTATGGTGGCTTAGCCGGGGAAGAGTCGTTCGATAGCAATATAGTGTTAGTAACT
ATGCCGAATAGAATTCCTGAGTGTAAGAGTATTGTGAAGTTTATAGCTTCACATGCTGAACATGCTCGTGCTCAAA
ATGATGGTGTGTTAGTTACTGGTGAACATACTCAGTTATTGGCGTTTGAGAATAATAATAAAACACCTATAAGTAT
TAATGCTGATGGTTTGTATGAGGTTATACTTCAAGGAGTATACACCTATCCATACCATGGTGATGGTGTTTGTGGG
TCTATATTATTGTCTCGTAATTTACAACGACCGATTATAGGGATCCATGTAGCTGGTACTGAAGGATTACATGGCT
TTGGTGTTGCTGAACCTCTTGTTCATGAGATGTTCACTGGGAAAGCAATAGAGAGTGAAAGGGAACCGTATGATCG
TGTGTATGAATTACCTTTGCGTGAATTAGATGAATCTGATATAGGTTTAGATACTGATTTATATCCTATAGGAAGA
GTCGATGCGAAATTAGCTCATGCCCAAAGTCCTTCAACAGGAATTAAAAAGACGCTTATTCATGGTACTTTTGATG
TTCGGACTGAACCGAATCCGATGTCATCACGAGACCCAAGAATAGCGCCACATGATCCGTTGAAGTTGGGGTGTGA
GAAACATGGTATGCCATGTTCTCCATTTAATCGGAAACATTTGGAATTAGCAACAACTCATTTAAAGGAGAAGTTA
ATTTCCGTAGTTAAACCTATAAATGGATGCAAGATTAGAAGTTTGCAAGATGCTGTATGTGGTGTACCAGGTTTGG
ATGGCTTTGATTCAATATCCTGGAATACTAGTGCTGGTTTTCCTTTATCTTCATTAAAACCGCCAGGCTCTTCTGG
TAAGCGATGGTTGTTTGATATTGAATTACAAGATTCAGGATGTTATCTTTTGAGAGGGATGAGACCTGAACTTGAG
ATACAGTTGACAACAACTCAGTTAATGAGGAAGAAGGGAATAAAGCCTCACACTATATTCACAGATTGTTTGAAAG
ATACATGTTTGCCTGTGGAAAAATGCAGAATACCTGGTAAAACTAGAATATTTAGTATAAGTCCCGTCCAATTTAC
GATTCCATTTCGACAATACTATCTCGATTTTATGGCGTCGTACCGTGCCGCTAGACTTAATGCTGAGCATGGAATA
GGTATAGACGTGAACAGCTTGGAATGGACAAACTTGGCAACAAGTCTGTCGAAATATGGCACGCATATTGTGACAG
GAGATTATAAGAATTTTGGTCCTGGGTTAGATTCTGATGTTGCCGCTTCAGCTTTTGAAATTATCATTGATTGGGT
GTTAAATTACACTGAAGAAGATGATAAAGACGAAATGAAGCGTGTAATGTGGACTATGGCTCAGGAAATCTTAGCT
CCTAGTCACTTATGTCGTGATTTAGTATATCGCGTGCCATGCGGTATTCCTTCTGGATCACCGATTACGGACATTT
TGAATACTATTTCGAATTGTTTGTTAATTCGATTGGCTTGGCAAGGTATTACTGATTTGCCTTTATCCGAATTTTC
TAGACATGTCGTGCTAGTTTGTTACGGTGATGATCTCATCATGAATGTAAGTGATGAGATGATAGATAAATTCAAT
GCTGTAACAATTGGCGATTTCTTTTCGCGATATAAGATGGAATTTACGGATCAGGATAAGTCTGGAAATACAGTGC
GGTGGCGAACTTTACAAACTGCTACGTTTTTGAAGCATGGGTTCTTGAAACATCCAACAAGACCCGTGTTTCTAGC
CAATCTGGATAAGGTTTCTATAGAAGGAACAACCAATTGGACACATGCTCGAGGATTGGGTCGTCGAGTAGCAACC
ATTGAGAATGCTAAACAAGCGCTAGAGTTGGCATTCGGATGGGGTCCCGAATACTTCAATCATGTTCGGAATACCA
TTAAAATGGCATTCGACAAGTTAGGTATTTATGAGGATCTCATAACGTGGGAAGAAATGGATGTTAGATGTTATGC
TAGTGCGTAATTTTAAGATTTTAATACTCATTAAAATTAATTCATATTTAGGTTATTGGAATTGAGGGAAGTACCA
CCCCCCAAGACCTTCGTTTTAAATCTGCTAAGAGGAGTGAACTTGCATATAAGAGTCTAAAAGCAGAGTGGATTAG
ACCACCACTTTTAGCTTATATGTGAGGAAGGTTGAGTTGCCTCTAAAGACTCAGCTCCGTAGTAGAGTAGTTTTAG
TTACGATTAAAGTGGTACTCTAGGTTAGGTGTTACTCGCGTATTGTCGCATAACGGCAATGCGTCCTAATTTTAGT
ATAGTTTAACC

>VDV1-USA-MD-12-cons
ATGGGAGGCGATTTATGCCTTCCATAGCGAATTACGGTGCAACTAACAATTTTAGATAGTAGCCATGAACAAACAT
TATGATTACTCACTACGTATTGATCATTTTTACAATGACTTGCGTAGCATGAAGCGCATGCTTGTAGTTATAACTA
TGTTATTTTGCAAGTTGGAGATAATTGTATTGGATTATGGATGCGTGCACTAAGTGTCTACATCTATAGTCGTTTG
TGGTTCAAGTTTTTGTGTTAGTAGTACAATTTTGAAGAATGTAAGTATCGTATGAATGATATTTGAATGACAACAC
TGAAGTATAAAATATATAAAATCCAAAAATATTTTAAATTTTATTCAGTGTAGTGTTTAATAGAGTAGAATGCCAT
GTGACCGCTCAAAGAAGTCCATTATGGTATATCATTCGAAGTTGAATACTTGTGTATAGTTATTGTATTTTATTAG
TAATATTAGTAGTCCGTAACTATCATAATCCTATTATAGTTTGATTATATGATAGACCACTGCAGTATCGAGTAGA
GTTTAGAAAGAGTAGTGCAATAGTAAGATCACTGTCACCGACCACTTATTGTAATAGTGAGGTTTGTCGGAAACCA
GTTATTGTGCAGCGACTAGCAATCGTGAATCAATATAGTTGGTATTCTAAATATGAGACGATTCGGCGATTTTATT
GCGACTGAAATTTCATATTTAGCATGTCAGGTCTTATTATGAATGCTCGAGTATTTATTTCTGCGGTAGAGTAGGG
ACCCCTCTATCTTTCAGGTACTGTATGAGGCGAAAGTGTGAAAGTAATTTATGTCTCTATACATAAGTGACTGTAT
CGGGATTTCCTTTGGCAAGAATCCTTTTAATACAGTATAATTTATGCTACGGTACGTTACGTTCGCAGGGCACCCG
TTAATGTCACATAGCCCAGACGATGACGAATGGAAAGACATTACTTTTTATTTTAATGCTACGATTATTGCTGTTT
TATTTTGCTGTTTTTATTTGCTATTATATTTTGCTATTTTATTATTGCTAAATATATTTCTTTGCTATTTTTGCTT
TATATATTAGATTCAATTCTTTTTATTTTATATTTTCAATTTGATTTCGATTTTGAAGGTAAATATATATATAAAAT

GGCATTTAGTTGTGGAACTCTTTCTTATGCTGCTGTTGCTCAAGCTCCCTCTGTAGCTCATGCTCCCCGTAGTTGG
GAGATTGATGAAGCTAGGCGTCGACGTGTTATTAAGCGTTTGGCGTTGGAACAGGAACGGATTCGAAACGTTCTTG
ATGTCACTGTGTATGATCATACAACGTGGGAGCAAGAGGATGCGCGTGATAATGAGTTCCTTACGGAACAATTGAA
TAATTTATATACGATATATTCTATAGCTGAAAGATGTGCCCGCCGCCCTGTTCAAGAACATGTCCCCATTTCAATC
AGTAATAGATATTCCCCTTTAGAATCCCTTAAGATTGAGGTAGGACAAGATGCGGGTGAGTTCGTATTTAAGAGAC
CCAAATATACAAAGATTTGTAAGAAAGTGAAACGGGTGGCATCAAAATTTGTGCGCGAGAAAGTTGTTAGGCCCGT
TTGTAATCGATCGCCCATGTTATTATTTAAAATTAAGAAAGTAATATATGATTTACATTTGTATCGGTTACGGAAA
CAAGTTCGGCTTCTCAGACGCGAAAAACAGCGTGAATATGAGTTAGAGTGTGTTACTAGTTTGCTACAGCTATCTA
ATCCTGTTTCAGCTAAACCTGAGATGGACAATCCTAATCCTGGTCCAGATGGTGAAGGTGAAGTTGAATTAGAAAA
AGATAGTAATGTAGTATTAACTACACAACGTGATCCTAGTACCTCTATTCCTGCTCCAACTAGTGTGAAGTGGAGT
AGGTGGACTAGTAATGATGTTGTGGATGATTATGCCACTATAACTTCGCGTTGGTATCAGATTGCCGAATTTGTAT
GGTCAAAGGATGATCCATTTGATAAGGAATTGGCGCGCTTAATTTTACCTCGAGCTTTGTTATCTAGTATTGAGGC
TAATTCTGACGCTATTTGTGATGTACCTAATACTATTCCGTTTAAGGTACATGCATATTGGCGTGGAGATATGGAA
GTTCGAGTGCAGATTAACTCGAATAAATTCCAGGTTGGTCAATTACAGGCAACTTGGTATTATTCGGATCATGAAA
ATTTGAATATTCAGACGAAGCGAAGTGTGTATGGTTTTTCGCATATGGATCATGCTTTGATTAGTGCATCAGCGAG
TAATGAAGCAAAATTAGTGATACCTTTTAAACACGTATATCCATTCTTACCAACGCGCGTCGTTCCTGATTGGACA
ACTGGTATTCTTGATATGGGTACCTTAAATATTCGTGTAATTGCTCCATTACGTATGAGTGCGACGGGACCAACCA
CTTGTAATGTTGTAGTATTTATTAAGTTAAATAATAGTGAATTCACTGGTACTTCCTCTGGTAAGTTTTACGCGAA
TCAAATCAGGGCAAAACCTGAAATGGACCGTGTGTTAAATTTGGCAGAAGGATTATTAAATAATACTGTAGGTGGT
TGTAATATGGATAATCCGTCATATCAGCAATCTCCGCGTCATTTTGTTCCTACTGGTATGCATAGTTTAGCTTTAG
GCACTAATTTAGTAGAGCCTTTGCATGCATTACGATTAGATGCATCAGGTACAACACAACATCCAGTTGGGTGTGC
GCCTGATGAAGATATGACTGTATCTTCCATTGCATCACGATATGGTTTAATTCGCCAAGTGCAATGGAAGAAAGAC
CATGCGAAAGGATCATTATTATTACAACTTGATGCTGATCCTTTCGTTGAACAGAAAATTGAGGGAACCAATCCAA
TTTCTTTGTATTGGTTTGCTCCGGTTGGAGTTGTATCTAGTATGTTTATGCAATGGAGAGGTTCTTTAGAATATAG
ATTTGATATTATAGCTTCCCAATTTCATACGGGTAGGTTAATTGTAGGTTATGTTCCTGGACTGACTGCTTCTTTA
CAACGTCAAATGGACTATATGAAATTGAAATCATCTAGTTATGTAGTGTTTGATTTACAGGAAAGTAATAGTTTTA
CGTTTGAAGTGCCTTATGTGTCATACAGACCGTGGTGGGTGCGTAAGTATGGTGGTAATTATCTGCCATCCTCTAC
TGATGCGCCTAGCACACTGTTTATGTATGTACAAGTGCCATTGATACCTATGGAAGCTGTTTCTGATACTATAGAT
ATCAATGTGTATGTGCGTGGTGGCAGTTCGTTTGAGGTTTGTGTTCCAGTCCAACCTAGTTTAGGTTTGAACTGGA
ATACAGATTTTATATTACGTAATGATGAGGAGTACCGCGCAAAGAATGGATATGCACCATATTATGCTGGTGTGTG
GCATAGCTTCAATAATAGTAATTCGCTTATTTTTAGATGGGGTTCGGCTTCAGATCAAATTGCTCAATGGCCAACA
ATAACAGTGCCTCGAGGAGAGTTGGCATTCTTGCGTATCCGCGATGCTAAGCAAGCTGCTGTAGGAACGCAACCTT
GGCGTACTATGGTCGTTTGGCCTTCAGGTCATGGATATAATATTGGAATACCAACTTATAATGCTGAACGAGCAAG
ACAACTTGCTCAGCATTTGTATGGTGGTGGGTCTTTGACAGATGAAAAGGCTAAGCAATTATTTGTGCCTGCTAAC
CAGCAAGGACCCGGCAAAGTAAGTAATGGTAACCCCGTCTGGGAAGTAATGCGCGCGCCTCTTGCAACTCAGCAAG
CGCATATACAAGATTTTGAATTTGTTGAAGCTGTTCCAGAAGGCGAAGAATCACGCAACACTACAGTGCTAGACAC
GACAACAACGTTACAGTCTAGCGGATTTGGTCGTGCTTTCTTTGGTGAGGCATTTAACGATCTTAAGACGTTAATG
CGCCGATACCAATTATATGGTCAATTATTGTTATCCGTTACTACGGATAAGGATATTGATCATTGTATGTTTACCT
TCCCTTGTTTACCTCAAGGGTTAGCGTTAGATATAGGTTCGGCTGGATCTCCTCATGAAATATTTAATCGCTGCCG
TGATGGTATCATTCCATTGATAGCGTCAGGGTATCGGTTTTATCGAGGCGACTTACGGTTCAAAATTGTTTTCCCA
AGTAACGTTAATAGCAATATTTGGGTACAACATCGACCAGATCGTAGACTGAAAGGATGGTCTGAAGCGAAAATAG
TAAACTGTGATGCTGTATCTACTGGACAAGGTGTTTATAATCATGGATATGCTAGTCATATTCAGATTACGCGTGT
AAATAATGTTATAGAATTGGAAGTCCCGTTTTATAACGCTACGTGCTATAATTATTTGCAAGCGTTTAACCCATCT
AGTGCAGCGTCGAGTTATGCCGTTTCGCTCGGAGAGATTTCGGTTGGTTTTCAGGCTACTAGTGATGATATTGCAG
CCATAGTTAATAAACCTGTAACTATATATTACAGTATTGGCGATGGTATGCAGTTTTCGCAGTGGGTTGGTTATCA
ACCAATGATGATTCTGGATCAATTGCCAGCACCTGTAGTTAGGGCTGTGCCTGAGGGCCCTATAGCGAAGATAAAG
AACTTTTTCCACCAAACGGCAGATGAAGTTCGAGAAGCTCAGGCCGCAAAGATGCGTGAAGATATGGGTATAGTAG
TCCAAGACGTTATAGGAGAGTTAAGTCAGGCTATACCCGATCTTCAACAACCGGAAGTTCAAGCGAATGTTTTTTC
CTTGGTGTCACAGTTAGTGCATGCTATTATTGGTACTAGTCTTAAGACAGTTGCTTGGGCGATTGTTTCGATTTTT
GTAACTTTAGGTTTGATTGGACGTGAAATGATGCATTCAGTCATAACTGTAGTTAAGCGGTTATTAGAAAAATATC
ACTTGGCGACGCAACCCCAGGAATCCGCCAATTCAGGTACGGTTATTTCCGCTGTTCCAGAAGCACCCAATGCTGA
AGCAGAGGAGGCCAGTGCCTGGGTATCCATTATTTATAATGGTGTGTGTAATATGTTGAATGTAGCCGCTCAAAAA
CCGAAACAATTTAAAGATTGGGTAAAATTAGCTACCGTAGATTTTAGTAATAATTGTAGAGGTAGTAATCAGGTAT
TTGTGTTTTTCAAGAATACGTTTGAAGTGTTGAAGAAAATGTGGGGTTATGTGTTTTGTCAGAGTAATCCTGCAGC
GCGACTCTTGAAAGCAGTGAATGATGAACCTGAGATTTTAAAAGCGTGGGTTAAAGAATGTCTGTATTTAGATGAT
CCTAAATTTAGAATGCGACGTGCACATGATCAAGAGTATATTGAGAGAGTGTTTGCGGCCCATTCGTATGGACAAA
TTTTATTGCATGACTTAACGGCTGAAATGAATCAATCGCGTAATTTAAGTGTGTTTACGAGAGTGTATGATCAAAT
ATCTAAATTGAAGACGGATCTCATGGAAATGGGATCAAACCCATATATCAGGCGTGAATGTTTTACGATTTGTATG
TGTGGTGCATCTGGAATTGGTAAGTCTTATTTAACTGATTCTTTATGCAGCGAGCTCTTACGTGCGAGTCGTACTC

CAGTGACAACGGGCATTAAGTGTGTTGTGAACCCTTTGTCTGATTATTGGGATCAGTGTGATTTTCAGCCCGTTTT
ATGTGTTGATGACATGTGGAGTGTTGAAACGTCTACTACGCTCGATAAACAGTTAAATATGCTATTTCAGGTTCAT
TCACCAATTGTACTTTCACCTCCTAAAGCTGATTTAGAAGGTAAGAAAATGCGTTATAATCCTGAAATATTCATAT
ATAATACGAATAAACCTTTTCCGAGGTTTGATCGTATAGCTATGGAAGCTATTTATCGACGTAGAAACGTTTTAAT
TGAATGTAAGGCTAATGAAGAGAAGAAGCGTGGATGTAAACATTGTGAGAATAATATACCCATTGCTGAATGTAGT
CCAAAAATTTTGAAAGATTTTCATCACATTAAATTTCGTTATGCTCATGATGTGTGCAATTCTGAAACTACGTGGT
CTGAGTGGATGTCGTATAATGAATTTTTGGAATGGATTACTCCTGTATATATGGCTAATCGACGTAAAGCAAATGA
ATCGTTTAAGATGCGTGTTGATGAAATGCAAATGTTGCGTATAGACGAGCCCTTGGAAGGCGATAATATTTTAAAT
AAGTATGTTGAAGTTAATCAGCGCTTAGTTGAGGAAATGAAAGCTTTTAAAGAGCGAACCCTCTGGGCTGATTTAC
AACGTGTTGGCTCAGAGATTAGTACTTCAGTTAAGAAAGCATTACCAACTATTTCCATTACTGAGAAGCTACCACA
TTGGACCATCCAATGTGGCATAGCTAAGCCTGAAATGGATCATGCTTATGAAGTTATGAGCTCATATGCAGCAGGA
ATGAACGCAGAAATTGAAGCGCATGAACAAGTTCGTCGTTCTTCTTTGGAATGTCAGTATGTTGAGCCTTCAACTT
CAAGACCTCTGGATGAAGAGGGTCCTACTATCGACGAGGAATTACTTGGCGAAGTAGAATTTACTTCTTCAGCTTT
GGAGCGTTTGGTTGATGAGGGGTATATTACTGGTAAACAAAAGAAGTACATGGCAACTTGGTGTACGAAACGAAGA
GAGCATGTATCTGATTTTGATTTAGTATGGATGGATAATTTGCGTGTTTTAAGTGCGTATGTCCACGAGCGTTCTA
CATCTACGCGTTTGTCTACCGATGATGTTAAATTATTTAAGACGATTAGTATGTTACATCAGAGGTATGATACCAC
TGATTGTGCAAAATGCCAACATTGGTATGCACCATTAACAGCTATTTATGTTGATGATAGAAAGCTGTTTTGGTGC
CAGAAGGAGACTAAGACTTTGATAGATGTTCGTAAATTGTCGAAAGAGGACGTTACAGTCCAATCGAAATTAATTA
ACTTATCGGTTCCGTGCGGTGATGTATGTATATTACATTCTAAGTACTTTAATTATTTATTTCATAAAGCGTGGTT
GTTTGAAAATCCAACATGGCGTTTAATATATAACGGTACTAAGAAAGGTATGCCTGAGTATTTCATGAATTGCGTG
GATGAAATTTCATTAGATTCAAAATTTTGTAAAGTAAAGGTTTGGCTTCAAGCAATTATTGATAAATATTTGACTC
GTCCAGTGAAAATGATTCGTGACTTTCTATTTAAATGGTGGCCGCAAGTAGCATACGTGTTAAGTTTGTTAGGTAT
AATTGGTATAACTGCGTATGAGATGCGTAATCCTAAATCAACAGCAGAAGACTTGGCTGAGCACTATGTTAATAGG
CATTGTAGTTCAGATTTTTGGTCACCAGGTATGGCAACTCCTCAGGGATTAAAATATAGTGAAGCGATAACAGCTA
AAGCGCCTAGAATTCATAGATTGCCCGTTACTACTAGACCTCAGGGATCAACGCAACAAGTTGACGCCGCTGTGAA
TAAAATTTTGCAGAATATGGTGTATATCGGTGTTGTGTTTCCAAAAGTGCCTGGTAGTAAGTGGCGAGATATTAAT
TTTAGATGTCTTATGCTTCATAATCGGCAGTGTTTGATGTTGCGGCATTACATTGAGTCGACGGCTGCTTTTCCGG
AGGGTACCAAATACTATTTTAAGTATATTCATAATCAAGAAACTCGAATGTCAGGTGATATATCTGGTATTGAGAT
TGATTTATTGAGTTTACCTAGATTGTATTATGGTGGCTTAGCCGGGGAAGAGTCGTTCGATAGCAATATAGTGTTA
GTAACTATGCCGAATAGAATTCCTGAGTGTAAGAGTATTGTGAAGTTTATAGCTTCACATGCTGAACATGCTCGTG
CTCAAAATGATGGTGTGTTAGTTACTGGTGAACATACTCAGTTATTGGCGTTTGAGAATAATAATAAAACACCTAT
AAGTATTAATGCTGATGGTTTGTATGAGGTTATACTTCAAGGAGTATACACCTATCCATACCATGGTGATGGTGTT
TGTGGGTCTATATTATTGTCTCGTAATTTACAACGACCGATTATAGGGATCCATGTAGCTGGTACTGAAGGATTAC
ATGGCTTTGGTGTTGCTGAACCTCTTGTTCATGAGATGTTCACTGGGAAAGCAATAGAGAGTGAAAGGGAACCGTA
TGATCGTGTGTATGAATTACCTTTGCGTGAATTAGATGAATCTGATATAGGTTTAGATACTGATTTATATCCTATA
GGAAGAGTCGATGCGAAATTAGCTCATGCCCAAAGTCCTTCAACAGGAATTAAAAAGACGCTTATTCATGGTACTT
TTGATGTTCGGACTGAACCGAATCCGATGTCATCACGAGACCCAAGAATAGCGCCACATGATCCGTTGAAGTTGGG
GTGTGAGAAACATGGTATGCCATGTTCTCCATTTAATCGGAAACATTTGGAATTAGCAACAACTCATTTAAAGGAG
AAGTTAATTTCCGTAGTTAAACCTATAAATGGATGCAAGATTAGAAGTTTGCAAGATGCTGTATGTGGTGTACCAG
GTTTGGATGGCTTTGATTCAATATCCTGGAATACTAGTGCTGGTTTTCCTTTATCTTCATTAAAACCGCCAGGCTC
TTCTGGTAAGCGATGGTTGTTTGATATTGAATTACAAGATTCAGGATGTTATCTTTTGAGAGGGATGAGACCTGAA
CTTGAGATACAGTTGACAACAACTCAGTTAATGAGGAAGAAGGGAATAAAGCCTCACACTATATTCACAGATTGTT
TGAAAGATACATGTTTGCCTGTGGAAAAATGCAGAATACCTGGTAAAACTAGAATATTTAGTATAAGTCCCGTCCA
ATTTACGATTCCATTTCGACAATACTATCTCGATTTTATGGCGTCGTACCGTGCCGCTAGACTTAATGCTGAGCAT
GGAATAGGTATAGACGTGAACAGCTTGGAATGGACAAACTTGGCAACAAGTCTGTCGAAATATGGCACGCATATTG
TGACAGGAGATTATAAGAATTTTGGTCCTGGGTTAGATTCTGATGTTGCCGCTTCAGCTTTTGAAATTATCATTGA
TTGGGTGTTAAATTACACTGAAGAAGATGATAAAGACGAAATGAAGCGTGTAATGTGGACTATGGCTCAGGAAATC
TTAGCTCCTAGTCACTTATGTCGTGATTTAGTATATCGCGTGCCATGCGGTATTCCTTCTGGATCACCGATTACGG
ACATTTTGAATACTATTTCGAATTGTTTGTTAATTCGATTGGCTTGGCAAGGTATTACTGATTTGCCTTTATCCGA
ATTTTCTAGACATGTCGTGCTAGTTTGTTACGGTGATGATCTCATCATGAATGTAAGTGATGAGATGATAGATAAA
TTCAATGCTGTAACAATTGGCGATTTCTTTTCGCGATATAAGATGGAATTTACGGATCAGGATAAGTCTGGAAATA
CAGTGCGGTGGCGAACTTTACAAACTGCTACGTTTTTGAAGCATGGGTTCTTGAAACATCCAACAAGACCCGTGTT
TCTAGCCAATCTGGATAAGGTTTCTATAGAAGGAACAACCAATTGGACACATGCTCGAGGATTGGGTCGTCGAGTA
GCAACCATTGAGAATGCTAAACAAGCGCTAGAGTTGGCATTCGGATGGGGTCCCGAATACTTCAATCATGTTCGGA
ATACCATTAAAATGGCATTCGACAAGTTAGGTATTTATGAGGATCTCATAACGTGGGAAGAAATGGATGTTAGATG
TTATGCTAGTGCGTAATTTTAAGATTTTAATACTCATTAAAATTAATTCATATTTAGGTTATTGGAATTGAGGGAA
GTACCACCCCCCAAGACCTTCGTTTTAAATCTGCTAAGAGGAGTGAACTTGCATATAAGAGTCTAAAAGCAGAGTG
GATTAGACCACCACTTTTAGCTTATATGTGAGGAAGGTTGAGTTGCCTCTAAAGACTCAGCTCCGTAGTAGAGTAG

TTTTAGTTACGATTAAAGTGGTACTCTAGGTTAGGTGTTACTCGCGTATTGTCGCATAACGGCAATGCGTCCTAAT
TTTAGTATAGTTTAACCATAATAG

>VDV1-USA-FL-22-cons
TGGGAGGCGATTTATGCCTTCCATAGCGAATTACGGTGCAACTAACAATTTTAGATAGTAGCCATGAACAAACATT
ATGATTACTCACTACGTATTGATCATTTTTACAATGACTTGCGTAGTATGAAGCGCATGCTTGTAGTTATAACTAT
GTTATTTTGCAAGTTGGAGATAATTGTATTGGATTATGGATGCGTGCACTAAGTGTCTACATCTATAGTCGTTTGT
GGTTCAAGTTTTTGTGTTAGTAGTACAATCTTGAGGAATGTAAGTATCGTATGAATGATATTTGAATAACAACACT
GAAGTATAAAATATATAAAATCCAAAAATATTTTTAATCTTATTCAGTGTAGTGTTTGATAGAGTAGAATGCCATG
TGACCGCTCAAAGAAGTCCATTATGGTATATCATTCGAAGTCGAATACTTGTGTATAGTTATTGTATTTTATTAGT
AATATTAGTAGTCCGTAACTATCATAATCCTATTATAGTTTGATTATATGATAGACCACTGCAGTATCGAGTAGAG
TTTAGAAAGAGTAGTGCAATAGTAAGATCACTGTCACCGACCACTCATTGTAATAGTGAGGTTTGTCGGAAACCAG
TTATTGTGCAGCGACTAGCAATCGTGAATCAATATAGTTGGTATTCTAAATATGAGACGATTCGGCGATTTTATTG
CGACTGAAATTTCATATTTAGCATGTCAGGTCTTATTATGAATGCTCGAGTATTTATTTCTGCGGTAGAGTAGGGA
CCCCTCTATCTCTCAGGTACTGTATGAGGCGAAAGTGTGAAAGTAATTTATGTCTCTATACATAAGTGACTGTATT
GGGATTTCCTTTGGCAAGAATCCTTTTAATACAGTATAATTTATGCCACGGTACGTTACGTTCGCAGGGCACCCGT
TAATGTCACATAGCCCAGACGATGACGAATGGAAAGACATTACTTTTTATTTTAATGCTACGATTATTGCTGTTTT
ATTTTGCTGTTTTAATTTGCTATTATATTTTGCTATTTTATTATTGCTAAATATATTTCTTTGCTATTTTTGCTTT
ATATATTAGATTCAATTCTTTTTATTTTATATTTTCAATTTGATTTTGATTTTGAAGGTAAATATATATATAAAAATG
GCATTTAGTTGTGGAACTCTTTCTTATGCTGCTGTTGCCCAAGCTCCCTCTGTAGCTCATGCTCCCCGTAGTTGGG
AGATTGATGAAGCTAGGCGTCGACGCGTTATTAAGCGTTTGGCGTTGGAACAGGAACGGATTCGAAACGTTCTTGA
CGTCACTGTGTATGATCATACAACGTGGGAGCAAGAGGATGCGCGTGATAATGAGTTCCTTACGGAACAATTGAAT
AATTTATATATGATATATTCTATAGCTGAAAGATGTACCCGTCGCCCTGTTCAAGAACATGTCCCCATTTCAATCA
GTAATAGATATTCCCCTTTAGAATCCCTTAAGATTGAGGTAGGAAAAGACGCAGGTGAGTTCGTATTTAAGAAACC
CAAATATACAAAGATTTGTAAGAAAGTGAAACGGGTGACATCAAAATTTGTGCGCGAGAAAGTTGTTAGGCCCGTT
TGTAATCGATCGCCCATGTTATTATTTAAAATTAAGAAAGTAATATATGATTTACATTTGTATCGGTTACGGAAAC
AAGTTCGGCTTCTCAGACGCGAAAAACAGCGTGAATACGAGTTAGAGTGTGTTACTAGTTTGCTACAGCTATCTAA
TCCTGTTTCAGCTAAACCTGAGATGGACAATCCTAATCCTGGTCCAGATGGTGAAGGTGAAGTTGAATTAGAAAAG
GATAGTAATGTAGTATTAACTACACAACGTGATCCTAGTACCTCTATTCCTGCTCCAACTAGTGTGAAGTGGAGTA
GATGGACTAGTAATGATGTTGTGGATGATTATGCCACTATAACTTCGCGTTGGTATCAAATTGCCGAATTTGTATG
GTCAAAGGATGATCCATTTGATAAGGAATTGGCGCGCTTAATTTTACCTCGAGCTTTGTTATCTAGTATTGAGGCT
AATTCTGACGCTATTTGTGATGTACCTAATACTATTCCGTTTAAGGTACATGCATATTGGCGTGGAGATATGGAAG
TTCGAGTGCAGATTAACTCGAATAAATTCCAGGTTGGTCAATTACAGGCAACTTGGTACTATTCGGATCATGAAAA
TCTGAATATCCAGACGAAGCGAAGTGTGTATGGTTTTTCGCATATGGATCATGCTCTGATTAGCGCATCAGCGAGT
AATGAAGCAAAATTAGTGATACCTTTTAAACACGCATATCCATTCTTACCAACGCGTGTCGTTCCTGATTGGACAA
CTGGTATTCTTGATATGGGTACCTTAAATATTCGTGTAATTGCTCCACTACGTATGAGTGCGACGGGACCAACCAC
TTGTAATGTTGTAGTATTTATTAAGTTAAATAATAGTGAATTCACTGGTACTTCTTCTGGTAAGTTTTACGCGAAT
CAAATTAGGGCAAAACCTGAAATGGACCGTGTGTTAAATTTGGCAGAAGGATTACTAAATAATACTGTAGGTGGTT
GTAATATGGATAATCCGTCATATCAGCAATCTCCGCGTCATTTTGTTCCTACTGGTATGCATAGTTTAGCTTTAGG
CACTAATTTAGTAGAGCCTTTGCATGCATTACGATTAGATGCATCAGGTACAACACAACATCCAGTTGGGTGTGCG
CCTGATGAAGATATGACTGTATCTTCCATTGCATCACGATATGGTTTAATTCGCCAAGTGCAATGGAAGAAAGACC
ATGCGAAAGGATCATTATTATTACAACTTGATGCTGATCCTTTCGTTGAACAGAAAATTGAGGGAACCAATCCAAT
TTCTTTGTATTGGTTTGCTCCGGTTGGAGTCGTATCTAGTATGTTTATGCAATGGAGAGGTTCTTTAGAATATAGA
TTTGATATTATAGCTTCCCAATTTCATACGGGTAGGTTAATTGTAGGTTATGTTCCTGGACTGACTGCTTCTTTAC
AACGTCAAATGGACTATATGAAATTGAAGTCATCTAGTTATGTGGTGTTTGATTTACAGGAAAGTAATAGTTTTAC
GTTTGAAGTGCCCTATGTGTCATACAGACCGTGGTGGGTGCGTAAGTATGGTGGTAATTATCTGCCATCTTCTACT
GATGCGCCTAGCACACTGTTTATGTATGTACAAGTACCATTGATACCTATGGAAGCTGTTTCTGATACTATAGATA
TCAATGTGTATGTGCGTGGTGGCAGTTCGTTTGAGGTTTGTGTTCCAGTCCAACCTAGTTTAGGTTTGAACTGGAA
TACAGATTTCATATTACGTAATGATGAGGAGTACCGCGCAAAGAATGGATATGCACCATATTATGCTGGTGTGTGG
CATAGCTTCAATAATAGTAATTCGCTTGTTTTTAGATGGGGTTCGGCTTCAGATCAAATTGCTCAATGGCCAACAA
TAACAGTGCCTCGAGGAGAGTTGGCATTCCTGCGTATCCGCGATGCTAAGCAAGCTGCTGTAGGAACGCAACCTTG
GCGTACTATGGTCGTTTGGCCTTCAGGTCATGGATATAATATTGGAATACCAACTTATAATGCTGAACGAGCAAGA
CAACTTGCTCAGCATTTGTATGGTGGTGGGTCTTTGACAGATGAAAAGGCTAAGCAATTATTTGTGCCTGCTAACC
AGCAAGGACCCGGCAAAGTAAGTAATGGTAACCCCGTCTGGGAAGTAATGCGCGCGCCTCTTGCAACTCAGCAAGC
GCATATACAAGATTTTGAATTTGTTGAAGCTGTTCCAGAAGGCGAAGAATCACGCAACACTACGGTGCTAGACACA
ACAACAACGTTACAGTCTAGCGGATTTGGTCGCGCTTTCTTCGGTGAGGCATTTAACGATCTTAAGACGTTAATGC
GCCGATACCAATTGTATGGTCAATTATTGTTATCCGTTACTACGGATAAGGATATTGATCATTGTATGTTTACCTT
CCCTTGTTTACCTCAAGGGTTAGCGTTAGATATAGGTTCGGCTGGATCGCCTCATGAAATATTTAATCGCTGCCGT

GATGGTATCATTCCGTTGATAGCGTCAGGGTATCGGTTTTATCGAGGCGATTTACGGTTCAAAATTGTTTTCCCAA
GTAACGTTAATAGCAATATTTGGGTACAACATCGACCAGATCGTAGACTGAAAGGATGGTCTGAAGCGAAAATAGT
AAACTGTGATGCTGTATCTACTGGACAAGGTGTTTATAATCATGGATATGCTAGTCATATTCAGATTACGCGTGTA
AATAATGTTATAGAATTGGAAGTCCCGTTTTATAACGCTACGTGCTATAATTATTTGCAAGCGTTTAACCCATCTA
GTGCAGCGTCGAGTTATGCCGTTTCGCTCGGAGAGATTTCGGTTGGTTTTCAAGCTACTAGTGATGACATTGCAGC
CATAGTTAATAAACCTGTAACTATATATTACAGTATTGGCGATGGTATGCAGTTTTCGCAGTGGGTTGGTTATCAA
CCAATGATGATTCTAGACCAATTGCCAGCACCAGTAGTTAGGGCTGTGCCTGAGGGCCCTATAGCGAAGATAAAGA
ACTTTTTCCACCAAACGGCAGATGAAGTTCGAGAAGCTCAGGCCGCAAAGATGCGTGAAGATATGGGTATAGTAGT
CCAAGACGTTATAGGAGAGTTAAGTCAGGCTATACCCGATCTTCAACAACCGGAAGTTCAAGCGAATGTTTTTTCT
CTGGTGTCACAGTTAGTGCATGCTATCATCGGTACTAGTCTTAAGACAGTTGCTTGGGCGATTGTTTCGATTTTTG
TAACTTTAGGTTTGATTGGACGTGAAATGATGCATTCAGTCATAACTGTAGTTAAGCGGTTATTAGAAAAATATCA
CTTGGCGACGCAACCCCAGGAATCCGCCAATTCAGGTACGGTTATTTCCGCTATTCCAGAAGCACCCAATGCTGAA
GCAGAGGAGGCCAGTGCCTGGGTATCCATTATTTATAATGGTGTGTGTAATATGTTGAATGTAGCCGCTCAAAAAC
CGAAACAATTTAAAGATTGGGTAAAATTAGCTACCGTAGATTTTAGTAATAATTGTAGAGGTAGTAATCAGGTATT
TGTGTTTTTCAAGAATACGTTTGAAGTGTTGAAGAAAATGTGGGGTTATGTGTTTTGTCAGAGTAATCCTGCAGCG
CGACTCTTGAAAGCAGTGAATGACGAACCTGAGATTTTAAAAGCGTGGGTTAAAGAATGTCTGTATTTAGATGATC
CTAAATTTAGAATGCGACGTGCGCATGATCAAGAGTATATTGAGAGAGTGTTTGCGGCCCATTCGTATGGACAAAT
TTTATTGCATGACTTAACGGCTGAAATGAATCAATCGCGTAATTTAAGTGTGTTTACGAGAGTGTATGATCAAATA
TCTAAATTGAAGACGGATCTCATGGAAATGGGATCAAACCCATATATCAGGCGTGAATGCTTTACGATTTGTATGT
GTGGTGCATCTGGAATTGGTAAGTCTTATTTAACTGATTCTTTATGCAGCGAGCTCTTACGTGCGAGTCGTACTCC
AGTGACAACGGGCATTAAGTGTGTCGTGAACCCTTTGTCTGATTATTGGGATCAGTGTGATTTTCAGCCCGTTTTA
TGTGTTGATGACATGTGGAGTGTTGAAACGTCTACTACGCTCGATAAACAGTTAAATATGTTATTTCAGGTTCATT
CACCAATTGTACTTTCACCGCCTAAAGCTGATTTAGAAGGTAAGAAAATGCGTTATAATCCTGAAATATTCATATA
TAATACGAATAAACCTTTTCCGAGGTTTGATCGTATAGCTATGGAAGCTATTTATCGACGTAGAAACGTTTTAATT
GAATGTAAGGCTAATGAAGAGAAGAAGCGTGGATGTAAACATTGTGAGAATAATATACCCATTGCTGAATGTAGTC
CAAAAATTTTGAAAGATTTTCATCACATTAAATTTCGTTATGCTCATGATGTGTGTAATTCTGAAACTACGTGGTC
TGAGTGGATGTCGTATAATGACTTTTTGGAATGGATTACTCCTGTATATATGGCTAATCGACGTAAAGCAAATGAA
TCGTTTAAGATGCGTGTTGATGAAATGCAAATGTTGCGTATGGATGAGCCCTTGGAAGGCGATAATATTTTAAATA
AGTATGTTGAAGTTAATCAGCGCTTAGTTGAGGAAATGAAAGCTTTTAAAGAGCGAACCCTCTGGGCTGATTTACA
ACGTGTTGGCTCAGAGATTAGTACTTCAGTTAAGAAAGCATTACCAACTATTTCCATTACTGAGAGGCTACCACAT
TGGACTATCCAATGTGGCATAGCTAAGCCTGAAATGGATCATGCTTATGAAGTTATGAGTTCATATGCAGCAGGAA
TGAACGCAGAAATTGAAGCGCATGAACAAGTTCGTCGTTCTTCTTTGGAATGTCAGTATATTGAGCCTTCAACTTC
AAGACCTCTGGATGAAGAGGGTCCTACTATCGACGAGGAATTACTTGGCGAAGTAGAATTTACTTCTTCAGCTTTG
GAGCGTTTGGTTGATGAGGGGTATATTACTGGTAAACAAAAGAAGTATATGGCAACTTGGTGTACGAAACGAAGAG
AGCATGTATCCGATTTTGATTTAGTATGGACGGATAATCTGCGTGTTTTGAGTGCGTATGTCCACGAGCGTTCTAC
ATCTACGCGTTTATCTACCGATGATGTTAAATTATTTAAGACGATTAGTATGTTACATCAGAGGTATGATACCACT
GATTGTGCAAAATGCCAACATTGGTATGCACCATTAACAGCTATTTATGTTGATGATAGAAAGCTATTTTGGTGCC
AGAAGGAGACTAAGACTTTGATAGATGTTCGTAAATTGTCGAAAGAGGATGTTACAGTCCAATCGAAATTAATTAA
CTTATCGGTTCCGTGCGGTGATGTGTGTATGTTACATTCTAAGTACTTTAATTATTTATTCCATAAAGCGTGGTTG
TTTGAAAATCCAACATGGCGTTTAATATATAATGGTACTAAGAAAGGTATGCCTGAGTATTTCATGAATTGCGTGG
ATGAAATTTCATTAGATTCAAAATTTTGTAAAGTAAAGGTTTGGCTTCAAGCAATTATTGATAAATATTTGACTCG
TCCAGCGAAAATGATTCGTGACTTTCTATTTAAATGGTGGCCGCAAGTAGCATACGTGTTAAGTTTGTTAGGTATA
ATTGGTATAACTGCGTATGAAATGCGTAATCCTAAATCAACAGCAGAAGACTTGGCTGAGCACTATGTTAATAGGC
ATTGTAGTTCAGATTTTTGGTCACCAGGCATGGCGACTCCTCAGGGATTAAAATATAGTGAAGCGATAACAGCTAA
AGCGCCTAGAATCCATAGATTGCCCGTTACTACTAGACCTCAGGGATCAACGCAACAAGTTGACGCCGCTGTGAAT
AAGATTTTGCAGAATATGGTGTATATCGGTGTTGTGTTTCCAAAAGTGCCTGGTAGTAAGTGGCGAGATATTAATT
TTAGATGTCTTATGCTTCATAATCGGCAATGTTTGATGTTGCGGCATTACATTGAGTCGACGGCTGCTTTTCCGGA
GGGTACCAAATACTATTTAAGTATATTCATAATCAAGAAACTCGAATGTCAGGTGATATATCTGGTATTGAGATT
GATTTATTGAGTTTACCTAGATTATATTATGGTGGCTTAGCTGGGGAAGAGTCGTTCGATAGTAATATAGTGTTAG
TAACTATGCCGAATAGAATTCCTGAGTGTAAGAGTATTGTGAAGTTTATAGCTTCACATGCTGAACATGCTCGTGC
TCAAAATGATGGTGTGTTAGTTACTGGTGAACATACTCAGTTATTGGCGTTCGAGAATAATAATAAAACACCTATA
AGTATTAATGCTGATGGTTTGTATGAGGTTATACTTCAAGGAGTATACACTTATCCATACCATGGTGATGGTGTTT
GTGGGTCTATATTATTGTCTCGTAATTTACAACGACCGATTATAGGGATCCATGTAGCTGGTACTGAAGGATTACA
TGGCTTTGGTGTTGCTGAACCTCTTGTTCATGAGATGTTCACTGGGAAAGCAATAGAGAGTGAAAGGGAACCGTAT
GATCGTGTGTATGAATTACCTTTGCGTGAATTAGATGAATCTGATATAGGTTTAGATACTGATTTATATCCTATAG
GAAGAGTTGATGCGAAATTAGCTCATGCCCAAAGTCCTTCAACAGGAATTAAAAAGACGCTTATTCATGGTACTTT
TGATGTTCGGACTGAACCGAACCCGATGTCATCACGAGACCCAAGAATAGCGCCACATGATCCGTTGAAGTTAGGG
TGTGAGAAACATGGTATGCCATGTTCTCCATTTAATCGAAAACATTTGGAATTAGCAACAACTCATTTAAAGGAGA
AGTTAATTTCCGTAGTTAAACCTATAAACGGATGCAAGATTAGAAGTTTGCAAGATGCTGTGTGTGGTGTACCAGG

TTTGGATGGCTTTGATTCAATATCCTGGAATACTAGTGCTGGTTTTCCTTTATCTTCATTAAAACCGCCAGGCTCT
TCTGGTAAGCGATGGTTGTTTGATATTGAATTACAAGATTCTGGATGTTATCTTTTGAGAGGGATGAGACCTGAAC
TTGAGATACAGTTGACAACAACTCAGTTAATGAGGAAGAAGGGAATAAAGCCTCACACTATATTCACGGATTGTTT
GAAAGATACATGTTTGCCTGTGGAAAAAATGCAGAATACCTGGTAAGACTAGAATATTTAGTATAAGTCCCGTCCAA
TTTACGATTCCATTCCGACAATACTATCTCGATTTTATGGCGTCGTACCGTGCCGCTAGACTTAATGCTGAGCATG
GAATAGGTATAGACGTGAACAGCTTGGAATGGACAAACTTGGCAACAAGTCTGTCGAAGTATGGCACGCATATTGT
GACAGGAGATTACAAGAATTTTGGTCCTGGGTTAGATTCTGATGTTGCCGCTTCAGCTTTCGAAATTATCATTGAT
TGGGTGTTAAATTACACTGAAGAAGATGATAAAGACGAAATGAAGCGTGTAATGTGGACTATGGCTCAGGAAATCT
TAGCTCCTAGTCACTTATGTCGTGATTTAGTATATCGCGTACCATGCGGTATTCCTTCTGGATCACCAATTACGGA
CATTTTGAATACTATTTCGAATTGTTTGTTAATTCGATTGGCTTGGCAAGGTATTACTGATTTGCCTTTATCCGAA
TTTTCTAGACATGTCGTGCTAGTTTGTTATGGTGATGATCTCATCATGAATGTAAGTGATGAGATGATAGATAAAT
TCAACGCTGTAACAATTGGCGATTTCTTTTCGCGATATAAGATGGAATTTACGGATCAGGATAAATCTGGAAATAC
AGTGCGGTGGCGAACTTTACAAACTGCCACGTTTTTGAAGCATGGGTTCTTAAAACATCCAACAAGACCCGTGTTT
CTAGCCAATCTGGATAAGGTTTCTATAGAAGGAACAACCAATTGGACACATGCTCGAGGATTGGGTCGTCGAGTAG
CAACCATTGAGAATGCTAAACAAGCGCTAGAGTTGGCATTCGGATGGGGTCCCGAATACTTTAATCATGTTCGGAA
TACCATTAAAATGGCATTCGACAAGTTAGGTATTTATGAGGATCTCATCACATGGGAAGAAATGGATGTTAGATGT
TATGCTAGCGCGTAAATTTAAGATTTTAATACTCATTAAAATTAATTTATATTTAGGTTATTGGAATTGAGGGAAG
TACCACCCCCCAAGACCTTCGTTTTAAATCTACTAAGAGGAGTGAACTTGCATATAAGAGTCTAAAAGCAGAGTGG
ATTAGACCACCACTTTTAGCTTATATGTGAGGAAGGTTGAGTTGCCTCTAAAGACTCAGCTCCGTAGTAGAGTAGT
TTTAGTTACGATTAAAGTGGTACTCTAGGTTAGGTGTTACTCGCGTATTGTCGCATAACGGCAATGCGTCCTAATT
TTAGTATAGTTTAACCATAATA

>DWV-USA-MD-2-cons
TGGGAGGCGATTTATGCCTTCCATAGCGAATTACGGTGCAACTAACAATTTTAGATAGTAGCCATGAACAAACATT
ATAGTGGCTCACTACGTATTGATCATTTTTATAATGACTTGCGTAGCATGAAGCGCATGCTTGTAGTTGTAACTAT
GTTACTTTGCAAGTTGGAGTTTACTATTTTGGATTATGAATATGTGCACTTAGTGTCTGTATTTATAGTCGTTTGT
GGTTCAAGGTTTTGTGTTAGTAGTACACTTATGTATGAATGTACCTTTAGTATGAATGTTATAGAATGACAATATC
GAAAGAAAAATCTTTATAAAATACAAAAATATTGTTTTTATTATTTCGATATGGTGTTTTATAGAGTAGATTGCCA
TGTGACCGCTCGTAGAAGTCCATTATGGTTTATCAATCGAAGTTGAATGTATTTATAAGAATATTATACTTAATTA
GTAATATTAGTAGTCCGTAACTATTATCATCCTTTTTCAGTTTGATGTGATAATAGACCACTGCAGTATCGAGTAG
AGTTTCGAATGCGTAGTGCAATAGTACAATCACTGTCACCGACCATCTATTGTAATGATAGATCTGTCGGAAACCA
TTATTTATGAAGTGACTAGCAATCATGGATTAAATTAGATGGTATTCTAGTTTAGAGGTGATTCGGCGCTGCGGTG
CGACTGAAACTTCTAAATTAGCATGTCAGATTGTATTATGAATGCGTTAGTAGTAATTTCTGCGATAGAGCTGGGA
CCCCTCAGTCTCTCAGGTATTGTACGAGGCGAAAGTGTGAAAGTTTTGTATGTGTTTTTATATGTACGACTGTATC
GGGAATTCCTTTAGCAAGAATCCTTTTAATACAGTATAATCTGTGCTACGGTACGTTACGTTCGCAGGGCACCCGT
TAATGTCTCATAGCCCAGACGATGGCGGATGGAAAGACATCATATTTTATTTTAATGCTGTCTTTATTGCTGATTT
ATCTTGCTGTTTTTATTTGCTATTTTATATTTGCTAATTTTCATTATTGCGAAATATATTACATTGCTATTTTTAT
TATATACGCTAGATTCAATTTTATTTTTCCTATATTTTCAATTTAATTTTGATTTTGAAGGTAAATATATATAATT
AATTATTAAAAATGGCTTTTAGTTGTGGAACTCTTTCTTACTCTGCCGTCGCCCAAGCTCCGTCTGTTGCCTATGC
ACCTCGTACATGGGAAGCTGATGAAGCTAGGCGGCGCCGAGTCATTAAACGTTTGGCGCTGGAGCAAGAACGTATG
CGTAACGTTCTTGACGTTGGCGTCTATGACCAGGCGACATGGGAACAGGAGGACGCGCGCGATAATGAGTTCCTAA
CGGAACAATTAAACAATTTATATACTATTTATTCGATCGCTGAACGTTGTACGCGTCGACCTATTAAAGAGCACTC
TCCTATATCAGTTTCGAATAGGTTTGCTCCATTGGAATCCCTCAAGGTCGAGGTCGGTCAAGAAGCAGGCGAATGT
ATATTTAAGAAACCTAAATATACGCGCGTTTGCAAGAAAGTGAAGCGTGTTGCAACTCGCTTCGTTCGTGAAAAAG
TTGTTCGTCCTATGTGTTCTAGATCTCCTATGCTATTATTTAAGCTTAAGAAAATTATTTATGATTTGCACTTATA
TAGATTAAGAAAACAGATTAGGATGTTGAGACGTCAAAAACAGCGCGAGTATGAGTTAGAGTGTGTCACTAATCTG
TTACAATTATCGAATCCGGTGCAGGCAAAACCAGAGATGGATAACCCTAATCCAGGACCTGATGGCGAGGGTGAAG
TTGAATTAGAAAAGGATAGTAATGTTGTTTTAACAACTCAGCGAGATCCTAGTACATCTATTCCAGCGCCGGTGAG
CGTAAAATGGAGTAGATGGACTAGTAATGATGTAGTAGATGATTACGCCACAATCACATCTCGATGGTATCAGATT
GCTGAATTTGTTTGGTCGAAGGATGATCCATTTGATAAGGAGTTAGCACGTTTAATTTTGCCTCGTGCTTTGTTAT
CTAGTATAGAGGCTAATTCTGATGCTATATGTGATGTGCCTAATACTATTCCATTTAAGGTACACGCATATTGGCG
AGGCGATATGGAAGTTAGAGTTCAAATTAATTCAAATAAATTCCAAGTTGGTCAATTACAAGCTACTTGGTATTAT
TCGGATCATGAGAATTTGAATATATCGTCTAAGAGAAGCGTTTATGGATTTTCACAAATGGATCATGCTTTGATTA
GTGCGTCAGCAAGTAATGAAGCAAAATTAGTTATTCCATATAAGCATGTTTATCCATTTTTACCAACAAGAATTGT
GCCAGATTGGACTACTGGCATTTTAGATATGGGTGCTTTGAACATTCGTGTAATTGCTCCCTTACGGATGAGTGCT
ACTGGTCCAACTACCTGTAATGTCGTCGTGTTTATTAAATTAAATAACAGCGAGTTTACAGGGACTTCCTCTGGTA
AGTTTTATGCGAGCCAAATCAGGGCAAAACCTGAGATGGATCGTATATTAAATTTAGCAGAAGGATTGTTGAATAA
CACGATTGGTGGTAATAATATGGATAATCCTTCTTATCAACAATCTCCTCGTCATTTTGTCCCGACTGGTATGCAC

AGCTTAGCTTTAGGTACTAATTTAGTTGAACCATTACATGCATTACGTTTGGATGCAGCCGGTACGACACAACATC
CTGTAGGTTGTGCTCCTGATGAAGATATGACTGTGTCTTCCATTGCATCTCGATATGGACTAATTAGACGGGTACA
ATGGAAGAAAGATCATGCTAAAGGATCGCTTTTGTTACAATTAGATGCTGATCCATTTGTGGAGCAAAGAATTGAA
GGTACGAATCCAATATCTTTGTATTGGTTCGCACCCGTGGGTGTAGTATCTAGTATGTTTATGCAATGGCGTGGTT
CATTAGAATATAGGTTTGATATTATAGCATCCCAATTTCATACTGGTAGGTTAATTGTAGGTTATGTGCCCGGTTT
GACAGCATCTTTGCAACTTCAAATGGACTATATGAAATTGAAGTCATCGAGTTATGTAGTATTTGATTTACAGGAA
AGTAATAGCTTCACTTTTGAGGTGCCATATGTTTCATATAGACCATGGTGGGTGCGTAAATATGGTGGCAATTATT
TGCCCTCGTCAACTGATGCTCCTAGTACATTATTCATGTATGTGCAGGTTCCGTTGATACCTATGGAAGCTGTTTC
AGATACTATTGATATCAATGTGTACGTACGGGGCGGTAGTTCATTTGAAGTTTGTGTTCCAGTCCAACCTAGTTTA
GGTTTGAATTGGAACACAGACTTTATTTTACGTAATGACGAAGAATACAGGGCTAAGACAGGTTATGCACCATATT
ATGCTGGAGTGTGGCATAGCTTTAATAATAGTAATTCTCTTGTTTTTAGGTGGGATCTGCTTCTGATCAAATTGC
TCAGTGGCCGACAATTTCAGTACCAAGAGGTGAGTTAGCTTTCTTACGAATTAAGGATGGAAAGCAAGCTGCTGTA
GGAACTCAACCTTGGCGTACGATGGTTGTTTGGCCTTCTGGTCACGGCTATAATATTGGTATACCCACGTATAATG
CTGAACGAGCTCGCCAGCTTGCACAACACTTATATGGTGGTGGATCATTAACTGATGAGAAGGCCAAACAATTATT
TGTTCCTGCTAATCAACAAGGACCTGGTAAGGTAAGTAATGGAAATCCGGTATGGGAAGTCATGCGTGCACCATTG
TCAACACAGCGTGCGCATATGCAAGATTTTGAATTTATTGAGGCTATTCCAGAAGGAGAGGAGTCTCGTAATACTA
CAGTCTTGGATACGACCACTACTTTACAGTCGAGTGGGTTTGGTCGCGCCTTCTTTGGAGAAGCTTTTAATGATCT
TAAAACGTTAATGCGGCGATATCAATTATATGGTCAATTATTATTGTCCGTTACTACGGATAAGGATATTGATCAT
TGTATGTTTACCTTCCCTTGTTTACCACAAGGGTTAGCGTTAGACATTGGTTCTGCTGGCTCTCCACATGAAATCT
TTAATAGATGTCGTGATGGTATTATACCATTAATTGCATCTGGATATAGATTTTATAGAGGAGATTTGCGTTATAA
GATTGTTTTTCCAAGTAATGTTAATAGCAACATTTGGGTACAACATCGACCGGATCGTAGACTGGAAGGATGGTCC
GCGGCTAAGATTGTAAATTGTGATGCTGTGTCTACTGGTCAAGGAGTGTATAATCATGGTTATGCTAGTCACATTC
AAATCACGCGTGTAAATAATGTTATAGAATTGGAAGTTCCATTTTATAATGCTACTTGTTATAATTATTTACAGGC
GTTTAATGCGTCTAGCGCTGCATCTAGTTATGCAGTATCTTTAGGAGAAATATCGGTTGGTTTTCAAGCCACAAGT
GATGATATCGCATCTATCGTCAACAAACCTGTTACTATTTATTATAGTATTGGAGATGGTATGCAGTTTTCTCAGT
GGGTTGGATATCAACCGATGATGATCCTAGATCAGCTTCCTGCACCAGTAGTAAGGGCCGTGCCTGAGGGCCCTAT
TGCGAAGATTAAAAACTTCTTCCATCAAACAGCCGACGAAGTTAGAGAAGCTCAGGCAGCAAAGATGCGTGAAGAT
ATGGGTATGGTTGTTCAAGATGTTATTGGAGAACTTAGCCAGGCCATACCGGATCTTCAACAACCGGAGGTTCAAG
CAAATGTCTTCTCACTGGTGTCTCAGTTAGTGCATGCTATTATAGGTACTAGTTTGAAGACAGTAGCTTGGGCGAT
TGTTTCGATTTTTGTGACTTTAGGATTAATTGGACGCGAAATGATGCATTCAGTTATTACTGTAGTCAAGCGGTTA
CTTGAGAAATATCACTTGGCGACGCAACCCCAGGAATCCGCCAGTTCAAGTACTGTTATATCTGCCGTTCCAGAAG
CTCCCAATGCTGAAGCGGAGGAGGCAAGTGCTTGGGTATCCATTATTTATAATGGTGTGTGTAATATGCTTAATGT
GGCTGCTCAAAAACCGAAACAATTTAAAGATTGGGTAAAATTAGCTACTGTAGATTTTAGTAATAATTGTAGAGGT
AGTAATCAGGTATTTGTGTTTTTCAAGAATACATTTGAAGTGTTGAAGAAAATGTGGGGTTATGTATTTTGTCAGA
GTAATCCTGCAGCGCGTTTGTTGAAAGCTGTGAATGACGAGCCTGAGATTTTGAAAGCATGGGTGAAGGAATGTCT
GTATTTGGATGATCCCAAATTTAGAATGCGTCGAGCGCATGATCAAGAGTATATCGAGCGAGTGTTTGCGGCACAT
TCATATGGACAAATTTTGCTACATGATTTAACTGCTGAAATGAACCAATCACGAAATTTGAGTGTGTTTACACGTG
TGTATGATCAAATTTCAAAATTGAAGACTGATCTTATGGAAATGGGATCGAATCCATATATAAGGCGTGAATGTTT
TACGATATGCATGTGTGGTGCATCTGGAATTGGAAAATCATATTTGACCGATTCTTTATGCAGCGAGCTCTTACGT
GCGAGTCGTACTCCTGTGACAACAGGCATAAAATGTGTTGTTAATCCATTATCTGATTATTGGGATCAGTGTGATT
TTCAGCCTGTTTTGTGCGTTGATGATATGTGGAGTGTTGAAACATCTACTACGCTCGATAAGCAGTTGAACATGCT
TTTCCAGGTCCATTCCCCTATCGTGCTTTCTCCTCCTAAAGCTGATTTAGAAGGTAAGAAAATGCGATATAACCCG
GAAATATTTATATACAATACGAATAAGCCTTTCCCGAGGTTTGATCGTATTGCTATGGAAGCTATTTATCGGCGTA
GAAATGTTTTGATTGAATGTAAAGCGAGTGAAGAGAAGAAGCGAGGATGCAAACATTGTGAGAACGATATTCCTAT
TGCTGAATGTAGTCCTAAGATGTTGAAAGATTTCCATCACATCAAATTTAGGTATGCACATGATGTATGTAATTCC
GAGACCACATGGTCTGAATGGATGACGTATAATGAATTTCTTGAATGGATAACTCCTGTGTATATGGCTAACCGTC
GTAAGGCGAATGAATCGTTTAAGATGCGTGTAGATGAAATGCAAATGTTACGTATGGATGAGCCATTGGAAGGTGA
TAATATCCTCAATAAGTATGTTGAAGTTAATCAGCGCTTAGTGGAGGAAATGAAGGCATTTAAGGAGCGTACACTA
TGGTCAGATTTACATCGTTTAGGTGCGGAAGTTAGTGCGTCAGTTAAGAAAGCTTTACCAACTATTTCTATAACTG
AAAAACTACCACATTGGACTGTTCAATGTGGTATTGCTAAACCTGAGATGGATCATGCTTATGAGGTTATGAGTTC
GTATGCAGCTGGAATGAATGCAGAGATCGAAGCGCATGAACAAGTTCGGCGTTCATCAGTGGAATGTCAATATGCA
GAGCCTCAAGCTCCAAGAAATCCTGATGATGAAGGACCAACCATAGATGAAGAACTTATGGGCGACACTGAATTCA
CATCACAGGCTCTAGAACGTCTTGTGGATGAAGGTTATATAACTGGAAAACAGAAGAAGTATATAGCTACGTGGTG
TAGTAAGCGTCGTGAACATACTGCTGACTTTGATCTTGTGTGGACTGATAATTTGCGTGTGTTAAGCGCGTATGTG
CATGAACGTTCATCTTCAACTCGGCTTTCTACGGATGACGTCAAGTTATATAAAACGATTAGCATGTTACATCAAA
AGTACGATACCACAGAGTGTGCTAAATGTCAACATTGGTATGCACCGTTGACTGATATCTATGTTGATGACAAGAA
ATTGTTCTGGTGTCAGAAAGAGAAAAAGACACTTATTGATGTCCGAAAATTGTCGAAAGAAGATGTGACTGTTCAA
TCGAAATTGATTAATTTATCTGTTCCTTGTGGTGAAGTATGTATGTTACATTCAAAATATTTTAATTATCTTTTCC
ATAAAGCATGGTTGTTCGAGAATCCAACTTGGCGCCTAATATATAATGGTACCAAGAAGGGTATGCCTGAGTACTT

TATGAATTGTGTGGATGAAATATCGTTAGATTCCAAATTTGGTAAAGTGAAAGTATGGTTGCAAGCAATCATTGAT
AAGTATTTAACTCGTCCCGTGAAAATGATTCGTGATTTTCTTTTCAAGTGGTGGCCGCAAGTTGCGTATGTGTTGA
GCTTGCTAGGTATAATTGGTATAACTGCGTATGAAATGAGAAATCCGAAACCAACTTCTGAGGAATTAGCTGATCA
TTATGTGAATAGGCATTGTAGCTCTGATTTTTGGTCACCAGGACTGGCATCACCTCAAGGATTGAAATATAGTGAA
GCAGTAACAGTAAAGGCACCTAGAATCCATAGATTGCCAGTTACTACTAAGCCTCAGGGATCAACACAACAAGTAG
ACGCCGCTGTGAATAAAATTCTACAGAACATGGTTTATATTGGTGTTGTTTTCCCAAAAGTGCCTGGTAGTAAGTG
GCGAGATATTAATTTTAGGTGTCTTATGCTTCATAATAGGCAATGTTTAATGTTGAGGCATTATATTGAGTCAACT
GCCGCCTTTCCTGAGGGAACCAAGTACTATTTTAAGTACATTCATAATCAAGAGACTAGAATGTCTGGTGATATTT
CTGGTATTGAAATTGATTTGTTGAATTTACCTAGATTGTATTATGGTGGTCTCGCGGGAGAGGAGTCGTTTGATAG
CAATATCGTGCTTGTGACTATGCCTAATCGTATTCCTGAGTGTAAGAGCATTATTAAATTTATAGCGTCACATAAT
GAGCATATACGTGCTCAGAATGATGGAGTGTTAGTAACTGGCGACCATACTCAGCTATTGGCTTTCGAGAATAATA
ATAAAACTCCAATAAGTATTAACGCTGATGGTTTGTATGAGGTTATACTTCAAGGAGTATATACTTATCCATACCA
CGGCGATGGTGTTTGTGGTTCGATATTGTTATCTCGGAATTTACAACGGCCAATTATAGGTATCCATGTTGCTGGT
ACTGAGGGATTGCATGGTTTTGGAGTCGCTGAACCACTTGTACATGAAATGTTCACCGGTAAAGCGATCGAGAGTG
AAAGAGAGCCGTATGATCGTGTGTATGAACTTCCGTTGCGTGAATTAGATGAATCTGATATTGGTTTAGATACCGA
TTTATATCCGATTGGTAGAGTGGATGCAAAGCTAGCTCATGCTCAAAGCCCTTCTACTGGGATTAAAAAGACGCTT
ATTCATGGAACATTTGATGTAAGGACTGAACCAAATCCGATGTCATCACGTGATCCAAGAATAGCGCCGCATGATC
CTTTGAAGTTAGGGTGTGAAAAGCATGGCATGCCTTGTTCACCGTTTAATAGGAAACATCTGGAATTAGCGACAAA
TCATTTGAAAGAAAAATTAGTTTCTGTAGTTAAACCAATAAATGGTTGCAAGATTAGAAGTTTGCAAGATGCTGTA
TGTGGTGTGCCTGGATTAGATGGGTTTGATTCGATATCGTGGAATACTAGTGCTGGTTTTCCTTTGTCTTCATTAA
AGCCACCTGGAACATCAGGTAAGCGATGGTTGTTTGATATTGAGCTACAAGACTCGGGATGTTATCTCCTGCGTGG
AATGCGTCCCGAACTTGAGATTCAATTATCAACGACACAGTTAATGAGGAAAAAGGGAATAAAACCTCACACTATA
TTCACGGATTGTTTGAAAGATACTTGTTTGCCTGTTGAAAAATGTAGAATACCTGGTAAGACTAGAATATTTAGTA
TAAGTCCGGTACAGTTTACCATACCGTTTCGACAGTATTACCTAGACTTTATGGCATCCTATCGAGCTGCACGACT
TAATGCTGAGCATGGTATTGGTATTGATGTTAACAGCTTAGAGTGGACAAATTTGGCAACAAGGTTGTCTAAGTAT
GGCACTCACATCGTGACAGGAGACTATAAGAATTTTGGTCCTGGGTTAGATTCCGATGTTGCAGCTTCAGCGTTTG
AAATTATTATCGACTGGGTATTACATTACACCGAAGAAGATAATAAAGACGAAATGAAGCGAGTAATGTGGACCAT
GGCGCAAGAGATCTTAGCGCCTAGTCATCTATATCGCGATTTAGTGTACCGAGTACCTTGCGGAATTCCATCAGGT
TCTCCAATAACGGACATATTGAATACAATTTCAAATTGTTTGTTAATTAGGTTAGCTTGGTTAGGTATTACTGATT
TGCCTTTGTCCGAGTTCTCTCAAAATGTTGTTCTTGTCTGTTATGGCGACGATCTTATCATGAATGTTAGCGATAA
CATGATTGATAAGTTTAATGCCGTGACGATAGGAAAATTCTTTTCACAATATAAGATGGAATTTACGGATCAGGAT
AAATCAGGAAATACTGTAAAGTGGCGGACGTTACAAACTGCTACTTTCTTAAAACATGGGTTCTTAAAACATCCAA
CTAGACCTGTGTTTCTAGCTAACCTGGACAAAGTCTCGGTAGAAGGAACGACGAATTGGACTCATGCTCGAGGATT
GGGTCGTCGTACAGCAACCATAGAAAATGCTAAACAAGCGTTAGAGTTAGCATTTGGGTGGGGTCCTGAATATTTT
AATTATGTCAGAAATACCATTAAAATGGCTTTTGACAAGTTGGGTATTTATGAGGACCTTATCACATGGGAAGAAA
TGGATGTTAGATGTTACGCTAGCGCGTAATATTAAATTTTAAATATTCATTAATTTTAATTTTATTTTAGGTTATT
GGAATTGAGGGAAGTACCACCCCCCAAGACCTTCGTTTTAAATCTACTAAGAGGAGTAAACCTATATATAAGAGTC
TAAAGACAGAGTGGATTAGACCATCATCTTTAGCTTATATATGGGGAAGGTTGAGTTGCCTCTAAAGACTCAGCTC
CATAGTAGAGTAGTTTTAATTACGATTAAAGTGGTACTCTAGGTTAGGTGTTACTCGCGTATTATCAACTAGTGGT
AATGCGTCCTAATTTTAGTATAGTTTTAACCATAATAGTAAAAAAAAAAAAAAAAAAAAAAAAAAA

>DWV-USA-MD-7-cons
GGCGATTTATGCCTTCCATAGCGAATTACGGTGCAACTAACAATTTTAGATAGTAGCCATGAACAAACATTATAGT
GGCTCACTACGTATTGATCATTTTTATAATGACTTGCGTAGCATGAAGCGCATGCTTGTAGTTGTAACTATGTTAC
TTTGCAAGTTGGAGTTTACTATTTTGGATTATGAATATGTGCACTTAGTGTCTGTATTTATAGTCGTTTGTGGTTC
AAGGTTTTGTGTTAGTAGTACACTTATGTATGAATGTACCTTTAGTATGAATGTTATAGAATGACAATATCGAAAG
AAAAATCTTTATAAAATACAAAAATATTGTTTTTATTATTTCGATATGGTGTTTTATAGAGTAGATTGCCATGTGA
CCGCTCGTAGAAGTCCATTATGGTTTATCAATCGAAGTTGAATGTATTTATAAGAATATTATACTTAATTAGTAAT
ATTAGTAGTCCGTAACTATTATCATCCTTTTTCAGTTTGATGTGATAATAGACCACTGCAGTATCGAGTAGAGTTT
CGAATGCGTAGTGCAATAGTACAATCACTGTCACCGACCATCTATTGTAATGATAGATCTGTCGGAAACCATTATT
TATGAAGTGACTAGCAATCATGGATTAAATTAGATGGTATTCTAGTTTAGAGGTGATTCGGCGCTGCGGTGCGACT
GAAACTTCTAAATTAGCATGTCAGATTGTATTATGAATGCGTTAGTAGTAATTTCTGCGATAGAGCTGGGACCCCT
CAGTCTCTCAGGTATTGTACGAGGCGAAAGTGTGAAAGTTTTGTATGTGTTTTTATATGTACGACTGTATCGGGAA
TTCCTTTAGCAAGAATCCTTTTAATACAGTATAATCTGTGCTACGGTACGTTACGTTCGCAGGGCACCCGTTAATG
TCTCATAGCCCAGACGATGGCGGATGGAAAGACATCATATTTTATTTTAATGCTGTCTTTATTGCTGATTTATCTT
GCTGTTTTTATTTGCTATTTTATATTTGCTAATTTTCATTATTGCGAAATATATTACATTGCTATTTTTATTATAT
ACGCTAGATTCAATTTTATTTTTCCTATATTTTCAATTTAATTTTGATTTTGAAGGTAAATATATATAATTAATTA

TTAAAAATGGCCTTTAGTTGTGGAACTCTTTCTTACTCTGCCGTCGCCCAAGCTCCGTCTGTTGCCTATGCACCTC
GTACATGGGAAGCTGATGAAGCTAGGCGGCGCCGAGTCATTAAACGTTTGGCGCTGGAGCAAGAACGTATGCGTAA
CGTTCTTGACGTTGGCGTCTATGACCAGGCGACATGGGAACAGGAGGACGCGCGCGATAATGAGTTCCTAACGGAA
CAATTAAACAATTTATATACTATTTATTCGATCGCTGAACGTTGTACGCGTCGGCCTATTAAAGAGCACTCTCCTA
TATCAGTTTCGAATAGGTTTGCTCCATTGGAATCCCTCAAGGTCGAGGTCGGTCAAGAAGCAGGCGAATGTATATT
TAAGAAACCTAAATATACGCGCGTTTGCAAGAAAGTGAAGCGTGTTGCAACTCGCTTCGTTCGTGAAAAAGTTGTT
CGTCCTATGTGTTCTAGATCTCCTATGCTATTATTTAAGCTTAAGAAAATTATTTATGATTTGCACTTATATAGAT
TAAGAAAACAGATTAGGATGTTGAGACGTCAAAAACAGCGCGAGTATGAGTTAGAGTGTGTCACTAATCTGTTACA
ATTATCGAATCCGGTGCAGGCAAAACCAGAGATGGATAACCCTAATCCAGGACCTGATGGCGAGGGTGAAGTTGAA
TTAGAAAAGGATAGTAATGTTGTTTTAACAACTCAGCGAGATCCTAGTACATCTATTCCAGCGCCGGTGAGCGTAA
AATGGAGTAGATGGACTAGTAATGATGTAGTAGATGATTACGCCACAATCACATCTCGATGGTATCAGATTGCTGA
ATTTGTTTGGTCGAAGGATGATCCATTTGATAAGGAGTTAGCACGTTTAATTTTGCCTCGTGCTTTGTTATCTAGT
ATAGAGGCTAATTCTGATGCTATATGTGATGTGCCTAATACTATTCCATTTAAGGTACACGCATATTGGCGAGGCG
ATATGGAAGTTAGAGTTCAAATTAATTCAAATAAATTCCAAGTTGGTCAATTACAAGCTACTTGGTATTATTCGGA
TCATGAGAATTTGAATATATCGTCTAAGAGAAGCGTTTATGGATTTTCACAAATGGATCATGCTTTGATTAGTGCG
TCAGCAAGTAATGAAGCAAAATTAGTTATTCCATATAAGCATGTTTATCCATTTTTACCAACAAGAATTGTGCCAG
ATTGGACTACTGGCATTTTAGATATGGGTGCTTTGAACATTCGTGTAATTGCTCCCTTACGGATGAGTGCTACTGG
TCCAACTACCTGTAATGTCGTCGTGTTTATTAAATTAAATAACAGCGAGTTTACAGGGACTTCCTCTGGTAAGTTT
TATGCGAGCCAAATCAGGGCAAAACCTGAGATGGATCGTATATTAAATTTAGCAGAAGGATTGTTGAATAACACGA
TTGGTGGTAATAATATGGATAATCCTTCTTATCAACAATCTCCTCGTCATTTTGTCCCGACTGGTATGCACAGCTT
AGCTTTAGGTACTAATTTAGTTGAACCATTACATGCATTACGTTTGGATGCAGCCGGTACGACACAACATCCTGTA
GGTTGTGCTCCTGATGAAGATATGACTGTGTCTTCCATTGCATCTCGATATGGACTAATTAGACGGGTACAATGGA
AGAAAGATCATGCTAAAGGATCGCTTTTGTTACAATTAGATGCTGATCCATTTGTGGAGCAAAGAATTGAAGGTAC
GAATCCAATATCTTTGTATTGGTTCGCACCCGTGGGTGTAGTATCTAGTATGTTTATGCAATGGCGTGGTTCATTA
GAATATAGGTTTGATATTATAGCATCCCAATTTCATACTGGTAGGTTAATTGTAGGTTATGTGCCCGGTTTGACAG
CATCTTTGCAACTTCAAATGGACTATATGAAATTGAAGTCATCGAGTTATGTAGTATTTGATTTACAGGAAAGTAA
TAGCTTCACTTTTGAGGTGCCATATGTTTCATATAGACCATGGTGGGTGCGTAAATATGGTGGCAATTATTTGCCC
TCGTCAACTGACGCTCCTAGTACATTATTCATGTATGTGCAGGTTCCGTTGATACCTATGGAAGCTGTTTCAGATA
CTATTGATATCAATGTGTACGTACGGGGCGGTAGTTCATTTGAAGTTTGTGTTCCAGTCCAACCTAGTTTAGGTTT
GAATTGGAACACAGACTTTATTTTACGTAATGACGAAGAATACAGGGCTAAGACAGGTTATGCACCATATTATGCT
GGAGTGTGGCATAGCTTTAATAATAGTAATTCTCTTGTTTTTAGGTGGGGATCTGCTTCTGATCAAATTGCTCAGT
GGCCGACAATTTCAGTACCAAGAGGTGAGTTAGCTTTCTTACGAATTAAGGATGGAAAGCAAGCTGCTGTAGGAAC
TCAACCTTGGCGTACGATGGTTGTTTGGCCTTCTGGTCATGGCTATAATATTGGTATACCCACGTATAATGCTGAA
CGAGCTCGCCAGCTTGCACAACACTTATATGGTGGTGGATCATTAACTGATGAGAAGGCCAAACAATTATTTGTTC
CTGCTAATCAACAAGGACCTGGTAAGGTAAGTAATGGAAATCCGGTATGGGAAGTCATGCGTGCACCATTGTCAAC
ACAGCGTGCGCATATGCAAGATTTTGAATTTATTGAGGCTATTCCAGAAGGAGAGGAGTCTCGTAATACTACAGTC
TTGGATACGACCACTACTTTACAGTCGAGTGGGTTTGGTCGCGCCTTCTTTGGAGAAGCTTTTAATGATCTTAAAA
CGTTAATGCGGCGATATCAATTATATGGTCAATTATTATTGTCCGTTACTACGGATAAGGATATTGATCATTGTAT
GTTTACCTTCCCTTGTTTACCACAAGGGTTAGCGTTAGACATTGGTTCTGCTGGCTCTCCACATGAAATCTTTAAT
AGATGTCGTGATGGTATTATACCATTAATTGCATCTGGATATAGATTTTATAGAGGAGATTTGCGTTATAAGATTG
TTTTTCCAAGTAATGTTAATAGCAACATTTGGGTACAACATCGACCGGATCGTAGACTGGAAGGATGGTCCGCGGC
TAAGATTGTAAATTGTGATGCTGTGTCTACTGGTCAAGGGGTGTATAATCATGGTTATGCTAGTCACATTCAAATC
ACGCGTGTAAATAATGTTATAGAATTGGAAGTTCCATTTTATAATGCTACTTGTTATAATTATTTACAGGCGTTTA
ATGCGTCTAGCGCTGCATCTAGTTATGCAGTATCTTTAGGAGAAATATCGGTTGGTTTTCAAGCTACAAGTGATGA
TATTGCATCTATCGTCAACAAACCTGTTACTATTTATTATAGTATTGGAGATGGTATGCAGTTTTCTCAGTGGGTT
GGATATCAACCGATGATGATCCTAGATCAGCTTCCTGCACCAGTAGTAAGGGCCGTGCCTGAGGGCCCTATTGCGA
AGATTAAAAACTTCTTCCATCAAACAGCCGACGAAGTTAGAGAAGCTCAGGCAGCAAAGATGCGTGAAGATATGGG
TATGGTTGTYCAAGATGTTATTGGAGAACTTAGCCAGGCCATACCGGATCTTCAACAACCGGAGGTTCAAGCAAAT
GTCTTCTCACTGGTGTCTCAGTTAGTGCATGCTATTATAGGTACTAGTTTGAAGACAGTAGCTTGGGCGATTGTTT
CGATTTTTGTGACTTTAGGATTAATTGGACGCGAAATGATGCATTCAGTTATTACTGTAGTCAAGCGGTTACTTGA
GAAATATCACTTGGCGACGCAACCCCAGGAATCCGCCAGTTCAAGTACTGTTATATCTGCCGTTCCAGAAGCTCCC
AATGCTGAAGCGGAGGAGGCAAGTGCTTGGGTATCCATTATTTATAATGGTGTGTGTAATATGCTTAATGTGGCTG
CTCAAAAACCGAAACAATTTAAAGATTGGGTAAAATTAGCTACTGTAGATTTTAGTAATAATTGTAGAGGTAGTAA
TCAGGTATTTGTGTTTTTCAAGAATACATTTGAAGTGTTGAAGAAAATGTGGGGTTATGTATTTTGTCAGAGTAAT
CCTGCAGCGCGTTTGTTGAAAGCTGTGAATGACGAGCCTGAGATTTTGAAAGCATGGGTGAAGGAATGTCTGTATT
TGGATGATCCCAAATTTAGAATGCGTCGAGCGCATGATCAAGAGTATATCGAGCGAGTGTTTGCGGCACATTCATA
TGGACAAATTTTGCTACATGATTTAACTGCTGAAATGAACCAATCACGAAATTTGAGTGTGTTTACACGTGTGTAT
GATCAAATTTCAAAATTGAAGACTGATCTTATGGAAATGGGATCGAATCCATATATAAGGCGTGAATGTTTTACGA
TATGCATGTGTGGTGCATCTGGAATTGGAAAATCATATTTGACCGATTCTTTATGCAGCGAGCTCTTACGTGCGAG

TCGTACTCCTGTGACAACAGGCATAAAATGTGTTGTTAATCCATTATCTGATTATTGGGATCAGTGTGATTTTCAG
CCTGTTTTGTGCGTTGATGATATGTGGAGTGTTGAAACATCTACTACGCTCGATAAGCAGTTGAACATGCTTTTCC
AGGTCCATTCCCCTATCGTGCTTTCTCCTCCTAAAGCTGATTTAGAAGGTAAGAAAATGCGATATAACCCGGAAAT
ATTTATATACAATACGAATAAGCCTTTCCCGAGGTTTGATCGTATTGCTATGGAAGCTATTTATCGGCGTAGAAAT
GTTTTGATTGAATGTAAAGCGAGTGAAGAGAAGAAGCGAGGATGCAAACATTGTGAGAACGATATTCCTATTGCTG
AATGTAGTCCTAAGATGTTGAAAGATTTCCATCACATCAAATTTAGGTATGCACATGATGTATGTAATTCCGAGAC
CACATGGTCTGAATGGATGACGTATAATGAATTTCTTGAATGGATAACTCCTGTGTATATGGCTAACCGTCGTAAG
GCGAATGAATCGTTTAAGATGCGTGTAGATGAAATGCAAATGTTACGTATGGATGAGCCATTGGAAGGTGATAATA
TCCTCAATAAGTATGTTGAAGTTAATCAGCGCTTAGTGGAGGAAATGAAGGCATTTAAGGAGCGTACACTATGGTC
AGATTTACATCGTTTAGGTGCGGAAATTAGTGCGTCAGTTAAGAAAGCTTTACCAACTATTTCTATAACCGAAAAA
CTACCACATTGGACTGTTCAATGTGGTATTGCTAAACCTGAGATGGATCATGCTTATGAGGTTATGAGTTCGTATG
CAGCTGGAATGAATGCAGAGATTGAAGCGCATGAACAAGTTCGGCGTTCATCAGTGGAATGTCAATATGCAGAGCC
TCAAGCTCCAAGAAATCCTGATGATGAAGGACCAACCATAGATGAAGAACTTATGGGCGACACTGAATTCACATCA
CAGGCTCTAGAACGTCTTGTGGATGAAGGTTATATAACTGGAAAACAGAAGAAATATATAGCTACGTGGTGTAGTA
AGCGTCGTGAACATACTGCTGACTTTGATCTTGTGTGGACTGATAATTTGCGTGTGTTAAGCGCGTATGTGCATGA
ACGTTCATCTTCAACTCGGCTTTCTACGGATGACGTCAAGTTATATAAAACGATTAGCATGTTACATCAAAAGTAT
GATACCACAGAGTGTGCTAAATGTCAACATTGGTATGCTCCGTTGACTGATATCTATGTTGATGACAAGAAATTGT
TCTGGTGTCAGAAAGAGAAAAAGACACTTATTGATGTCCGAAAATTGTCGAAAGAAGATGTGACTGTTCAATCGAA
ATTGATTAATTTATCTGTTCCTTGTGGTGAAGTATGTATGTTACATTCAAAATATTTCAATTATCTTTTCCATAAA
GCATGGTTGTTCGAGAACCCAACTTGGCGCCTAATATATAATGGTACCAAGAAGGGTATGCCTGAGTACTTTATGA
ATTGTGTGGATGAAATATCATTAGATTCCAAATTTGGTAAAGTGAAAGTATGGTTGCAAGCGATCATTGATAAGTA
TTTAACTCGTCCCGTGAAAATGATTCGTGATTTTCTTTTCAAGTGGTGGCCGCAAGTTGCGTATGTGTTGAGCTTG
CTAGGTATAATTGGTATAACTGCGTATGAAATGAGAAATCCGAAACCAACTTCTGAGGAATTAGCTGATCATTATG
TGAATAGGCATTGTAGCTCTGATTTTTGGTCACCAGGACTGGCATCACCTCAAGGATTGAAATATAGTGAAGCAGT
AACAGTAAAGGCACCTAGAATCCATAGATTGCCAGTTACTACTAAGCCTCAGGGATCAACACAACAAGTAGACGCT
GCTGTGAATAAAATTCTACAGAACATGGTTTATATTGGTGTTGTTTTCCCAAAAGTGCCTGGTAGTAAGTGGCGAG
ATATTAATTTTAGGTGTCTTATGCTTCATAATAGGCAATGTTTAATGTTGAGGCATTATATTGAGTCAACTGCCGC
CTTTCCTGAGGGAACCAAGTACTATTTTAAGTATATTCATAATCAAGAGACTAGAATGTCTGGTGATATTTCTGGT
ATTGAAATTGATTTGTTGAATTTACCTAGATTGTATTATGGTGGTCTCGCGGGAGAGGAGTCGTTTGATAGTAATA
TCGTGCTTGTGACTATGCCTAATCGTATTCCTGAGTGTAAGAGCATTATTAAATTTATAGCGTCACATAATGAGCA
TATACGTGCTCAGAATGATGGAGTGTTAGTAACTGGCGACCATACTCAGCTATTGGCTTTCGAGAATAATAATAAA
ACTCCAATAAGTATTAACGCTGATGGTTTGTATGAGGTTATACTTCAAGGAGTATATACCTATCCATACCACGGCG
ATGGTGTTTGTGGTTCGATATTGTTGTCTCGGAATTTACAACGGCCAATTATAGGTATCCATGTTGCTGGTACTGA
AGGATTGCATGGCTTTGGAGTCGCTGAACCACTTGTACATGAAATGTTCACCGGTAAAGCRATCGAGAGTGAAAGA
GAGCCGTATGATCGTGTGTATGAACTTCCGTTGCGTGAATTAGATGAATCTGATATTGGTTTAGATACCGATTTAT
ATCCGATTGGTAGAGTGGATGCAAAGCTAGCTCATGCTCAAAGCCCTTCTACTGGGATTAAAAAGACGCTTATCCA
TGGAACATTTGATGTAAGGACTGAACCAAATCCGATGTCATCACGTGATCCAAGAATAGCGCCGCATGATCCTTTG
AAGTTAGGGTGTGAAAAGCATGGCATGCCTTGTTCACCGTTTAATAGGAAACATCTGGAATTAGCGACAAATCATT
TGAAAGAAAAATTAGTTTCAGTAGTTAAACCAATAAATGGTTGCAAGATTAGAAGTTTGCAAGATGCTGTATGTGG
TGTGCCTGGATTAGATGGGTTTGATTCGATATCTTGGAATACTAGTGCTGGTTTTCCTTTGTCTTCATTAAAGCCA
CCTGGAACATCAGGTAAGCGATGGTTGTTTGACATTGAGCTACAAGACTCGGGATGTTATCTCCTGCGTGGAATGC
GTCCCGAACTTGAGATTCAATTATCAACGACACAGTTAATGAGGAAAAAGGGAATAAAACCTCACACTATATTCAC
GGATTGTTTGAAAGATACTTGTTTGCCTGTTGAAAAATGTAGAATACCTGGTAAGACTAGAATATTTAGTATAAGT
CCGGTACAGTTTACCATACCGTTTCGACAGTATTACTTAGACTTTATGGCATCCTATCGAGCTGCACGACTTAATG
CTGAGCATGGTATTGGTATTGATGTTAACAGCTTAGAGTGGACAAATTTGGCAACAAGGTTGTCTAAGTATGGCAC
TCACATCGTGACAGGAGACTATAAGAATTTTGGTCCTGGGTTAGATTCCGATGTTGCAGCTTCAGCGTTCGAAATT
ATTATCGACTGGGTATTACATTACACCGAAGAAGATAATAAAGACGAAATGAAGCGAGTAATGTGGACCATGGCGC
AAGAGATCTTAGCGCCTAGTCATCTATATCGCGATTTGGTGTACCGAGTACCTTGCGGAATTCCATCAGGTTCTCC
AATAACGGACATATTGAATACAATTTCAAATTGTTTGTTAATTAGGTTAGCTTGGTTAGGTATTACTGATTTGCCT
TTGTCCGAGTTCTCTCAAAATGTTGTTCTTGTCTGTTATGGCGACGATCTTATCATGAATGTTAGCGATAACATGA
TTGATAAGTTTAACGCCGTGACGATAGGAAAATTCTTTTCACAATATAAGATGGAATTTACGGATCAGGATAAATC
AGGAAATACTGTAAAGTGGCGGACGTTACAGACTGCTACTTTCTTAAAACATGGGTTCTTAAAACATCCAACTAGA
CCTGTGTTTCTAGCTAACCTGGACAAGGTCTCGGTAGAAGGAACGACGAATTGGACTCATGCTCGAGGATTGGGTC
GTCGTACAGCAACCATAGAAAATGCTAAACAAGCGTTAGAGTTAGCATTTGGGTGGGGTCCTGAATATTTTAATTA
TGTCAGAAATACCATTAAAATGGCTTTTGACAAGTTGGGTATTTATGAGGACCTTATCACATGGGAAGAAATGGAT
GTTAGATGTTACGCTAGCGCGTAATATTAAATTTTAAATATTCATTAATTTTAATTTTATTTTAGGTTATTGGAAT
TGAGGGAAGTACCACCCCCCAAGACCTTCGTTTTAAATCTACTAAGAGGAGTAAACCTATATATAAGAGTCTAAAG
ACAGAGTGGATTAGACCATCATCTTTAGCTTATATATGGGGAAGGTTGAGTTGCCTCTAAAGACTCAGCTCCATAG

TAGAGTAGTTTTAATTACGATTAAAGTGGTACTCTAGGTTAGGTGTTACTCGCGTATTATCAACTAGTGGTAATGC
GTCCTAATTTTAGTATAGTTTTAACCATAATAGTAAAAAAAAAAAAAAAAAAAAAAAAAAA

>DWV-USA-MD-12-cons
TGGGAGGCGATTTATGCCTTCCATAGCGAATTACGGTGCAACTAACAATTTTAGATAGTAGCCATGAACAAACATT
ATAGTGGCTCACTACGTATTGATCATTTTTATAATGACTTGCGTAGCATGAAGCGCATGCTTGTAGTTGTAACTAT
GTTACTTTGCAAGTTGGAGTTTACTATTTTGGATTATGAATATGTGCACTTAGTGTCTGTATTTATAGTCGTTTGT
GGTTCAAGGTTTTGTGTTAGTAGTACACTTATGTATGAATGTACCTTTAGTATGAATGTTATAGAATGACAATATC
GAAAGAAAAATCTTTATAAAATACAAAAATATTGTTTTTATTATTTCGATATGGTGTTTTATAGAGTAGATTGCCA
TGTGACCGCTCATAGAAGTCCATTATGGTTTATCAATCGAAGTTGAATGTACTTATAAGAATGTTATACTTAATCA
GTATTATTAGTAGTCCGTAACTATTATCATCCTTTTTCAGTTTGATGTGATAATAGACCACTGCAGTATCGAGTAG
AGTTTCGAATGCGTAGTGCAATAGTACAATCACTGTCACCGACCATCTATTGTAATGATAGATCTGTCGGAAACCA
TTATTTATGAAGTGACTAGCAATCATGGATTAAATTAGATGGTATTCTAGTTTAGAGGTGATTCGGCGCTGCGGTG
CGACTGAAACTTCTAAATTAGCATGTCAGATTGTATTATGAATGCGTTAGTAGTAATTTCTGCGATAGAGCTGGGA
CCCCTCAGTCTCTCAGGTATTGTACGAGGCGAAAGTGTGAAAGTTTTGTATGCGTTTTTATATGTACGACTGTATC
GGGAATTCCTTTAGCAAGAATCCTTTTAATACAGTATAATCTGTGCTACGGTACGTTACGTTCGCAGGGCACCCGT
TAATGTCTCATAGCCCAGACGATGGCGGATGGAAAGACATCATATTTTATTTTAATGCTGTCTTTATTGCTGATTT
ATCTTGCTGTTTTTATTTGCTATTTTATATTTGCTAATTTTCATTATTGCGAAATATATTACATTGCTATTTTTAT
TATATACGCTAGATTCAATTTTATTTTTCCTATATTTTCAATTTAATTTTGATTTCGAAGGTATATATATATATAATT
AATTATTAAAAATGGCTTTTAGTTGTGGAACTCTTTCTTACTCTGCCGTCGCCCAAGCTCCGTCTGTTGCCTATGC
ACCTCGTACATGGGAAGCTGATGAAGCTAGGCGGCGCCGAGTTATTAAACGTTTGGCGCTGGAGCAAGAACGTATG
CGTAACGTTCTTGACGTTAGCGTCTATGACCAGGCGACATGGGAACAGGAGGACGCGCGCGATAATGAGTTCCTAA
CGGAACAATTAAACAATTTATATACTATTTATTCGATCGCTGAACGTTGTACGCGTCGGCCTATCAAAGAGCACTC
TCCTATATCAGTTTCGAATAGGTTTGCTCCACTGGAATCCCTCAAGGTCGAGGTCGGTCAAGAAGCAGGCGAATGT
ATATTTAAGAAACCTAAATATACACGCGTTTGCAAGAAAGTGAAGCGTGTTGCAACTCGCTTCGTTCGTGAAAAAG
TTGTTCGTCCTATGTGTTCTAGATCTCCTATGCTACTATTTAAGCTTAAGAAAATTATTTATGATTTGCACTTATA
TAGATTAAGAAAACAGATTAGGATGTTGAGACGTCAAAAACAGCGCGAATATGAGTTAGAGTGTGTCACTAATCTG
TTACAATTATCGAATCCGGTGCAGGCAAAACCAGAGATGGATAACCCTAATCCAGGACCTGATGGCGAGGGTGAAG
TTGAACTAGAAAAGGATAGTAATGTTGTTTTAACAACTCAGCGAGATCCTAGTACATCTATTCCAGCGCCGGTGAG
CGTAAAATGGAGTAGATGGACTAGTAATGATGTAGTAGATGATTACGCCACAATCACATCTCGATGGTATCAGATT
GCTGAATTTGTTTGGTCGAAGGATGATCCATTTGATAAGGAGTTAGCACGTTTAATTTTGCCTCGTGCTTTGTTAT
CTAGTATAGAGGCTAACTCTGATGCTATATGTGATGTGCCTAATACTATCCCATTTAAGGTACACGCATATTGGCG
AGGCGATATGGAAGTTAGAGTTCAAATTAATTCAAATAAATTCCAAGTTGGTCAATTACAAGCTACTTGGTATTAT
TCGGATCATGAGAATTTGAATATATCGTCTAAGAGAAGCGTTTATGGATTTTCACAAATGGATCATGCTTTGATTA
GTGCGTCAGCAAGTAATGAAGCAAAATTAGTTATTCCATATAAGCATGTTTATCCATTTTTACCAACAAGAATTGT
GCCAGATTGGACTACTGGCATTTTAGACATGGGTGCTTTGAATATTCGTGTAATTGCTCCCTTACGGATGAGTGCT
ACTGGTCCAACTACCTGTAATGTCGTCGTGTTTATTAAATTAAATAACAGCGAGTTTACAGGGACTTCTTCTGGTA
AGTTTTATGCGAGCCAAATCAGGGCAAAACCTGAGATGGATCGTATATTAAATTTAGCAGAGGGATTGTTGAATAA
CACGATTGGTGGTAATAATATGGATAATCCTTCTTATCAGCAATCTCCTCGTCATTTTGTCCCGACTGGTATGCAT
AGCTTAGCTTTAGGTACTAATTTAGTTGAACCATTACATGCATTACGTTTGGATGCAGCCGGTACGACACAACATC
CTGTAGGTTGTGCTCCTGATGAAGATATGACTGTATCTTCCATTGCATCTCGATATGGACTAATTAGACGGGTACA
ATGGAAGAAAGATCATGCTAAAGGATCACTTTTGTTACAATTAGATGCTGATCCATTTGTGGAGCAAAGAATTGAA
GGTACGAATCCAATATCTTTGTATTGGTTCGCACCCGTGGGTGTAGTATCTAGTATGTTTATGCAATGGCGTGGTT
CATTAGAATATAGATTTGATATTATAGCATCCCAATTTCATACTGGTAGGTTAATTGTAGGTTATGTGCCCGGTTT
GACAGCATCTTTGCAACTTCAAATGGACTATATGAAATTGAAGTCATCGAGTTATGTAGTATTTGATTTACAGGAA
AGTAATAGCTTCACTTTTGAGGTGCCATATGTTTCATATAGACCATGGTGGGTGCGTAAATATGGTGGCAATTATT
TACCCTCGTCAACTGACGCTCCTAGTACATTATTTATGTATGTGCAGGTTCCGCTGATACCTATGGAAGCTGTTTC
AGATACTATTGATATCAATGTGTACGTACGGGGCGGTAGTTCATTTGAAGTTTGTGTTCCGGTCCAACCTAGTTTA
GGTTTGAATTGGAACACAGACTTTATTTTACGTAATGACGAAGAATACAGGGCTAAGACAGGTTATGCACCATATT
ATGCTGGAGTGTGGCATAGCTTCAATAATAGTAATTCTCTTGTTTTTAGGTGGGGATCTGCTTCTGATCAAATTGC
TCAGTGGCCGACAATTTCAGTACCAAGAGGTGAGTTAGCTTTCTTACGAATTAAGGATGGAAAGCAAGCTGCTGTA
GGAACTCAACCTTGGCGTACGATGGTTGTTTGGCCTTCTGGTCATGGCTATAATATTGGTATACCTACGTATAATG
CTGAACGAGCTCGCCAGCTTGCACAACACTTATATGGTGGTGGATCATTAACTGATGAGAAGGCCAAACAATTATT
TGTTCCTGCTAATCAACAAGGACCTGGTAAGGTAAGTAATGGAAATCCGGTATGGGAAGTCATGCGTGCACCATTG
TCAACACAGCGTGCGCATATGCAAGATTTTGAATTTATTGAAGCTATTCCAGAAGGAGAGGAGTCTCGTAATACTA
CGGTCTTGGATACGACCACTACTTTACAGTCGAGTGGATTTGGTCGCGCCTTCTTTGGAGAAGCTTTTAATGACCT
TAAAACGTTAATGCGGCGATATCAATTATATGGTCAATTATTATTGTCCGTTACTACGGATAAGGATATTGATCAT

TGTATGTTTACCTTCCCTTGTTTACCACAAGGGTTAGCGTTAGACATTGGTTCTGCTGGCTCTCCACATGAAATCT
TTAATAGATGTCGTGATGGTATTATACCATTAATTGCATCTGGATATAGATTTTATAGAGGAGATTTGCGTTATAA
GATTGTTTTTCCAAGTAATGTTAATAGCAACATTTGGGTACAACATCGACCGGATCGTAGACTGGAAGGATGGTCC
GCGGCTAAGATTGTAAATTGCGATGCTGTGTCTACTGGTCAAGGGGTGTATAATCATGGTTATGCTAGTCACATTC
AAATCACGCGTGTAAATAATGTTATAGAATTGGAAGTTCCATTTTATAATGCTACTTGTTATAATTATTTGCAGGC
GTTTAATGCGTCTAGCGCTGCATCTAGTTATGCAGTATCTTTAGGAGAAATATCGGTTGGTTTTCAAGCTACAAGT
GATGATATTGCATCTATTGTTAACAAACCTGTTACTATTTATTATAGTATTGGAGATGGTATGCAATTTTCTCAGT
GGGTTGGATATCAACCGATGATGATCCTAGACCAGCTTCCTGCACCAGTAGTAAGGGCCGTGCCTGAGGGCCCTAT
TGCGAAGATTAAAAACTTCTTCCATCAAACAGCCGACGAAGTTAGAGAAGCTCAGGCAGCGAAGATGCGTGAAGAT
ATGGGTATGGTTGTCCAAGATGTTATTGGAGAACTTAGCCAGGCCATACCGGATCTTCAACAACCGGAGGTTCAAG
CAAATGTCTTCTCACTGGTGTCTCAGTTAGTGCATGCTATTATAGGTACTAGTTTGAAGACAGTAGCTTGGGCGAT
TGTTTCGATTTTTGTGACTTTAGGATTAATTGGACGCGAAATGATGCATTCAGTTATTACTGTAGTCAAGCGGTTA
CTTGAGAAATATCACTTGGCGACGCAACCCCAGGAATCCGCCAGTTCAAGTACTGTTATATCTGCCGTTCCAGAAG
CTCCCAATGCTGAAGCGGAGGAGGCAAGTGCTTGGGTATCCATTATTTATAATGGTGTGTGTAATATGCTTAATGT
GGCTGCTCAAAAACCGAAACAATTTAAAGATTGGGTAAAATTAGCTACTGTAGATTTTAGTAATAATTGTAGAGGT
AGTAATCAGGTATTTGTATTTTTCAAGAATACATTTGAAGTGTTGAAGAAAATGTGGGGTTATGTATTTTGTCAGA
GTAATCCTGCAGCGCGTTTGTTGAAAGCTGTGAATGACGAGCCTGAGATTTTGAAAGCATGGGTGAAGGAATGTCT
GTATTTGGATGATCCTAAATTTAGAATGCGTCGAGCGCATGATCAAGAGTATATCGAGAGAGTGTTTGCGGCACAT
TCGTATGGACAAATTTTGCTACATGATTTAACTGCTGAAATGAATCAATCACGAAATTTGAGTGTGTTTACACGTG
TGTATGATCAAATTTCAAAATTGAAGACTGATCTTATGGAAATGGGATCGAATCCATATATAAGGCGCGAATGTTT
TACGATATGCATGTGTGGTGCATCTGGAATTGGAAAATCATATTTGACCGATTCTTTATGCAGCGAGCTCTTACGT
GCGAGTCGTACTCCTGTGACAACAGGCATAAAATGTGTTGTTAATCCATTATCTGATTATTGGGATCAGTGTGATT
TTCAGCCTGTTTTGTGCGTTGATGATATGTGGAGTGTTGAAACATCTACTACGCTCGATAAGCAGTTGAACATGCT
TTTCCAGGTCCATTCCCCTATCGTGCTTTCTCCTCCTAAAGCTGATTTAGAAGGTAAGAAAATGCGATATAACCCG
GAAATATTTATATACAATACGAATAAGCCTTTCCCGAGGTTTGATCGTATTGCTATGGAAGCTATTTATCGGCGTA
GAAATGTTTTGATTGAATGTAAAGCGAGTGAAGAGAAGAAGCGAGGATGCAAGCATTGTGAGAATGATATTCCTAT
TGCTGAATGTAGTCCTAAGATGTTGAAAGATTTCCATCATATCAAATTTAGGTATGCACATGATGTATGTAATTCC
GAGACCACATGGTCTGAATGGATGACGTATAATGAATTTCTCGAATGGATAACTCCTGTGTATATGGCTAACCGTC
GTAAGGCGAATGAATCGTTTAAGATGCGTGTAGATGAAATGCAAATGTTACGTATGGATGAACCATTGGAAGGTGA
TAATATTCTCAATAAGTATGTTGAGGTTAATCAGCGTTTAGTGGAGGAAATGAAGGCATTTAAGGAGCGTACACTA
TGGTCAGATTTACATCGCGTAGGTGCGGAAATTAGTGCATCAGTTAAGAAAGCTTTACCAACTATTTCTATAACCG
AAAAACTACCACATTGGACTGTTCAATGTGGTATTGCTAAACCTGAGATGGATCATGCTTATGAGGTTATGAGTTC
GTATGCAGCTGGAATGAATGCAGAGATTGAAGCGCATGAACAAGTTCGGCGTTCATCAGTGGAATGTCAATATACA
GAGCCTCAAGCTCCAAGAAATCCTGATGATGAAGGACCAACTATAGATGAAGAACTTATGGGCGACACTGAATTCA
CATCACAGGCTCTAGAACGTCTTGTGGATGAAGGTTATATAACTGGAAAACAGAAGAAATATATAGCTACGTGGTG
TAGTAAGCGTCGTGAACATACTGCTGACTTTGATCTTGTGTGGACTGATAATTTGCGTGTGTTAAGCGCGTATGTG
CACGAACGTTCATCTTCAACTCGGCTTTCTACGGATGACGTCAAGTTATATAAAACGATTAGTATGTTACATCAAA
AGTATGATACCCACAGAGTGTGCTAAATGTCAACATTGGTATGCTCCGTTGACTGACATCTATGTTGATGACAAGAA
ATTGTTCTGGTGTCAGAAAGAGAAAAAGACACTTATTGATGTCCGAAAATTGTCGAAAGAAGATGTGACTGTTCAA
TCGAAATTGATTAATTTATCTGTTCCTTGTGGTGAAGTATGTATGTTACATTCAAAATATTTCAATTATCTTTTCC
ATAAAGCATGGTTGTTTGAGAACCCGACTTGGCGCCTAATATATAATGGTACCAAGAAGGGTATGCCTGAGTACTT
TATGAATTGTGTGGATGAAATATCATTAGATTCCAAATTTGGTAAAGTGAAAGTATGGTTGCAAGCGATCATTGAT
AAGTATTTAACTCGTCCCGTGAAAATGATTCGTGATTTTCTTTTCAAGTGGTGGCCGCAAGTTGCGTATGTGTTGA
GCTTGCTAGGTATAATTGGTATAACTGCGTATGAAATGAGAAATCCGAAACCAACTTCTGAGGAATTAGCTGATCA
TTATGTGAATAGGCATTGTAGCTCTGATTTTTGGTCACCAGGACTGGCATCACCTCAAGGATTGAAATATAGTGAA
GCAGTAACAGTAAAGGCACCTAGAATCCATAGATTGCCAGTTACTACTAAGCCTCAGGGATCAACACAACAAGTAG
ACGCTGCTGTGAATAAAATTTTACAGAACATGGTTTATATTGGTGTTGTTTTCCCAAAAGTACCTGGTAGTAAGTG
GCGAGATATTAATTTTAGGTGTCTTATGCTTCATAATAGGCAATGTTTAATGTTGAGGCACTATATTGAGTCAACT
GCCGCCTTTCCTGAGGGAACCAAGTACTATTTTAAGTATATTCATAATCAAGAGACTAGAATGTCTGGTGATATTT
CTGGTATTGAAATTGATTTGTTGAATTTACCTAGATTGTATTATGGTGGTCTCGCGGGAGAGGAGTCGTTTGATAG
TAATATCGTGCTTGTGACTATGCCTAATCGTATTCCTGAGTGTAAGAGCATTATTAAATTTATAGCGTCACATAAT
GAGCATATACGTGCTCAGAATGATGGAGTGTTAGTAACTGGCGACCATACTCAGCTATTGGCTTTCGAGAATAATA
ATAAAACTCCAATAAGTATTAACGCTGATGGTTTGTATGAGGTTGTACTTCAAGGAGTATATACCTATCCATACCA
CGGCGATGGTGTTTGTGGTTCGATATTGTTGTCTCGAAATTTACAACGGCCGATTATAGGTATCCATGTTGCTGGT
ACTGAAGGATTGCATGGCTTTGGAGTTGCTGAACCACTTGTACATGAAATGTTCACTGGTAAAGCAATCGAGAGTG
AAAGAGAGCCGTATGATCGTGTGTATGAACTTCCGTTGCGTGAATTAGATGAATCTGATATTGGTTTAGATACCGA
TTTATATCCGATTGGTCGAGTGGATGCAAAGCTAGCCCATGCTCAAAGCCCTTCTACTGGGATTAAAAAGACGCTT
ATCCATGGAACATTTGATGTAAGGACTGAACCAAATCCGATGTCATCACGTGATCCAAGAATAGCGCCACATGATC
CTTTGAAGTTAGGGTGTGAAAAGCATGGCATGCCTTGTTCACCGTTTAATAGGAAACATCTGGAATTAGCGACAAA

TCATTTGAAAGAAAAATTATTTTCAGTAGTTAAACCAATAAATGGTTGCAAGATTAGAAGTTTGCAAGATGCTGTA
TGTGGTGTGCCTGGATTAGATGGGTTTGATTCGATATCTTGGAATACTAGTGCTGGTTTTCCTTTGTCTTCATTAA
AGCCACCTGGAACATCAGGCAAGCGATGGTTGTTTGACATTGAGCTACAAGATTCGGGATGTTATCTCCTGCGTGG
AATGCGTCCCGAACTTGAGATTCAATTATCAACGACACAGTTAATGAGAAAAAAGGGAATAAAACCTCACACTATA
TTCACGGATTGTTTGAAAGATACTTGTTTGCCTGTTGAAAAATGTAGAATACCTGGGAAGACTAGAATATTTAGTA
TAAGTCCGGTACAGTTTACCATACCGTTTCGACAGTATTACTTAGACTTTATGGCATCCTATCGAGCTGCACGGCT
TAATGCTGAGCATGGTATTGGTATTGATGTTAACAGCTTAGAGTGGACAAATTTGGCAACAAGATTGTCTAAGTAT
GGCACTCACATCGTGACAGGAGACTATAAGAATTTTGGTCCTGGGTTAGATTCCGATGTTGCAGCTTCAGCGTTCG
AAATTATTATCGACTGGGTATTACATTACACCGAAGAAGATAATAAAGACGAAATGAAGCGAGTAATGTGGACCAT
GGCGCAAGAGATCTTAGCGCCTAGTCATCTATGTCGCGACTTGGTGTACCGAGTACCTTGCGGAATTCCATCAGGT
TCTCCAATAACGGACATATTGAATACAATTTCAAATTGTTTGTTAATTAGGTTAGCTTGGTTAGGTATTACTGATT
TGCCTTTGTCCGAGTTCTCTCAAAATGTTGTTCTTGTCTGTTATGGCGATGATCTTATCATGAATGTTAGCGACAA
CATGATTGATAAGTTTAACGCCGTGACGATAGGAAAATTCTTTTCACAATATAAGATGGAATTTACGGATCAGGAT
AAATCAGGAAATACTGTAAAGTGGCGGACGTTACAGACTGCCACTTTCTTAAAACATGGGTTTTTAAAACATCCAA
CTAGACCTGTGTTTCTAGCTAACCTGGACAAGGTCTCGGTAGAAGGAACGACGAATTGGACTCATGCTCGAGGATT
GGGTCGTCGTACAGCAACCATAGAAAATGCTAAACAAGCGTTAGAGTTAGCATTTGGGTGGGGTCCTGAATATTTT
AATTATGTCAGAAATACCATTAAAATGGCTTTTGACAAGTTGGGTATTTATGAGGACCTTATCACATGGGAAGAAA
TGGATGTTAGATGTTACGCTAGCGCGTAATATTAAATTTTAAATATTCATTAATTTTAATTTTATTTTAGGTTATT
GGAATTGAGGGAAGTACCACCCCCCAAGACCTTCGTTTTAAATCTACTAAGAGGAGTAAACCTATATATAAGAGTC
TAAAGACAGAGTGGATTAGACCATCATCTTTAGCTTATATATGGGAAAGGTTGAGTTGCCTCTAAAGACTCAGCTC
CATAGTAGAGTAGTTTTAATTACGATTAAAGTGGTACTCTAGGTTAGGTGTTACTCGCGTATTATCAATTAGTGGT
AATGCGTCCTAATTTTAGTATAGTTTTAACCATAATAGTAAAAAAAAAAAAAAAAAAAAAAAAAAA

>DWV-USA-FL-22-cons
TGGGAGGCGATTTATGCCTTCCATAGCGAATTACGGTGCAACTAACAATTTTAGATAGTAGCCATGAACAAACATT
ATAGTGGCTCACTACGTATTGATCATTTTTATAATGACTTGCGTAGCATGAAGCGCATGCTTGTAGTTGTAACTAT
GTTACTTTGCAAGTTGGAGTTTACTATTTTGGATTATGAATATGTGCACTTAGTGTCTGTATTTATAGTCGTTTGT
GGTTCAAGGTTTTGTGTTAGTAGTACACTTATGTATGAATGTACCTTTAGTATGAATGTTATAGAATGACAATATC
GAAAGAAAAATCTTTATAAAATACAAAAATATTGTTTTTATTATTTCGATATGGTGTTTTATAGAGTAGATTGCCA
TGTGACCGCTCGTAGAAGTCCATTATGGTTTATCAATCGAAGTTGAATGTATTTATAAGAATATTATACTTAATTA
ATAATATTAGTAGTCCGTAACTATTATCATCCTTTTTCAGTTTGATGTGATAATAGACCACTGCAGTATCGAGTAG
AGTTTCGAATGCGTAGTGCAATAGTATAATCACTGTCACCGACCATCTATTGTAATGATAGATCTGTCGGAAACCA
TTATTTATGAAGTGACTAGCAATCATGGATTAAATCAGATGGTATTCTAGTTTAGAGGTGATTCGGCGCTGCGGTG
CGACTGAAACTTCTAAATTAGCATGTCAGATTATATTATGAATGCGTTAGTAGTAATTTCTGCGATAGAGCTGGGA
CCCCTCAGTCTCTCAGGTATTGTACGAGGCGAAAGTGTGAAAGTTTTGTATGTGTTTTTATATGTACGACTGTATC
GGGAATTCCTTTAGCAAGAATCCTTTTAATACAGTATAATTTGTGCTACGGTACGTTACGTTCGCAGGGCACCCGT
TAATGTCTCATAGCCCAGACGATGGCGGATGGAAAGACATCATATTTTATTTTAATGCTGTCTTTATTGCTGATTT
ATCTTGCTGTTTTTATTTGCTATTTCATATTTGCTAATTTTCATTATTGCGAAATATATTACATTGTTATTTTTAT
TATATACGCTAGATTCAATTTTATTTTTCTTATATTTTCAATTTAATTTTGATTTTGAAGGTAAATATATATAATT
AATTATTAAAAATGGCTTTTAGTTGTGGAACTCTTTCTTACTCTGCCGTCGCCCAAGCTCCGTCTGTTGCCTGTGC
ACCTCGTACATGGGAAGCTGATGAAGCTAGGCGGCGCCGAGTCATTAAACGTTTGGCGCTGGAGCAAGAACGTATG
CGTAACGTTCTTGACGTTGGCGTCTATGACCAGGCGACATGGGAACAGGAGGACGCGCGCGATAATGAGTTCCTAA
CGGAACAATTAAACAATTTATATACTATTTATTCGATCGCTGAACGTTGTACGCGTCGACCTATTAAAGAGCACTC
TCCTATATCAGTTTCGAATAGGTTTGCTCCATTGGAATCCCTCAAGGTCGAGGTCGGTCAAGAAGCAGGCGAACTT
ACATTTAAGAAACCTAAATATACGCGCGTTTGCAAGAAAGTGAAGCGTGTTGCAACCCGCTTCGTTCGTGAAAAAG
TTGTTCGTCCTATGTGTTCTAGATCTCCTATGCTATTATTTAAGCTTAAGAAAATTATTTATGATTTGCACTTATA
TAGATTAAGAAAACAGATTAGGATTTTGAGACGTCAAAAACAGCGCGAGTATGAGTTAGAGTGTGTCACTAATCTG
TTACAATTATCGAATCCGGTGCAGGCAAAACCAGAGATGGATAACCCTAATCCAGGACCTGATGGCGAGGGTGAAG
TTGAATTAGAAAAGGATAGTAATGTTGTTTTAACAACTCAGCGAGATCCTAGTACATCTATTCCAGCGCCGGTGAG
CGTAAAATGGAGTAGATGGACTAGTAATGATGTAGTAGATGATTACGCCACAATCACATCTCGATGGTATCAGATT
GCTGAATTTGTTTGGTCGAAGGATGATCCATTTGATAAGGAGTTAGCACGTTTAATTTTGCCTCGTGCTTTGTTAT
CTAGTATAGAGGCTAATTCTGATGCTATATGTGATGTGCCTAATACCATTCCATTTAAGGTACACGCATATTGGCG
AGGCGATATGGAAGTTAGAGTTCAAATTAATTCAAATAAATTCCAAGTTGGTCAATTACAAGCTACTTGGTATTAT
TCGGATCATGAGAATTTGAATATATCGTCTAAGAGAAGCGTTTATGGATTTTCACAAATGGATCATGCTTTGATTA
GTGCGTCAGCAAGTAATGAAGCAAAATTGGTTATTCCATATAAGCATGTTTATCCATTTTTACCAACAAGAATTGT
GCCAGATTGGACTACTGGCATTTTAGATATGGGTGCTTTGAACATTCGTGTGATTGCTCCCTTACGGATGAGTGCT
ACTGGTCCAACTACCTGTAATGTCGTCGTGTTTATTAAATTAAATAACAGCGAGTTTACAGGACTTCCTCTGGTA

AGTTTTATGCGAGCCAAATCAAGGCAAAACCTGAGATGGATCGTATATTAAATTTAGCAGAAGGATTGTTGAATAA
CACGATTGGTGGTAATAATATGGATAATCCTTCTTATCAACAATCTCCTCGTCATTTTGTCCCGACTGGTATGCAC
AGCTTAGCTTTAGGTACTAATTTAGTTGAACCATTACATGCATTACGTTTGGATGCAGCCGGTACGACACAACATC
CTGTAGGTTGTGCTCCTGATGAAGATATGACTGTGTCTTCCATTGCATCTCGATATGGACTAATTAGACGGGTACA
ATGGAAGAAAGATCATGCTAAAGGATCGCTTTTGTTACAATTAGATGCTGATCCATTTGTGGAGCAAAGAATTGAA
GGTACGAATCCAATATCTTTGTATTGGTTCGCACCCGTGGGTGTAGTATCTAGTATGTTTATGCAATGGCGTGGTT
CATTAGAATATAGGTTTGATATTATAGCATCCCAATTTCATACTGGTAGGTTAATTGTAGGTTATGTGCCCGGTTT
GACAGCATCTTTGCAACTTCAAATGGACTATATGAAATTGAAGTCATCGAGTTATGTAGTATTTGATTTACAGGAA
AGTAATAGCTTCACTTTTGAGGTGCCATATGTTTCATATAGACCATGGTGGGTGCGTAAATATGGTGGCAATTATT
TGCCCTCGTCAACTGACGCTCCTAGTACATTATTCATGTATGTGCAGGTTCCGTTGATACCTATGGAAGCTGTTTC
AGATACTATTGATATCAATGTGTACGTACGGGGCGGTAGTTCATTTGAAGTTTGTGTTCCAGTCCAACCTAGTTTA
GGTTTGAATTGGAACACAGACTTTATTTTACGTAATGACGAAGAATACAGGGCTAAGACAGGTTATGCACCATATT
ATGCTGGAGTGTGGCATAGCTTTAATAATAGTAATTCTCTTGTTTTTAGGTGGGGATCTGCTTCTGATCAAATTGC
TCAGTGGCCGACAATTTCAGTACCAAGAGGTGAGTTAGCTTTCTTACGAATTAAGGATGGAAAGCAAGCTGCTGTA
GGAACTCAACCTTGGCGTACGATGGTTGTTTGGCCTTCTGGTCATGGCTATAATATTGGTATACCCACGTATAATG
CTGAACGAGCTCGCCAGCTTGCACAACACTTATATGGTGGTGGATCATTAACTGATGAGAAGGCCAAACAATTATT
TGTTCCTGCTAATCAACAAGGACCTGGTAAGGTAAGTAATGGAAATCCGGTATGGGAAGTCATGCGTGCACCATTG
TCAACACAGCGTGCGCATATGCAAGATTTTGAATTTATTGAGGCTATTCCAGAAGGAGAGGAGTCTCGTAATACTA
CAGTCTTGGATACGACTACTACTTTACAGTCGAGTGGGTTTGGTCGCGCCTTCTTTGGAGAAGCTTTTAATGATCT
TAAAACGTTAATGCGGCGATATCAATTATATGGTCAATTATTATTGTCCGTTACTACGGATAAGGATATTGATCAT
TGTATGTTTACCTTCCCTTGTTTACCACAAGGGTTAGCGTTAGATATTGGTTCTGCTGGCTCTCCACATGAAATCT
TTAATAGATGTCGTGATGGTATTATACCATTAATTGCATCTGGATATAGATTTTATAGAGGAGATTTGCGTTATAA
GATTGTTTTTCCAAGTAATGTTAATAGCAACATTTGGGTACAACATCGACCGGATCGTAGACTGGAAGGATGGTCC
GCGGCTAAGATTGTAAATTGTGATGCTGTGTCTACTGGTCAAGGAGTGTATAATCATGGTTATGCTAGTCACATTC
AAATCACGCGTGTAAATAATGTTATAGAATTGGAAGTTCCATTTTATAATGCTACTTGTTATAATTATTTACAGGC
GTTTAATGCGTCTAGCGCTGCATCTAGTTATGCAGTATCTTTAGGAGAAATATCGGTTGGTTTTCAAGCTACAAGT
GATGATATTGCATCTATCGTCAACAAACCTGTTACTATTTATTATAGTATTGGAGATGGTATGCAGTTTTCTCAGT
GGGTTGGATATCAACCGATGATGATCCTAGATCAGCTTCCTGCACCAGTAGTAAGGGCCGTGCCTGAGGGCCCTAT
TGCGAAGATTAAAAACTTCTTCCATCAAACAGCCGACGAAGTTAGAGAAGCTCAGGCAGCAAAGATGCGTGAAGAT
ATGGGTATGGTTGTTCAAGATGTTATTGGAGAACTTAGCCAGGCCATACCGGATCTTCAACAACCGGAGGTTCAAG
CAAATGTCTTCTCACTGGTGTCTCAGTTAGTGCATGCTATTATAGGTACTAGTTTGAAGACAGTAGCTTGGGCGAT
TGTTTCGATTTTTGTGACTTTAGGATTAATTGGACGCGAAATGATGCATTCAGTTATTACTGTAGTCAAGCGGTTA
CTTGAGAAATATCACTTGGCGACGCAACCCCAGGAATCCGCCAGTTCAAGTACTGTTATATCTGCCGTTCCAGAAG
CTCCCAATGCTGAAGCGGAGGAGGCAAGTGCTTGGGTATCCATTATTTATAATGGTGTGTGTAATATGCTTAATGT
GGCTGCTCAAAAACCGAAACAATTTAAAGATTGGGTAAAATTAGCTACTGTAGATTTTAGTAATAATTGTAGAGGT
AGTAATCAGGTATTTGTGTTTTTCAAGAATACATTTGAAGTGTTGAAGAAGATGTGGGGTTATGTATTTTGTCAGA
GTAATCCTGCAGCGCGTTTGTTAAAAGCTGTGAATGACGAGCCTGAGATTTTGAAAGCATGGGTGAAGGAATGTCT
GTATTTGGATGATCCCAAATTTAGAATGCGTCGAGCGCATGATCAAGAGTATATCGAGCGAGTGTTTGCGGCACAT
TCATATGGACAAATTTTGCTACATGATTTAACTGCTGAAATGAACCAATCACGAAATTTGAGTGTGTTTACACGTG
TGTATGATCAAATTTCAAAATTGAAGACTGATCTTATGGAAATGGGATCAAATCCATATATAAGGCGTGAATGTTT
TACGATATGCATGTGTGGTGCATCTGGAATTGGAAAATCATATTTGACCGATTCTTTATGTAGCGAGCTCTTACGT
GCGAGTCGTACTCCTGTGACAACGGGCATAAAATGTGTTGTTAATCCATTATCTGATTATTGGGATCAGTGTGATT
TTCAGCCTGTTTTGTGCGTTGATGATATGTGGAGTGTTGAAACATCTACTACGCTCGATAAGCAGTTGAACATGCT
TTTCCAGGTCCATTCACCTATCGTGCTTTCTCCTCCTAAAGCTGATTTAGAAGGTAAGAAAATGCGATATAACCCG
GAAATATTCATATACAATACGAATAAGCCTTTCCCGAGGTTTGATCGTATTGCTATGGAAGCTATTTATCGGCGTA
GAAATGTTTTGATTGAATGTAAAGCGAGTGAAGAGAAGAAGCGAGGATGCAAACATTGCGAGAACGATATTCCTAT
TGCTGAATGTAGTCCTAAGATGTTGAAAGATTTCCATCACATCAAATTTAGGTATGCACATGATGTATGTAATTCC
GAGACCACATGGTCTGAATGGATGACGTATAATGAATTTCTTGAATGGATAACTCCTGTGTATATGGCTAACCGTC
GTAAGGCGAATGAATCGTTTAAGATGCGTGTAGATGAAATGCAAATGTTACGTATGGATGAGCCATTGGAAGGTGA
TAATATCCTCAATAAGTATGTTGAAGTTAATCAGCGCTTAGTGGAGGAAATGAAGGCATTTAAGGAGCGTACATTA
TGGTCAGATTTACATCGTTTAGGTGCGGAAGTTAGTGCGTCAGTTAAGAAAGCTTTACCAACTATTTCCATAACTG
AAAAACTGCCACATTGGACTGTTCGATGTGGTATTGCTAAACCTGAGATGGATCATGCTTATGAGGTTATGAGTTC
GTATGCAGCTGGAATGAATGCAGAGATCGAAGCGCATGAACAAGTTCGGCGTTCATCAGTGGAATGTCAATATGCA
GAGCCTCAAGCTCCAAGAAATCCTGATGATGAAGGACCAACCATAGATGAAGAACTTATGGGCGACACTGAATTCA
CATCGCAGGCTCTAGAACGTCTTGTGGATGAAGGTTATATAACTGGAAAACAGAAGAAATATATAGCTACGTGGTG
TAGTAAGCGTCGTGAACATACTGCTGACTTTGATCTTGTGTGGACTGATAATTTGCGTGTGTTGAGCGCGTATGTG
CATGAACGTTCATCTTCAACTCGGCTTTCTACGGATGACGTCAAGTTATATAAAACGATTAGTATGTTACATCAAA
AGTATGATACCACAGAGTGTGCTAAATGTCAACATTGGTATGCTCCGTTGACTGATATCTATGTTGATGACAAGAA
ATTGTTCTGGTGTCAGAAAGAGAAAAAGACACTTATTGATGTCCGAAAATTGTCGAAAGAAGATGTGACTGTTCAA

TCGAAATTGATTAATTTATCTGTTCCTTGTAGTGAAGTATGTATGTTACATTCAAAATATTTCAATTATCTTTTCC
ATAAAGCATGGTTGTTCGAAAACCCAACTTGGCGCCTAATATATAATGGTACCAAGAAGGGTATGCCTGAGTACTT
TATGAATTGTGTAGATGAAATATCATTAGATTCCAAATTTGGTAAAGTGAAAGTATGGTTGCAAGCGATCATTGAT
AAGTATTTAACTCGTCCCGTGAAAATGATTCGTGATTTTCTTTTCAAGTGGTGGCCGCAAGTTGCGTATGTGTTGA
GCTTGCTAGGTATAATTGGTATAACTGCGTATGAAATGAGAAATCCGAAACCAACTTCTGAGGAATTAGCTGATCA
TTATGTGAATAGGCATTGTAGCTCTGATTTTTGGTCACCTGGACTGGCATCACCTCAAGGATTGAAATATAGTGAA
GCAGTAACAGTAAAGGCACCTAGAATCCATAGATTGCCGGTTACTACTAGGCCTCAAGGATCAACACAACAAGTAG
ACGCTGCTGTGAATAAAATTCTACAGAACATGGTTTATATTGGTGTTGTTTTCCCAAAAGTGCCTGGTAGTAAGTG
GCGAGATATTAATTTTAGGTGTCTTATGCTTCATAATAGGCAATGTTTAATGTTGAGGCATTATATCGAGTCAACT
GCCGCCTTTCCTGAGGGAACCAAGTACTATTTTAAGTATATTCATAATCAAGAGACTAGAATGTCTGGTGATATTT
CTGGTATTGAAATTGATTTGTTGAATTTGCCTAGATTGTATTATGGTGGTCTCGCGGGAGAGGAGTCGTTTGATAG
TAATATCGTGCTTGTGACTATGCCTAATCGTATTCCTGAGTGTAAGAGCATTATTAAATTTATAGCGTCACATAAT
GAACATATACGTGCTCAGAATGATGGAGTGTTAGTAACTGGCGACCATACTCAGCTATTGGCTTTCGAGAACAATA
ATAAAACTCCAATAAGTATTAACGCTGATGGCTTGTATGAGGTTATACTTCAAGGAGTATATACCTACCCATACCA
CGGCGATGGTGTTTGTGGTTCGATATTATTGTCTCGGAATTTACAACGGCCAATTATAGGTATCCATGTTGCTGGT
ACTGAAGGATTGCATGGCTTTGGAGTCGCTGAACCACTTGTACATGAAATGTTCACCGGTAAAGCGATTGAGAGTG
AAAGAGAGCCGTATGATCGTGTGTATGAACTTCCGTTGCGTGAATTAGATGAATCTGATATTGGTTTAGATACCGA
TTTATATCCGATTGGTAGAGTGGATGCAAAGCTAGCTCATGCTCAAAGTCCTTCTACTGGGATTAAAAAGACGCTT
ATCCATGGAACATTTGATGTAAGGACTGAACCAAATCCGATGTCATCACGTGATCCGAGAATAGCGCCGCACGATC
CTTTGAAGTTAGGGTGTGAAAAGCATGGCATGCCTTGTTCACCGTTTAATAGGAAACATCTGGAATTAGCGACAAA
TCATTTGAAAGAAAAATTAGTTTCAGTAGTTAAACCAATAAATGGTTGCAAGATTAGAAGTTTGCAAGATGCTGTA
TGTGGTGTGCCTGGATTAGATGGGTTTGATTCGATATCTTGGAATACTAGTGCTGGTTTTCCTTTGTCTTCATTAA
AGCCACCTGGAACATCGGGTAAGCGATGGTTGTTTGACATTGAGCTACAAGACTCGGGATGTTATTTCCTGCGTGG
AATGCGTCCCGAACTTGAGATTCAATTATCAACGACACAGTTAATGAGGAAAAAGGGAATAAAACCTCACACTATA
TTCACAGATTGTTTGAAAGATACTTGTTTGCCTGTTGAAAAATGTAGAATACCTGGTAAGACTAGAATATTTAGTA
TAAGTCCGGTACAGTTCACCATACCGTTTCGACAGTATTACTTAGACTTTATGGCATCCTATCGAGCTGCACGACT
TAATGCTGAGCATGGTATTGGTATTGATGTTAACAGCTTAGAGTGGACAAATTTGGCAACAAGGTTGTCTAAGTAT
GGCACTCACATCGTGACAGGAGACTATAAAAATTTTGGTCCTGGGTTAGATTCCGATGTTGCAGCTTCAGCGTTCG
AAATTATTATCGACTGGGTATTACATTACACTGAAGAAGATAATAAAGACGAAATGAAGCGAGTAATGTGGACCAT
GGCGCAAGAGATCTTAGCGCCTAGTCATCTATACCGCGATTTGGTGTACCGAGTACCTTGCGGAATTCCATCAGGT
TCTCCAATAACGGACATATTGAATACAATTTCAAATTGTTTGTTAATTAGGTTAGCTTGGTTAGGTATTACTGATT
TGCCTTTGTCCGAGTTCTCTCAAAATGTTGTTCTTGTCTGTTATGGCGATGATCTTATCATGAATGTTAGCGATAA
CATGATTGATAAGTTTAACGCCGTGACGATAGGAAAATTCTTTTCACAATATAAGATGGAATTTACGGATCAGGAT
AAATCAGGAAATACTGTAAAGTGGCGGACGTTACAGACTGCTACTTTCTTAAAACATGGGTTCTTAAAACATCCAA
CTAGACCTGTGTTTCTAGCTAACCTGGACAAGGTCTCGGTAGAAGGAACGACGAATTGGACTCATGCTCGAGGATT
GGGTCGTCGTACAGCAACCATAGAAAATGCTAAACAAGCATTAGAGTTAGCATTTGGGTGGGGTCCTGAATATTTT
AATTATGTCAGAAATACCATTAAAATGGCTTTTGACAAGTTGGGTATTTATGAGGACCTTATCACATGGGAAGAAA
TGGATGTTAGATGTTACGCTAGCGCGTAATATTATATTTTACATATTCATTAATTTTAATTTTATTTTAGGTTATT
GGAATTGAGGGAAGTACCACCCCCCAAGACCTTCGTTTTAAATCTACTAAGAGGAGTAAACCTATATATAAGAGTC
TAAAGACAGAGTGGATTAGACCATCATCTTTAGCTTATATATGGGGAAGGTTGAGTTGCCTCTAAAGACTCAGCTC
CATAGTAGAGTAGTTTTAATTACGATTAAAGTGGTACTCTAGGTTAGGTGTTACTCGCGTATTATCAACTAGTGGT
AATGCGTCCTAATTTTAGTATAGTTTTAACCATAATAGTAAAAAAAAAAAAAAAAAAAAAAAAAA

.

Supplementary Data S2. DWV, VDV1 and VDV1-DWV recombinant sequences generated by RT-PCR.

>VDV1-USA-MD-12-fragm-CP
TGAGTTAGAGTGTGTTACTAGTTTGCTACAGCTATCTAATCCTGTTTCAGCTAAACCTGAGATGGACAATCCTAAT
CCTGGTCCAGATGGTGAAGGTGAAGTTGAATTAGAAAAAGATAGTAATGTAGTATTAACTACACAACGTGATCCTA
GTACCTCTATTCCTGCTCCAACTAGTGTGAAGTGGAGTAGGTGGACTAGTAATGATGTTGTGGATGATTATGCCAC
TATAACTTCGCGTTGGTATCAGATTGCCGAATTTGTATGGTCAAAGGATGATCCATTTGATAAGGAATTGGCGCGC
TTAATTTTACCTCGAGCTTTGTTATCTAGTATTGAGGCTAATTCTGACGCTATTTGTGATGTACCTAATACTATTC
CGTTTAAGGTACATGCATATTGGCGTGGAGATATGGAAGTTCGAGTGCAGATTAACTCGAATAAATTCCAGGTTGG
TCAATTACAGGCAACTTGGTATTATTCGGATCATGAAAATTTGAATATTCAGACGAAGCGAAGTGTGTATGGTTTT
TCGCATATGGATCATGCTTTGATTAGTGCATCAGCGAGTAATGAAGCAAAATTAGTGATACCTTTTAAACACGTAT
ATCCATTCTTACCAACGCGCGTCGTTCCTGATTGGACAACTGGTATTCTTGATATGGGTACCTTAAATATTCGTGT
AATTGCTCCATTACGTATGAGTGCGACGGGACCAACCACTTGTAATGTTGTAGTATTTATTAAGTTAAATAATAGT
GAATTCACTGGTACTTCCTCTGGTAAGTTTTACGCGAATCAAATCAGGGCAAAACCTGAAATGGACCGTGTGTTAA
ATTTGGCAGAAGGATTATTAAATAATACTGTAGGTGGTTGTAATATGGATAATCCGTCATATCAGCAATCTCCGCG
TCATTTTGTTCCTACTGGTATGCATAGTTTAGCTTTAGGCACTAATTTAGTAGAGCCTTTGCATGCATTACGATTA
GATGCATCAGGTACAACACAACATCCAGTTGGGTGTGCGCCTGATGAAGATATGACTGTATCTTCCATTGCATCAC
GATATGGTTTAATT

>VDV1-USA-MD-12-fragm-C
GGTACGGTTATTTCCGCTGTTCCAGAAGCACCCAATGCTGAAGCAGAGGAGGCCAGTGCCTGGGTATCCATTATTT
ATAATGGTGTGTGTAATATGTTGAATGTAGCCGCTCAAAAACCGAAACAATTTAAAGATTGGGTAAAATTAGCTAC
CGTAGATTTTAGTAATAATTGTAGAGGTAGTAATCAGGTATTTGTGTTTTTCAAGAATACGTTTGAAGTGTTGAAG
AAAATGTGGGGTTATGTGTTTTGTCAGAGTAATCCTGCAGCGCGACTCTTGAAAGCAGTGAATGATGAACCTGAGA
TTTTAAAAGCGTGGGTTAAAGAATGTCTGTATTTAGATGATCCTAAATTTAGAATGCGACGTGCACATGATCAAGA
GTATATTGAGAGAGTGTTTGCGGCCCATTCGTATGGACAAATTTTATTGCATGACTTAACGGCTGAAATGAATCAA
TCGCGTAATTTAAGTGTGTTTACGAGAGTGTATGATCAAATATCTAAATTGAAGACGGATCTCATGGAAATGGGAT
CAAACCCATATATCAGGCGTGAATGTTTTACGATTTGTATGTGTGGTGCATCTGGAATTGGTAAGTCTTATTTAAC
TGATTCTTTATGCAGCGAGCTCTTACGTGCGAGTCGTACTCCAGTGACAACGGGCATTAAGTGTGTTGTGAACCCT
TTGTCTGATTATTGGGATCAGTGTGATTTTCAGCCCGTTTTATGTGTTGATGACATGTGGAGTGTTGAAACGTCTA
CTACGCTCGATAAACAGTTAAATATGCTATTTCAGGTTCATTCACCAATTGTACTTTCACCTCCTAAAGCTGATTT
AGAAGGTAAGAAAATGCGTTATAATCCTGAAATATTCATATATAATACGAATAAACCTTTTCCGAGGTTTGATCGT
ATAGCTATGGAAGCTATTTATCGACGTAGAAACGTTTTAATTGAATGTAAGGCTAATGAAGAGAAGAAGCGTGGAT
GTAAACATTGTGAGAATAATATACCCATTGCTGAATGTAGTCCAAAAATTTTGAAAGATTTTCATCACATTAAATT
TCGTTATGCTCATGATGTGTGCAATTCTGAAACTACGTGGTCTGAGTGGATGTCGTATAATGAATTTTTGGAATGG
ATTACTCCTGTATATATGGCTAATCGACGTAAAGCAAATGAATCGTTTAAGATGCGTGTTGATGAAATGCAAATGT
TGCGTATAGACGAGCCCTTGGAAGGCGATAATATTTTAAATAAGTATGTTGAAGTTAATCAGCGCTTAGTTGAGGA
AATGAAAGCTTT

>VDV1-USA-FL-22-fragm-C
CCGCCAATTCAGGTACGGTTATTTCCGCTATTCCAGAAGCACCCAATGCTGAAGCAGAGGAGGCCAGTGCCTGGGT
ATCCATTATTTATAATGGTGTGTGTAATATGTTGAATGTAGCCGCTCAAAAACCGAAACAATTTAAAGATTGGGTA
AAATTAGCTACCGTAGATTTTAGTAATAATTGTAGAGGTAGTAATCAGGTATTTGTGTTTTTCAAGAATACGTTTG
AAGTGTTGAAGAAAATGTGGGGTTATGTGTTTTGTCAGAGTAATCCTGCAGCGCGACTCTTGAAAGCAGTGAATGA
CGAACCTGAGATTTTAAAAGCGTGGGTTAAAGAATGTCTGTATTTAGATGATCCTAAATTTAGAATGCGACGTGCG
CATGATCAAGAGTATATTGAGAGAGTGTTTGCGGCCCATTCGTATGGACAAATTTTATTGCATGACTTAACGGCTG
AAATGAATCAATCGCGTAATTTAAGTGTGTTTACGAGAGTGTATGATCAAATATCTAAATTGAAGACGGATCTCAT
GGAAATGGGATCAAACCCATATATCAGGCGTGAATGCTTTACGATTTGTATGTGTGGTGCATCTGGAATTGGTAAG
TCTTATTTAACTGATTCTTTATGCAGCGAGCTCTTACGTGCGAGTCGTACTCCAGTGACAACGGGCATTAAGTGTG
TCGTGAACCCTTTGTCTGATTATTGGGATCAGTGTGATTTTCAGCCCGTTTTATGTGTTGATGACATGTGGAGTGT
TGAAACGTCTACTACGCTCGATAAACAGTTAAATATGTTATTTCAGGTTCATTCACCAATTGTACTTTCACCGCCT
AAAGCTGATTTAGAAGGTAAGAAAATGCGTTATAATCCTGAAATATTCATATATAATACGAATAAACCTTTTCCGA
GGTTTGATCGTATAGCTATGGAAGCTATTTATCGACGTAGAAACGTTTTAATTGAATGTAAGGCTAATGAAGAGAA
GAAGCGTGGATGTAAACATTGTGAGAATAATATACCCATTGCTGAATGTAGTCCAAAAATTTTGAAAGATTTTCAT
CACATTAAATTTCGTTATGCTCATGATGTGTGTAATTCTGAAACTACGTGGTCTGAGTGGATGTCGTATAATGACT

TTTTGGAATGGATTACTCCTGTATATATGGCTAATCGACGTAAAGCAAATGAATCGTTTAAGATGCGTGTTGATGA
AATGCAAATGTTGCGTATGGATGAGCCCTTGGAAGGCGATAATATTTTAAATAAGTATGTTGAAGTTAATCAGCGC
TTAGTTGAGGAAATGAAAGCTTTAAAGAGCGAA

>VDV1-USA-MD-12-fragm-POL
GCTTGGAATGGACAAACTTGGCAACAAGTCTGTCGAAATATGGCACGCATATTGTGACAGGAGATTATAAGAATTT
TGGTCCTGGGTTAGATTCTGATGTTGCCGCTTCAGCTTTTGAAATTATCATTGATTGGGTGTTAAATTACACTGAA
GAAGATGATAAAGACGAAATGAAGCGTGTAATGTGGACTATGGCTCAGGAAATCTTAGCTCCTAGTCACTTATGTC
GTGATTTAGTATATCGCGTGCCATGCGGTATTCCTTCTGGATCACCGATTACGGACATTTTGAATACTATTTCGAA
TTGTTTGTTAATTCGATTGGCTTGGCAAGGTATTACTGATTTGCCTTTATCCGAATTTTCTAGACATGTCGTGCTA
GTTTGTTACGGTGATGATCTCATCATGAATGTAAGTGATGAGATGATAGATAAATTCAATGCTGTAACAATTGGCG
ATTTCTTTTCGCGATATAAGATGGAATTTACGGATCAGGATAAGTCTGGAAATACAGTGCGGTGGCGAACTTTACA
AACTGCTACGTTTTTGAAGCATGGGTTCTTGAAACATCCAACAAGACCCGTGTTTCTAGCCAATCTGGATAAGGTT
TCTATAGAAGGAACAACCAATTGGACACATGCTCGAGGATTGGGTCGTCGAGTAGCAACCATTGAGAATGCTAAAC
AAGCGCTAGAGTTGGCATTCGGATGGGGTCCCGAATACTTCAATCATGTTCGGAATACCATTAAAATGGCATTCGA
CAAGTTAGGTATTTATGAGGATCTCATAACGTGGGAAGAAATGGATGTTAGATGTTATGCTAGTGCGTAATTTTAA
GATTTTAATACTCATTAAAATTAATTCATATTTAGGTTATTGGAATTGAGGGAAGTACCACCCCCCAAGACCTTCG
TTTTAAATCTGCTAAGAGGAGTGAACTTGCATATAAGAGTCTAAAAGCAGAGTGGATTAGACCACCACTTTTAGCT
TATATGTGAGGAAGGTTGAGTTGCCTCTAAAGACTCAGCTCCGTAGTAGAGTAGTTTTAGTTACGA

>Rec-VDV1-DWV-USA-MD-12-fragm-C
GGTACGGTTATTTCCGCTGTTCCAGAAGCACCCAATGCTGAAGCAGAGGAGGCCAGTGCCTGGGTATCCATTATTT
ATAATGGTGTGTGTAATATGTTGAATGTAGCCGCTCAAAAACCGAAACAATTTAAAGATTGGGTAAAATTAGCTAC
CGTAGATTTTAGTAATAATTGTAGAGGTAGTAATCAGGTATTTGTGTTTTTCAAGAATACGTTTGAAGTGTTGAAG
AAAATGTGGGGTTATGTGTTTTGTCAGAGTAATCCTGCAGCGCGACTCTTGAAAGCAGTGAATGATGAACCTGAGA
TTTTAAAAGCGTGGGTGAAAGAATGTCTGTATTTGGATGATCCTAAATTTAGAATGCGACGTGCACATGATCAAGA
GTATATTGAGAGAGTGTTTGCGGCCCATTCGTATGGACAAATTTTACTGCATGACTTAACTGCTGAAATGAATCAA
TCACGAAATTTGAGTGTGTTTACgaGAGTGTATGATCAAATATCTAAATTGAAGACTGATCTCATGGAAATGGGAT
CGAATCCATATATCAGGCGTGAATGTTTTACGATATGCATGTGTGGTGCATCTGGAATTGGAAAATCATATTTGAC
CGATTCTTTATGCAGCGAGCTCTTACGTGCGAGTCGTACTCCTGTGACAACAGGCATAAAATGTGTTGTGAATCCA
TTATCTGATTATTGGGATCAGTGTGATTTTCAGCCTGTTTTGTGCGTTGATGATATGTGGAGTGTTGAAACATCTA
CTACGCTCGATAAGCAGTTGAATATGCTTTTCCAGGTCCATTCCCCTATTGTGCTTTCTCCTCCTAAAGCTGATTT
AGAAGGTAAGAAAATGCGATATAACCCGGAAATATTCATATACAATACGAATAAACCTTTCCCGAGGTTTGATCGT
ATTGCTATGGAAGCTATTTATCGGCGTAGAAATGTTTTGATTGAATGTAAAGCGAGTGAAGAGAAGAAGCGAGGAT
GCAAGCATTGTGAGAATGATATTCCTATTGCTGAATGTAGTCCTAAGATGTTGAAAGATTTCCATCATATCAAATT
TAGGTATGCACATGATGTATGTAATTCCGAGACCACATGGTCTGAATGGATGACGTATAATGAATTTCTTGAATGG
ATAACTCCTGTGTATATGGCTAACCGTCGTAAGGCGAATGAATCGTTTAAGATGCGTGTAGATGAAATGCAAATGT
TACGTATGGATGAACCATTGGAAGGTGATAATATTCTCAATAAGTATGTTGAAGTTAATCAGCGTTTAGTGGAGGA
AATGAAGGCATTTAAGGAGCGTACACTATGGTCAGATTTACATCGCGTAGGTGCGGAAATTAGTGCGTCAGTTAAG
AAAGCTTTACCAACTATTTCTATAACCGAAAAACTACCACATTGGACTGTTCAATGTGGTATTGCTAAACCTGAGA
TGGATCATGCTTATGAGGTTATGAGTTCGTATGCAGCTGGAATGAATGCAGAGA

>Rec-VDV1-DWV-USA-MD-22-fragm-C
TTATTTCCGCTATTCCAGAAGCACCCAATGCTGAAGCAGAGGAGGCCAGTGCCTGGGTATCCATTATTTATAATGG
TGTGTGTAATATGTTGAATGTAGCCGCTCAAAAACCGAAACAATTTAAAGATTGGGTAAAATTAGCTACCGTAGAT
TTTAGTAATAATTGTAGAGGTAGTAATCAGGTATTTGTGTTTTTCAAGAATACGTTTGAAGTGTTGAAGAAAATGT
GGGGTTATGTGTTTTGTCAGAGTAATCCTGCAGCGCGACTCTTGAAAGCTGTGAATGACGAACCTGAGATTTTAAA
AGCGTGGGTGAAAGAATGTCTGTATTTGGATGATCCCAAATTTAGAATGCGACGAGCGCATGATCAAGAGTATATC
GAGAGAGTGTTTGCGGCCCATTCATATGGACAAATTTTGCTGCATGACTTAACTGCTGAAATGAACCAATCACGAA
ATTTGAGTGTGTTTACAAGAGTGTATGATCAAATTTCTAAATTGAAGACTGATCTTATGGAAATGGGATCAAATCC
ATATATAAGGCGTGAATGTTTTACGATATGCATGTGTGGTGCATCTGGAATTGGAAAATCATATTTGACCGATTCT
TTATGCAGCGAGCTCTTACGTGCGAGTCGTACTCCTGTGACAACGGGCATAAAATGTGTTGTTAATCCATTATCTG
ATTATTGGGATCAGTGTGATTTTCAGCCTGTTTTGTGCGTTGATGATATGTGGAGTGTTGAAACATCTACTACGCT
CGATAAGCAGTTGAACATGCTTTTCCAGGTTCATTCACCTATCGTGCTTTCTCCTCCTAAAGCTGATTTAGAAGGT
AAGAAAATGCGATATAACCCGGAAATATTCATATACAATACGAATAAGCCTTTCCCGAGGTTTGATCGTATTGCTA

TGGAAGCTATTTATCGGCGTAGAAATGTTTTGATTGAATGTAAAGCGAGTGAAGAGAAGAAGCGAGGATGCAAACA
TTGCGAGAACGATATTCCTATTGCTGAATGTAGTCCTAAGATGTTGAAAGATTTCCATCACATCAAATTTAGGTAT
GCACATGATGTATGTAATTCCGAGACCACATGGTCTGAATGGATGACGTATAATGAATTTCTTGAATGGATAACTC
CTGTGTATATGGCTAACCGTCGTAAGGCGAATGAATCGTTTAAGATGCGTGTAGATGAAATGCAAATGTTACGTAT
GGATGAGCCATTGGAAGGTGATAATATTCTCAATAAGTATGTTGAAGTTAATCAGCGTTTAGTGGAGGAAATGAAG
GCATTTAAGGAGCGTACATTATGGTCAGATTTACATCGTTTAGGTGCGGAAGTTAGTGCGTCAGTTAAGAAAGCTT
TACCAACTATTTCCATAACCGAAAAACTACCACATTGGACTGTTCAATGTGGTATTGCTAAACCTGAGATGGATCA
TGCTTATGAGGTTATGAGTTCGTATG

>Rec-VDV1-DWV-USA-MD-22-2-fragm-C

CTGTAGTTAAGCGGTTATTAGAAAAATATCACTTGGCGACGCAACCCCAGGAATCCGCCAATTCAGGTACGGTTAT
TTCCGCTATTCCAGAAGCACCCAATGCTGAAGCAGAGGAGGCCAGTGCCTGGGTATCCATTATTTATAATGGTGTG
TGTAGTATGTTGAATGTAGCCGCTCAAAAACCGAAACAATTTAAAGATTGGGTAAAATTAGCTACCGTAGATTTTA
GTAATAATTGTAGAGGTAGTAATCAGGTATTTGTGTTTTTCAAGAATACGTTTGAAGTGTTGAAGAAAATGTGGGG
TTATGTGTTTTGTTAGAGTAATCCTGCAGCGCGACTCTTGAAAGCAGTGAATGACGAACCTGAGATTTTAAAAGCG
TGGGTTAAAGAATGTCTGTATCTAGATGATCCTAAATTTAGAATGCGACGTGCGCATGATCAAGAGTATATTGAGA
GAGTGTTTGCGGCCCATTCGTATGGACAAATTTTACTACATGATTTAACTGCTGAAATGAATCAATCACGAAATTT
GAGTGTGTTTACACGTGTGTATGATCAAATTTCAAAATTGAAGACCGATCTTATGGAAATGGGATCGAATCCATAT
ATAAGGCGTGAATGTTTTACGATATGCATGTGTGGTGCATCTGGAATTGGAAAATCATATTTGACCGATTCTTTAT
GCAGCGAGCTCTTACGTGCGAGTCGTACTCCTGTGACAACAGGCATAAAATGTGTTGTTAATCCATTATCTGATTA
TTGGGATCAGTGTGATTTTCAGCCTGTTTTGTGCGTTGACGATATGTGGAGTGTTGAAACATCTACTACGCTCGAT
AAGCAGTTGAACATGCTTTTCCAGGTTCATTCCCCTATCGTGCTTTCTCCTCCTAAAGCTGATTTAGAAGGTAAGA
AAATGCGATATAACCCGGAAATATTCATATACAATACGAATAAGCCTTTCCCGAGGTTTGATCGTATTGCTATGGA
AGCTATTTATCGGCGTAGAAATGTTTTGATTGAATGTAAAGCGAGTGAAGAGAAGAAGCGAGGATGCAAGCATTGT
GAGAATGATATTCCTATTGCTGAATGTAGTCCTAAGATGTTGAAAGATTTCCATCATATCAAATTTAGGTATGCAC
ATGATGTATGTAATTCCGAGACCACATGGTCTGAATGGATGACGTATAATGAATTTCTTGAATGGATAACTCCTGT
GTATATGGCTAACCGTCGTAAGGCGAATGAATCGTTTAAGATGCGTGTAGATGAAATGCAAATGTTACGTATGGAT
GAACCATTGGAAGGTGATAATATTCTCAATAAGTATGTTGAAGTTAATCAGCGTTTAGTGGAGGAAATGAAGGCAT
TTAAGGAGCGTACGCTATGGTCAGATTTACATCGCTTAGGTGCAGAAGTTAGTGCGTCAGTTAAGAAAGCTTTACC
AACTATTTCCATAACCGAAAAACTACCACACTGGACTGTTCAATGTGGTATTGCTAAACCTGAGATGGATCATGCT
TATGAGGTTATGAGTTCGTATGCAGCAGGAATGAATGCAGAGATTGAAGCGCATGAACAAGTTCGGCGTTCATCAG
TGGAATGTCAATATGCAGAGCCTCAAGCTCCAAGAA

>DWV-USA-MD-12-fragm-C

CCAGTTCAAGTACTGTTATATCTGCCGTTCCAGAAGCTCCCAATGCTGAAGCGGAGGAGGCAAGTGCTTGGGTATC
CATTATTTATAATGGTGTGTGTAATATGCTTAATGTGGCTGCTCAAAAACCGAAACAATTTAAAGATTGGGTAAAA
TTAGCTACTGTAGATTTTAGTAATAATTGTAGAGGTAGTAATCAGGTATTTGTATTTTTCAAGAATACATTTGAAG
TGTTGAAGAAAATGTGGGGTTATGTATTTTGTCAGAGTAATCCTGCAGCGCGTTTGTTGAAAGCTGTGAATGACGA
GCCTGAGATTTTGAAAGCATGGGTGAAGGAATGTCTGTATTTGGATGATCCTAAATTTAGAATGCGTCGAGCGCAT
GATCAAGAGTATATCGAGAGAGTGTTTGCGGCACATTCATATGGACAAATTTTGCTACATGATTTAACTGCTGAAA
TGAATCAATCACGAAATTTGAGTGTGTTTACACGTGTGTATGATCAAATTTCAAAATTGAAGACTGATCTTATGGA
AATGGGATCGAATCCATATATAAGGCGTGAATGTTTTACGATATGCATGTGTGGTGCATCTGGAATTGGAAAATCA
TATTTGACCGATTCTTTATGCAGCGAGCTCTTACGTGCGAGTCGTACTCCTGTGACAACAGGCATAAAATGTGTTG
TTAATCCATTATCTGATTATTGGGATCAGTGTGATTTTCAGCCTGTTTTGTGCGTTGATGATATGTGGAGTGTTGA
AACATCTACTACGCTCGATAAGCAGTTGAACATGCTTTTCCAGGTCCATTCCCCTATCGTGCTTTCTCCTCCTAAA
GCTGATTTAGAAGGTAAGAAAATGCGATATAACCCGGAAATATTTATATACAATACGAATAAGCCTTTCCCGAGGT
TTGATCGTATTGCTATGGAAGCTATTTATCGGCGTAGAAATGTTTTGATTGAATGTAAAGCGAGTGAAGAGAAGAA
GCGAGGATGCAAGCATTGTGAGAATGATATTCCTATTGCTGAATGTAGTCCTAAGATGTTGAAAGATTTCCATCAT
ATCAAATTTAGGTATGCACATGATGTATGTAATTCCGAGACCACATGGTCTGAATGGATGACGTATAATGAATTTC
TTGAATGGATAACTCCTGTGTATATGGCTAACCGTCGTAAGGCGAATGAATCGTTTAAGATGCGTGTAGATGAAAT
GCAAATGTTACGTATGGATGAACCATTGGAAGGTGATAATATACTCAATAAGTATGTTGAAGTTAATCAGCGTTTA
GTGGAGGAAATGAAGGCATTTAAGGAGCGTACACTATGGTCAGATTTACATCGCGTAGGTGCGGAAATTAGTGCAT
CAGTTAAGAAAGCTTTACCAACTATTTCTATAACCGAAAAACTACCACATTGGACTGTTCAATGTGGTATTGCTAA
ACCTGAGATGGATCATGCTTATGAGGTTATGAGTTCGTATGCAGCTGGAATGAATGCAGAGA

>DWV-USA-FL-22-fragm-C
CCGCCAGTTCAAGTACTGTTATATCTGCCGTTCCAGAAGCTCCCAATGCTGAAGCGGAGGAGGCAAGTGCTTGGGT
ATCCATTATTTATAATGGTGTGTGTAATATGCTTAATGTGGCTGCTCAAAAACCGAAACAATTTAAAGATTGGGTA
AAATTAGCTACTGTAGATTTTAGTAATAATTGTAGAGGTAGTAATCAGGTATTTGTGTTTTTCAAGAATACATTTG
AAGTGTTGAAGAAGATGTGGGGTTATGTATTTTGTCAGAGTAATCCTGCAGCGCGTTTGTTAAAAGCTGTGAATGA
CGAGCCTGAGATTTTGAAAGCATGGGTGAAGGAATGTCTGTATTTGGATGATCCCAAATTTAGAATGCGTCGAGCG
CATGATCAAGAGTATATCGAGCGAGTGTTTGCGGCACATTCATATGGACAAATTTTGCTACATGATTTAACTGCTG
AAATGAACCAATCACGAAATTTGAGTGTGTTTACACGTGTGTATGATCAAATTTCAAAATTGAAGACTGATCTTAT
GGAAATGGGATCAAATCCATATATAAGGCGTGAATGTTTTACGATATGCATGTGTGGTGCATCTGGAATTGGAAAA
TCATATTTGACCGATTCTTTATGTAGCGAGCTCTTACGTGCGAGTCGTACTCCTGTGACAACGGGCATAAAATGTG
TTGTTAATCCATTATCTGATTATTGGGATCAGTGTGATTTTCAGCCTGTTTTGTGCGTTGATGATATGTGGAGTGT
TGAAACATCTACTACGCTCGATAAGCAGTTGAACATGCTTTTCCAGGTCCATTCACCTATCGTGCTTTCTCCTCCT
AAAGCTGATTTAGAAGGTAAGAAAATGCGATATAACCCGGAAATATTCATATACAATACGAATAAGCCTTTCCCGA
GGTTTGATCGTATTGCTATGGAAGCTATTTATCGGCGTAGAAATGTTTTGATTGAATGTAAAGCGAGTGAAGAGAA
GAAGCGAGGATGCAAACATTGCGAGAACGATATTCCTATTGCTGAATGTAGTCCTAAGATGTTGAAAGATTTCCAT
CACATCAAATTTAGGTATGCACATGATGTATGTAATTCCGAGACCACATGGTCTGAATGGATGACGTATAATGAAT
TTCTTGAATGGATAACTCCTGTGTATATGGCTAACCGTCGTAAGGCGAATGAATCGTTTAAGATGCGTGTAGATGA
AATGCAAATGTTACGTATGGATGAGCCATTGGAAGGTGATAATATCCTCAATAAGTATGTTGAAGTTAATCAGCGC
TTAGTGGAGGAAATGAAGGCATTTAAGGAGCGTACATTATGGTCAGATTTACATCGTTTAGGTGCGGAAGTTAGTG
CGTCAGTTAAGAAAGCTTTACCAACTATTTCCATAACTGAAAAACTGCCACATTGGACTGTTCGATGTGGTATTGC
TAAACCTGAGATGGATCATGCTTATGAGGTTATGAGTTCGTATGCAGCTGGAATGAATGCAGAGATCGAAGCGCAT
GAACAG
